# Carbon substrates utilization determine antagonistic fungal-fungal interactions among root-associated fungi

**DOI:** 10.3389/fmicb.2025.1645107

**Published:** 2025-08-14

**Authors:** Rosa Kemmerling, Louise-Elisabeth Dintilhac, Anouk Zancarini, Alice Mataigne, Christophe Mougel, Nathan Vannier

**Affiliations:** ^1^IGEPP, INRAE, Institut Agro, Le Rheu, France; ^2^IRISA, CNRS, Rennes, France

**Keywords:** plant microbiome, root-associated fungi, microbe-microbe interactions, carbon metabolism, fungal ecology, antifungal activity

## Abstract

**Introduction:**

The assembly of the plant microbiome results from a complex network of interactions. The role of microbial taxa in shaping the microbiome has recently gained attention, emphasizing the competitive dynamics and chemical warfare occurring within this dynamic environment. Within and around the roots, microbe-microbe interactions are piloted by nutritional constraints that can be modulated by the host. In this context, while nutrient blocking and antimicrobial production have largely been described as competitive traits in bacterial taxa, the importance of fungal metabolism in determining fungal-fungal interactions remains largely unexplored.

**Methods:**

In this work, we profiled the carbon substrate utilization of 91 root-associated fungal isolates from *Brassica napus* and *Triticum aestivum* and evaluated their antagonistic abilities against two agronomically relevant fungal competitors, *Rhizoctonia solani* and *Fusarium graminearum*.

**Results:**

Our results indicate that fungi arbor contrasted carbon utilization profiles and strategies that are independent from the two host plant species tested, the plant compartment and the geographic region. Strikingly, specific carbon utilization signatures were associated with antagonistic abilities with antifungal-mediated antagonism characterized by higher utilization rates of diverse carbon substrates while direct competitive abilities were associated with lower utilization rates of fewer carbon substrates.

**Discussion:**

Together with taxonomy-based predictions of antagonism-specific enzymatic reactions, these results suggest that carbon utilization profiles and enzymatic reactions prediction could be considered as markers of fungal antagonistic potential. From an ecological point of view, our results suggest that root-associated fungi have contrasted carbon usage strategies likely shaped by and determining fungal-fungal antagonistic interactions.

## 1 Introduction

The plant microbiota forms a complex and dynamic community of microorganisms that colonize both external surfaces and internal tissues of plants, including leaves and roots (Edwards et al., 2015; Trivedi et al., 2020; Peñuelas and Terradas, 2014). This assemblage encompasses a broad taxonomic diversity, comprising fungi, bacteria, archaea, oomycetes and protists (Buée et al., 2009; Vorholt, 2012). The balance of the plant microbiome is essential for plant health and fitness (Berendsen et al., 2012; Vandenkoornhuyse et al., 2015; Berg et al., 2021). As observed in the human microbiome, plant diseases are often associated with dysbiosis, characterized by an imbalance or loss of microbial diversity (Chen et al., 2020; Arnault et al., 2023). Understanding the ecological and molecular processes that govern microbiome assembly, − particularly the interplay between biotic interactions and abiotic constraints - has thus become a key challenge in harnessing microbiome functionalities for sustainable crop management (Trivedi et al., 2020). Microbiome assembly is shaped by multiple factors, including host genetic background and developmental stage (Oberholster et al., 2018; Toju et al., 2019; Wassermann et al., 2019), environmental conditions (Maestre et al., 2015; Zhang et al., 2019), and microbial interactions (Cordovez et al., 2019; Zhou et al., 2019; Fitzpatrick et al., 2020; Bai et al., 2022). Among the key modulators of this environment are root exudates, which consist of complex mixtures of carbon-based compounds secreted by plant roots (Hu et al., 2018; Ma et al., 2022). These exudates serve as a major source of organic carbon for soil microbes and account for a substantial proportion-up to 40%- of plant-fixed photosynthates (Grayston et al., 1997; Fransson et al., 2007; Gargallo-Garriga et al., 2018). They also play a pivotal role in shaping the rhizosphere microbiome by favoring specific microbial taxa (Haichar et al., 2008; Hugoni et al., 2018; Sun et al., 2023). The pivotal role of microbial interactions, especially those occurring between different kingdoms, in structuring community composition and functionality has been underscored by studies employing synthetic microbial communities (Agler et al., 2016; Niu et al., 2017; Durán et al., 2018).

While bacterial-fungal interactions and the role of bacterial metabolites in controlling fungal communities have been extensively documented (Durán et al., 2018; Pierce et al., 2021; Wolinska et al., 2021), fungal-fungal interactions remain comparatively underexplored. Although plant disease onset is often linked to total fungal biomass (Wolinska et al., 2021) or the presence of specific pathogens (Doehlemann et al., 2017), the influence of interspecific fungal interactions (i.e., antagonism), on community composition and disease dynamics is still poorly understood. These interactions can range from mutualism to antagonism and parasitism. In antagonistic interactions, several mechanisms have been described, illustrating the diverse strategies fungi employed to suppress competitors (Boddy and Hiscox, 2016). For instance, competitive exclusion has been described in endophytic colonization of tomato and cotton seeds by *Beauveria bassiana,* controlling root rot caused by *Rhizoctonia solani,* through direct competition for space (Ownley et al., 2008). Similarly, copiotrophic fungi that rapidly metabolize carbon-rich substrates can deprive pathogens of these resources (Ramirez et al., 2012; Männistö et al., 2016). Another major mechanism is mycoparasitism, wherein one fungus actively parasitizes another. This process requires direct contact, host recognition, adhesion, hyphal penetration, and enzymatic degradation of the host cell wall (Koch and Herr, 2021; Liu and Dong, 2023). For example, *Geotrichum* sp. parasitizes *Thanatephorus cucumeris*, using it as both a nutrient source and structural support (Donayre and Dalisay, 2016). Additionally, many fungi produce antifungal secondary metabolites-including flavonoids, alkaloids, terpenes, lipopeptides, and phenolic compounds-that inhibit the growth of fungal competitors (Mousa and Raizada, 2013; Lugtenberg et al., 2016). *Aspergillus*, *Penicillium*, *Fusarium*, and *Phoma* endophytes isolated from *Eleusine coracana* produce such compounds, effective against *Fusarium* spp. (Mousa et al., 2015). Thus, fungal-fungal competition encompasses a large range of interactions mechanisms from nutrient blocking and space occupation to the production of metabolites targeting competitors.

Because competition for resources is directly linked with nutrient availability and because the production of antifungal molecules requires specific precursors, it is likely that these fungal-fungal interactions are largely dependent on the nutritional environment. Fungi-fungi interactions are modulated by the plant’s surrounding physico-chemical environment, particularly by root exudates (Hu et al., 2018). Fungi capable of efficiently metabolizing exudate-derived compounds exhibit enhanced rhizosphere fitness (Broeckling et al., 2008), potentially leading to their selective enrichment (Hugoni et al., 2018). Importantly, exudate composition varies with plant species, genotype, and developmental stage (Kowalchuk et al., 2006; Chaparro et al., 2013; Burns et al., 2015; Eck et al., 2019). Consequently, the rhizosphere mycobiome is likely to reflect the composition and timing of exudation events. Fungal taxa may therefore compete for access to these resources (Sasse et al., 2018; Hu et al., 2020), and some may utilize specific carbon substrates to produce antifungal metabolites. This suggests that root-derived carbon inputs could serve as key drivers of fungal–fungal interactions and determine the antagonistic abilities of fungal taxa within a given environment. Considering the importance of fungal-fungal interactions for fungal fitness, it is also likely that these interactions sculpted fungal metabolic profiles over evolutionary time. In this context, we addressed the following questions: (i) Can different carbon utilization profiles be distinguished among fungal isolates depending on their ecological origins, such as plant compartment, host plant species and geographic region in France? (ii) Is there a carbon utilization profile associated with antagonistic isolates (i.e., antifungal-mediated antagonism and competition-mediated antagonism)? (iii) Is it possible to identify proteomic (i.e., EC) and metabolic (i.e., carbon substrates) markers predicting antagonistic effects? To explore these hypotheses, we used a collection of 91 fungal isolates associated with *Brassica napus* (oilseed rape) and *Triticum aestivum* (wheat) and assessed their antagonistic abilities against two widespread soil-borne plant pathogens, *Rhizoctonia solani* (*Rs*), and *Fusarium graminearum* (*Fg*). For all isolates of this collection we carried dual-culture assays against *Rs* and *Fg* to determine their antagonistic potential. As it is likely that the production of antifungal requires specific precursors, we characterized both competition-mediated antagonism (i.e., any form of competition reducing the growth of the competitor) and specifically antifungal-mediated antagonism (i.e., limitation of growth without direct contact). We identified competition-mediated antagonism through the reduction in growth of *Rs* and *Fg* in the presence of the 91 fungal isolates. Whenever an inhibition zone without contact between mycelia was observed it was considered as antifungal-mediated antagonism, assuming that a diffusible molecule inhibited the competitor growth. We profiled carbon utilization capacities and predicted enzymatic reaction profiles (i.e., predicted proteomic models) based on taxonomic affiliation. We identified antifungal-mediated antagonism measured by the formation of inhibition zones and competition-mediated antagonism measured by the growth reduction of competitors (i.e., *Rs* or *Fg*). Antagonistic abilities were then compared to carbon utilization profiles and predicted proteomes to investigate the determinants of antagonistic fungal-fungal interactions.

## 2 Materials and methods

### 2.1 Fungal material and selection of fungal isolates

The fungal isolates used were isolated in 2021. Briefly, root and rhizosphere samples were collected from 48 oilseed rape and 50 wheat fields across three agriculturally distinct regions of France (East, South and West). Samples were ground and plated on three different culture media: Inhibitory Mold Agar (IMA) (Scognamiglio et al., 2010), Rose-Bengal Chloramphenicol Agar (RBCA; SIGMA-ALDRICH; Ref. 17,211-500G) and Potato-Carrot Agar (PCA) (Chelkowski et al., 1992). All isolates were identified by amplification of the ITS1 region using universal primers ITS1ngs (5’-GGTCATTTAGAGGAAGTAA-3′) and ITS2ngs (5’-TTYRCKRCGTTCTTCATCG-3′). Amplicons were sequenced by Macrogen Europe using Sanger sequencing method. For identification of fungal isolates, the *ITS1* sequences obtained were compared with those in the nucleotide database using the Basic Local Alignment Search Tool (BLAST) of the National Center for Biotechnology Information (NCBI) in the USA (accessed April 2022).

The diversity of the isolates was illustrated through the construction of a phylogenetic tree, which was obtained by aligning the sequences using the MAFFT software (Katoh and Standley, 2013; Kuraku et al., 2013; Katoh et al., 2018) with default parameters to optimize sequence alignment. The phylogenetic tree was then generated using RAxML software (version 1.2.2; Stamatakis, 2014), applying the GTRCAT model (Stamatakis, 2014) and 100 bootstrap replicates to assess branch support. To adequately represent the observed diversity, we used PARNAS (Markin et al., 2023), a selection algorithm based on phylogenetic coverage. This software uses the phylogenetic distances on a phylogenetic tree and optimizes the selection of isolates in order to cover all the diversity represented on the tree. A total of 40 wheat isolates and 51 oilseed rape isolates were selected from the entire phylogenetic tree, thus covering the entire taxonomic range at genus level. To validate the representativeness of the selected isolates in relation to the entire crop collection, we used the same PARNAS software (Markin et al., 2023) this time quantifying the proportion of phylogenetic diversity captured by the selected isolates, enabling us to assess the extent to which these isolates reflect the overall diversity of the collection.

The taxonomic classification of these isolates was validated by a second round of sequencing, using the ITS1ngs and ITS2ngs primers described above. To further assign the isolates studied, additional primers NS1 (5’-GTAGTCATGCTTGTCTC-3′) and NS4 (5’-CTTCCGTCGAATTCTTAA-3′) were also added using the same protocol. The resulting sequences were sent for sequencing to Macrogen, and taxonomic identification was validated using BLASTn against the NCBI nucleotide database (accessed October 2023).

Because NS1/NS4 barcodes, which amplify the 18S region of rRNA, are more conserved than the ITS1 region, we chose to represent the genetic diversity of isolates by forming a phylogenetic tree with NS1/NS4 barcodes. All the phylogenetic trees represented in this article are based on these barcodes, using the following method. These sequences were aligned using Seaview (version 4, Gouy et al., 2010) with the default parameters. Phylogenetic trees were constructed using Kimura’s method with the same software.

### 2.2 Antagonistic capacities against *Rs* and *Fg*

The competitive mediated antagonistic ability and the antifungal-mediated antagonistic ability of 91 fungal isolates was tested against two known plant pathogens, *Rs* and *Fg*. The fungal pathogens *Fg* (strain MDC_Fg1) and *Rs* (strain AG 2–1 R5) were kindly provided from previous work (Gilligan and Bailey, 1997; Alouane et al., 2021). *Rs* and *Fg* cause damping-off on oilseed rape and wheat, respectively. We tested whether the 51 fungal isolates collected from oilseed rape could limit the growth of *Rs* through competitive mediated antagonism or antifungal-mediated antagonism using a modified dual-culture antagonism assay, as described in Zhao et al. (2021). We used the same approach to test whether the 40 fungal isolates collected from wheat could limit the growth of *Fg*. Briefly, all fungal isolates (i.e., those isolated from oilseed rape and wheat, and the competitors *Rs* and *Fg*) were grown for 7 days at 20°C on IMA medium (Inhibitory Mold Agar). For the dual-culture confrontation tests, a 5 mm diameter mycelial plug was collected from the growing edge of each fungal colony. Two plugs from two fungi (one isolate and *Rs* or *Fg*) were placed on IMA medium in 90 mm Petri dishes facing each other, 6 cm apart (center-to-center), each positioned 1 cm from the edge of the plate. After 7 days of incubation at 20°C, the Petri dishes were scanned (Epson Perfection V37), and the growth area (in cm^2^) of both the pathogen and the fungal isolate was measured using ImageJ Fiji software (version 1.54i; Schindelin et al., 2012). Plates inoculated only with *Rs* or *Fg* were included as controls to compare the pathogen’s growth in isolation with its growth in the presence of the different isolates. All experimental conditions, including controls and confrontation assays, were performed in triplicate, and the entire experiment was independently repeated three times. The inhibition rate was calculated using the following formula: Inhibition rate = 1 - (A1/A2), where A1 is the area occupied by the competitor (i.e., *Rs* or *Fg*) in the presence of a tested isolate, and A2 is the average area occupied by the competitor alone (i.e., control plates). Thus, a high inhibition rate translates to a high reduction in competitor growth area.

The ability of each isolate to perform competition-mediated antagonism was evaluated across all isolates tested and consisted of assessing the difference in growth (i.e., inhibition rate) of *Rs* and *Fg* in the presence of different isolates. Whenever a zone without mycelium was observed between the two mycelial growth areas (i.e., no visible mycelial growth of the pathogen or the tested isolate), we considered that the tested isolate is an antifungal-mediated antagonistic isolate. To validate that the growth measurements are robust and persist after 7 days, the growth was measured a second time at 28 days after inoculation for 39 isolates.

### 2.3 Profiling of fungal isolates carbon utilization profiles

The ability of each isolate to metabolize 95 different carbon substrates was evaluated using specific filamentous fungal (FF) microplates from Biolog (Biolog™ FF MicroPlates, Biolog Inc., Hayward, CA, USA; Supplementary Table S1). This method enables the analysis of fungal functional diversity and catabolic versatility (Pinzari et al., 2016). The inoculation procedure followed the manufacturer’s recommendations (Biolog FF MicroPlate™ Protocol, Pawlik et al., 2019) with minor modifications. Isolates were grown for 7 days at 20°C in the dark on 20% malt extract agar. Mycelia were harvested by adding 2 mL of FF-IF (filamentous fungal inoculation fluid) inoculation fluid (Biolog™) directly into the culture plates and gently scraping the surface with a sterile loop. The recovered suspension was then vortexed with 1 mm sterile glass beads to obtain a homogeneous solution used as inoculum. The optical density at 600 nm (OD₆₀₀) of the inoculum was adjusted to 0.002, corresponding to 99% transmittance using a spectrophotometer (Jenway™ 7,300 spectrophotometer). Each well of a Biolog FF MicroPlate was filled with 100 μL of the inoculum, and each isolate was tested in triplicate to ensure reproducibility of the carbon utilization profile of each isolate. The carbon utilization profile was measured by detecting the reduction of a tetrazolium dye, monitored via changes in optical density at 590 nm (OD₅₉₀), at 30-min intervals over 168 h using the OmniLog™ instrument (Biolog™), controlled via Data Analysis software (Version 1.7, Biolog Inc.). The area under the curve (AUC) was calculated from the kinetic data to quantify metabolic activity on each substrate. AUC values were then normalized by subtracting the signal of the negative control well (without carbon source) to obtain a relative consumption rate, which was used in downstream analyses.

### 2.4 Identification of proteomic features associated with antagonistic capacities

The consensus proteome of all isolates was analyzed to identify Enzyme Commission (EC) numbers specific to isolates with competition-mediated antagonism of both competitors. For this purpose, isolates metabolism was estimated using EsMeCaTa (Belcour et al., 2022), a Python workflow that approximates metabolic potential based on taxonomic affiliation by retrieving and analyzing reference proteomes from the UniProt database.

First, for the entire taxonomic assignment of the selected fungi, EsMeCaTa searches for at least 5 reference proteomes associated with each taxonomic assignment, regardless of the rank at which the isolate was identified. If fewer than 5 reference proteomes are found, EsMeCaTa moves to higher taxonomic ranks (up to family level only) and explores non-reference proteomes to complete the set. EsMeCaTa then identifies proteins shared among the retrieved proteomes. Only proteins found in at least 50% of these proteomes are retained. Finally, EsMeCaTa constructs a consensus sequence for each retained protein and calls EggNOG-mapper (Cantalapiedra et al., 2021) to annotate the retained proteins with EC numbers. These EC annotations were grouped based on whether the isolates are showing competition-mediated antagonism against *Rs* or *Fg* (as defined in section 2.3) or not. Isolates with the same taxonomic assignment and/or reference proteomes that were present in competitive and non-competitive isolates were excluded. This allowed us to identify ECs uniquely associated with inhibitors.

### 2.5 Assessing the specificity of fungal isolates antagonistic abilities against two fungal competitors

In order to determine whether the observed inhibition potential of the isolates tested (i.e., general competition-mediated antagonism or antifungal-mediated antagonism) are competitor-specific (i.e., specific to *Rs* or *Fg*) we conducted additional confrontation assays including isolates displaying antagonistic phenotypes against *Rs* (Oilseed rape isolates) or against *Fg* (Wheat isolates). To do so, we followed the same protocol as previously described (part 2.2 Antagonistic capacities against *Rs* and *Fg*) using a total of 25 isolates displaying inhibition phenotypes in the previous tests. Three biological replicates and 3 technical replicates were performed for each confrontation.

### 2.6 Statistical analyses

All statistical analyses were carried out using R version 4.3.3 within RStudio version 2024.12.0. To assess differences between fungal isolates in terms of competition-mediated antagonism against the two competitors, we first evaluated the normality of *Rs* and *Fg* growth surface distributions. Given that these distributions did not meet the assumptions of normality, non-parametric Kruskal-Wallis tests were employed (base R stats package), followed by Dunn’s *post hoc* tests with false discovery rate (FDR) correction (rstatix package, Kassambara, 2021).

In order to evaluate the link between the observed antagonistic phenotypes and the carbon utilization profiles we grouped isolates into phenotypic categories. To do so, we used the unsupervised k-means clustering classification method. This method allows observations to be grouped into an optimal number of clusters based on their similarity. By applying this approach to the observed inhibition rates (Supplementary Figure S1), the analysis identified the two distinct groups for both oilseed rape and wheat isolates. Based on the distribution of the inhibition values and on the k-means clustering we thus selected a 0.3 inhibition rate threshold for oilseed rape isolates and 0.2 for wheat isolates (Supplementary Figure S1). The inhibition rate was thus used as both a quantitative variable in linear models (lm function, stats package) and analysis of variance (ANOVA) using the rstatix packages and as a qualitative variable (i.e., competitive vs. non-competitive isolates) in subsequent Kruskal-Wallis tests followed by Dunn’s post hoc tests with FDR correction. These analyses enabled us to examine the influence of ecological origin, including the geographic region in France, plant compartment, and host plant species (wheat or oilseed rape). A linear regression was also performed between the inhibition rate calculated at 7 days post-inoculation and at 28 days post-inoculation.

In parallel, the carbon utilization profile of each isolate was assessed using carbon sources in Biolog FF microplates. More specifically, the area under the consumption curve (AUC) values for each well were calculated and adjusted relative to the control well (i.e., AUC of each well divided by AUC of well A01). This data was used to form a heatmap using the ggplot function in the ggplot2 package, and to create a Euclidean distance matrix using the dist function in the stats package. This matrix was then used to explore the influence of multiple variables, such as ecological origin (i.e., geographic region in France, plant compartment, and host plant species), taxonomic assignment, and competition-mediated antagonistic as a qualitative variable (against *Rs* or *Fg*), on the carbon utilization profiles of the isolates by performing a Permutational Multivariate Analysis of Variance (PERMANOVA) analysis using the adonis2 function in the vegan package (version 2.6–4; Oksanen et al., 2013). At the same time, in order to highlight the differences in carbon utilization between the antagonistic isolates, three analyses (i.e., PCA, Random forest and PPLS-DA) were carried out to obtain the most reliable results possible. PCAs were performed using the rda function in the vegan package to highlight the most discriminating carbonaceous substrates on the first two principal axes, applying an absolute contribution threshold > 0.8. The Random Forest analysis (Breiman, 2001) was performed using the MUVR package (version 0.0.976; Shi et al., 2019) to identify the major carbon substrates that predict the competition-mediated antagonistic as a qualitative variable (competitive isolates vs. non-competitive isolates) of the 51 fungal isolates obtained from oilseed rape isolates and tested against *Rs*. The MUVR algorithm achieves a minimal feature selection by performing recursive variable elimination in a repeated double cross-validation procedure and improves predictive performance minimizing over-fitting and false positives. In this analysis, we used the following parameters for the random forest analysis: 48 repetitions, 8 outer cross-validation segments and 80% of variables kept for iteration of the recursive variable elimination in the inner loop. The statistical significance of the ‘max’ model (i.e., model including predictors with redundant but not erroneous information) was assessed with 100 permutations on the competition mediated antagonistic qualitative variable. Finally, a PPLS-DA was used to discriminate groups of fungal isolates during competition-mediated antagonism on the basis of their carbon utilization profiles. PPLS-DA consists of a partial least square’s regression analysis where the response variable is a qualitative variable (y-block; describing the grouping factor), expressing the class membership of the statistical units (Sabatier et al., 2003). Utilization rates of the 95 carbon sources by the fungal isolates were first scaled by Z-score normalization before using the cppls function in the package pls. The PLS-DA procedure includes a cross-validation step producing a *p* value that expresses the validity of the PLS-DA method regarding the dataset (function MVA.test in package RVAideMemoire). The PLS-DA procedure also expresses the statistical sensitivity, indicating the modelling efficiency in the form of the percentage of misclassification of samples in categories accepted by the class model (function MVA.cmv in package RVAideMemoire). These three methods therefore identify carbon substrates used differently in antagonistic isolates. Only the carbon substrates identified in the three analyses were subsequently studied. Non-parametric Kruskall-Wallis tests and linear models (lm function) were used to identify differences in the consumption of these carbonaceous substrates between antagonistic and non-antagonistic isolates.

## 3 Results

### 3.1 Selection of a representative set of fungal isolates associated to oilseed rape and wheat roots

To investigate antagonistic fungal-fungal interactions, we used a previously established collection of 780 fungal isolates, comprising 209 from wheat and 571 from oilseed rape, collected in 2021 in fields from three main agricultural regions in France. From this initial collection, we assembled a subcollection by selecting representative isolates. Using Parnas (Markin et al., 2023), we identified sequence-based diversity clusters and selected 91 representative isolates (40 from wheat and 51 from rapeseed; Supplementary Table S2) based on their genetic diversity (i.e., ITS1 sequences). These isolates represent 78.98% of the 780 isolates sequence diversity. The majority of the selected isolates from oilseed rape are Eurotiomycetes (*n* = 20; 39.2%), Sordariomycetes (*n* = 12; 23.5%), and *Mucoromycetes* (*n* = 8; 15.6%), while the one from wheat are mainly classified as Eurotiomycetes (*n* = 14; 35%), Sordariomycetes (*n* = 13; 32.5%) and Dothideomycetes (*n* = 9; 22.5%).

### 3.2 Antagonistic abilities of individual fungal isolates

**Figure 1:**
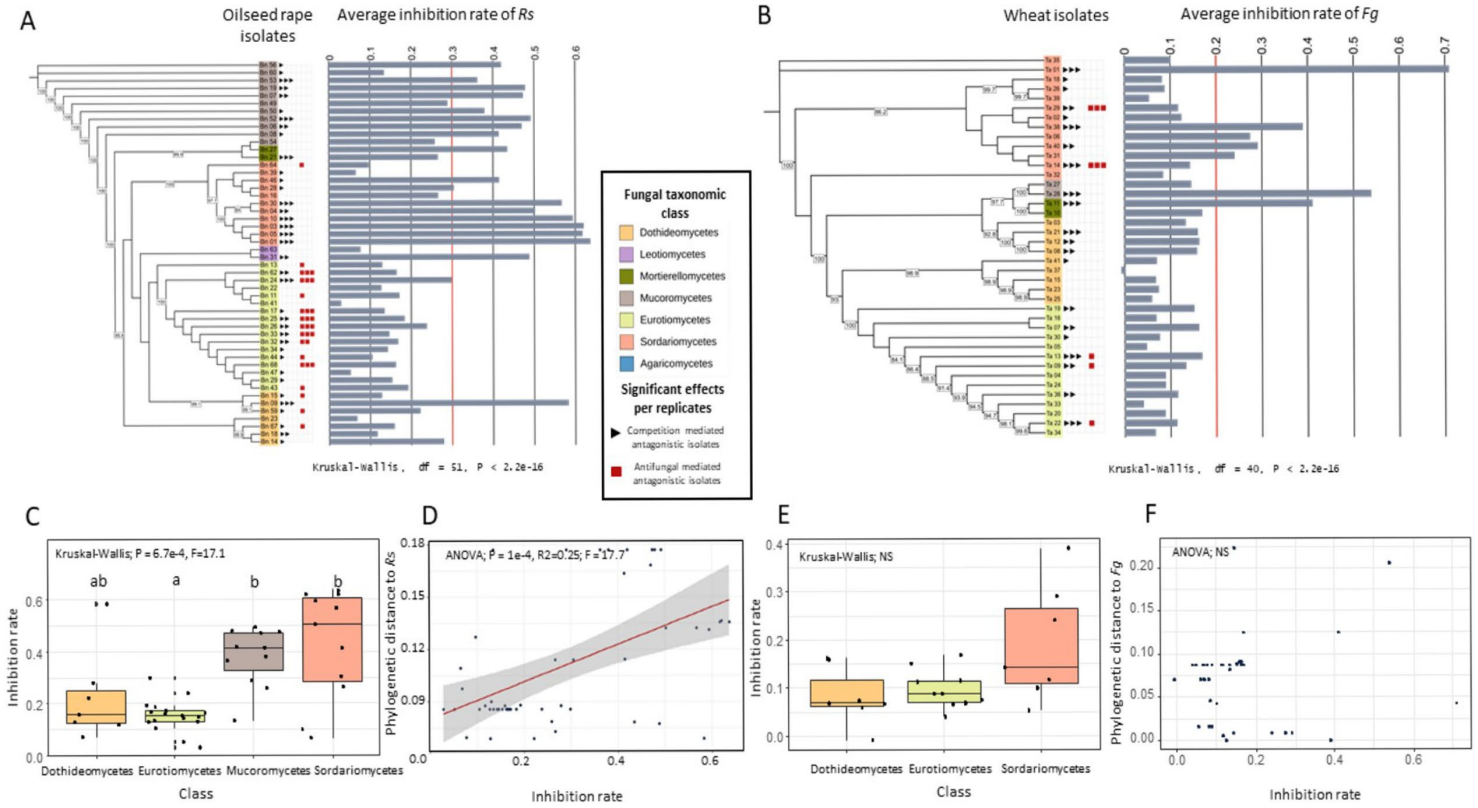
Inhibition of two fungal competitors (i.e., *Rs* and *Fg*) by different fungal isolates. **(A,B)** Maximum likelihood tree of the fungal isolates based on SSU sequences, showing phylogenetic relationships among fungal isolates and their average inhibition rates against *Rhizoctonia solani* (*Rs*) **(A)** and *Fusarium graminearum* (*Fg*) **(B)**. These isolates were either isolated from oilseed rape or wheat. Oilseed rape isolates were tested against *Rs* and wheat isolates against *Fg*. Fungal classes are color-coded. Bars indicate average inhibition rates across replicates, with a red line marking the limit between competitive isolates and non-competitive isolates. Red and black squares next to each isolate denote significant antagonistic mechanisms in a biological replicate. Kruskal-Wallis tests reveal significant differences in inhibition rates among isolates for both pathogens (*p* < 2.2e−16). **(C,E)** Inhibition rates of each fungal class against *Rs* **(C)** and *Fg* **(E)**. Significant differences were observed for *Rs* (Kruskal-Wallis, *p* = 0.00067), but not for *Fg* (*p* = 0.13). Different letters indicate statistically significant groups (post-hoc test). **(D,F)** Linear regressions showing the relationship between inhibition rate and phylogenetic distance from *Rs* **(D)** and *Fg* **(F)**. A significant positive correlation was observed for *Rs* (ANOVA, *p* = 0.0001, R^2^ = 0.25), but no significant relation was found for *Fg.*

To determine the antagonistic potential of individual isolates, we carried out *in vitro* dual confrontation tests against two competitors, *Rs* and *Fg*. Oilseed rape isolates were tested against *Rs*, while wheat isolates were tested against *Fg*. The inhibition rate of isolates was analyzed in relation to their ecological origin, including the geographic region in France and plant compartment (i.e., rhizosphere vs. roots), as well as their taxonomic classification. Across all dual confrontation assays, regardless of competitor, the two main antagonistic mechanisms were identified: (i) competition-mediated antagonism, and (ii) antifungal-mediated antagonism (Figures 1A,B; Supplementary Figures S2–S4).

We first examined the competition-mediated antagonism between the competitor and the isolates by examining the inhibition rate of the different fungal isolates. Among the 51 oilseed rape isolates tested against *Rs*, we observed significant differences in *Rs* growth depending on the isolate (Kruskal-Wallis chi-squared = 423.46, df = 51, *p* < 0.05; Figure 1A; Supplementary Figure S3), with a mean inhibition rate of 28% (+/− 18%). Across the three experimental replicates, 10 out of 51 isolates significantly reduced *Rs* growth (Kruskal-Wallis, *p* < 0.05; Figure 1A). These isolates belonged to the fungal classes Mucoromycetes, Mortierellomycetes, Sordariomycetes, Eurotiomycetes, and Dothideomycetes. Interestingly, similar patterns were observed for the 40 wheat-derived isolates tested against *Fg* (Figure 1B). *Fg* growth varied significantly among the isolates (Kruskal-Wallis chi-squared = 303.91, df = 40, *p* < 2.2e-16; Figure 1B; Supplementary Figure S3), with a mean inhibition rate of 15% (+/− 14%). Likewise, across three experimental repetitions, 7 out of 40 isolates significantly inhibited *Fg* growth (Kruskal-Wallis, *p* < 0.05; Figure 1B). These competitive isolates also belonged to the same fungal classes identified in the *Rs* assays: Sordariomycetes, Mucoromycetes, Mortierellomycetes, Dothideomycetes, and Eurotiomycetes. We then tested whether these inhibition patterns can be connected to the fungal taxonomy. As only two isolates from Leotiomycetes and Mortierellomycetes were present among the oilseed rape isolates, and a similar scarcity was observed for Mortierellomycetes and Mucoromycetes among the wheat isolates, these taxonomic groups were excluded from these analyses. For the oilseed rape isolates tested against *Rs*, a significant difference in inhibition rate among taxonomic classes was detected (Kruskal-Wallis; *p* = 0.00067, chi-squared = 17.1; Figure 1C). Eurotiomycetes showed the lowest average inhibition rate (15% +/− 7%), whereas Mucoromycetes and Sordariomycetes displayed significantly higher average inhibition rates of 38% (+/− 11%) and 42% (+/− 21%) on average, respectively (Dunn’s test with FDR correction; *p* < 0.05; Figure 1C). In contrast, no such difference in inhibition rate was observed among taxonomic classes for isolates tested against *Fg*, particularly among Dothideomycetes, Eurotiomycetes, and Sordariomycetes (Dothideomycetes, Eurotiomycetes, and Sordariomycetes; Kruskal-Wallis, *p* > 0.05, Figure 1E). However, it is important to note that the Mucoromycetes, identified as competitive isolates against *Rs* for oilseed rape isolates, could not be tested for wheat due to low statistical power.

Interestingly, oilseed rape isolates ability to reduce *Rs* growth was significantly correlated with phylogenetic distance to *Rs* (ANOVA; *p* = 0.0001, R^2^ = 0.25; *F* = 17.7; Figure 1D), with distant isolates yielding stronger inhibition rate than related isolates. In contrast, the phylogenetic distance between wheat isolates and *Fg* did not correlate with their inhibition abilities (ANOVA; p > 0.05, Figure 1F).

In addition, we investigated the ability of fungal isolates to perform antifungal-mediated antagonism, by observing the presence or absence of inhibition zones in dual culture assays. Antifungal mediated antagonism was observed for seven isolates tested against *Rs* and two isolates tested against *Fg* across all three experimental replicates (Figures 1A,B). All isolates that showed a zone of mycelium growth inhibition and were therefore identified as exhibiting antifungal-mediated antagonism against *Rs,* belonged to the taxonomic class Eurotiomycetes, specifically the genus *Penicillium* (Figure 1A; Supplementary Figure S4), while isolates identified as performing antifungal-mediated antagonism against *Fg* were affiliated with the Sordariomycetes (Figure 1B; Supplementary Figure S4). These results suggest that these isolates may produce antifungal compounds and effectively suppress the growth of *Rs* and *Fg.*

To determine whether observed antagonistic phenotypes persisted over time, a second growth measurement was performed 28 days after inoculation. A significant correlation of the inhibition rate was observed between the two dates (day 7 and day 28) for both wheat isolates tested against *Fg* (ANOVA, *p* < 0.05, R2 = 0.85, *F* = 118.9) and rapeseed isolates tested against *Rs* (ANOVA, *p* < 0.05, R2 = 0.77, *F* = 54.56; Supplementary Figure S5). The inhibition zones formed by the different isolates were also present at 28 days post-inoculation (Supplementary Figure S6) except for one isolate (Ta_14).

### 3.3 Carbon utilization profile of individual fungal isolates

To investigate the diversity of carbon utilization profiles among fungal isolates, we analyzed their utilization of 95 distinct carbon substrates. We first aimed at exploring potential associations between metabolic traits and isolates’ origin (i.e., geographic region, compartment and host plant species), as well as taxonomic classification.

**Figure 2:**
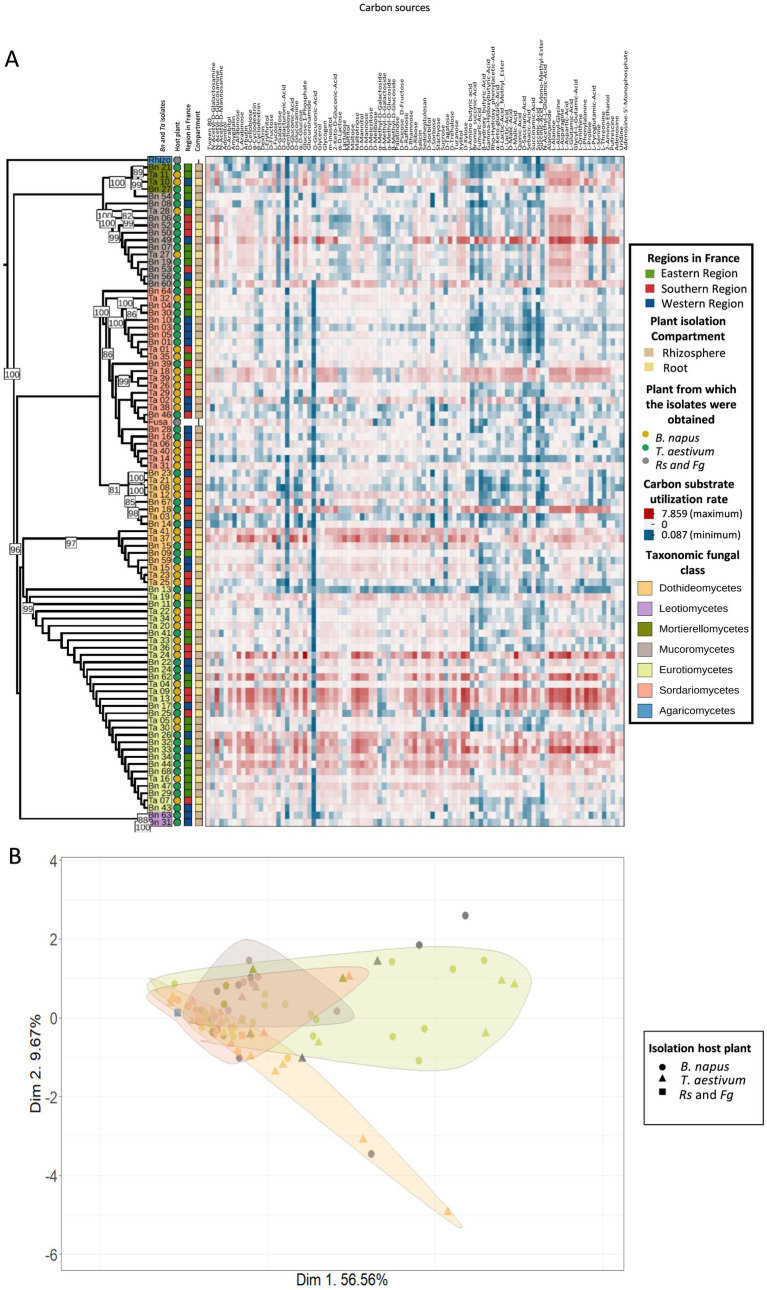
Carbon utilization profiles of fungal isolates across host plant species, geographic regions in France, plant compartment and taxonomic assignment. **(A)** Heatmap showing the carbon utilization profiles (Biolog FF microplates) of fungal isolates grouped by hierarchical clustering. Each row represents a fungal isolate and each column a specific carbon substrate. All carbon sources and the corresponding values are included in Supplementary Table 5. Colors indicate the consumption rate, with red indicating high and blue indicating low usage. Metadata annotations on the left indicate the region of origin in France (i.e., East, South, West), plant compartment (i.e., Rhizosphere, Root), host plant [i.e., oilseed rape, wheat, or *R. solani* (*Rs*) *and F. graminearum* (*Fg*)], and taxonomic fungal class. **(B)** Principal Component Analysis (PCA) of substrate utilization profiles. Shapes represent the host plant and colors indicate taxonomic fungal class. Ellipses denote 95% confidence intervals for taxonomic fungal classes. PERMANOVA results indicate a significant effect of taxonomic fungal class (*p* = 0.002) but not of host plant (*p* = 0.572) on substrate utilization profiles.

To investigate the differences in carbon utilization profile related to isolate origin and taxonomy, we first examined the carbon utilization profiles of individual isolates (Figure 2A). The number of carbon sources metabolized by individual isolates ranged from 10 (Supplementary Figure S7A; *Fusarium* sp.; Ta_14) to 90 substrates (Supplementary Figure S7A; *Cladosporium* sp.; Ta_37 and *Penicillium* sp.; Bn_44) out of the 95 substrates tested. On average, isolates used 60 substrates (+/− 20, median = 62), with 49 isolates (52.7%) exceeding this average (Supplementary Figure S7A). This indicates a continuum of carbon use capabilities, ranging from specialists (i.e., able to use only a few specific substrates) to generalists able to utilize nearly all tested carbon sources. On average, each of the 95 substrates tested were consumed by 58 isolates (Supplementary Figure S7B; +/− 21 isolates; median = 63), with large variations across carbon substrates. Glucuronamide was the least frequently used substrate, used by only 6 isolates (Supplementary Figure S7B; 6.45%), while *α*-D-glucose and maltotriose (D-glucose-derived substrate) were utilized by 90 isolates (Supplementary Figure S7B; 96.8%). Notably, no substrate was either universally utilized or completely unused, highlighting the broad and heterogeneous carbon utilization potential of these plant-associated fungal isolates.

The observed carbon utilization profiles were largely determined by the taxonomic affiliation across all taxonomic ranks (PERMANOVA: class, *p* = 0.002, R^2^ = 0.15; order, *p* = 0.003, R^2^ = 0.22; family, *p* = 0.003, R^2^ = 0.31; genus, *p* = 0.021, R^2^ = 0.34; species, *p* = 0.005, R^2^ = 0.58; Supplementary Table S3, Figure 2B). The taxonomy explained up to 58% of carbon utilization at the species level and only 15% at the class level (Supplementary Table S3). Interestingly, the profiles were more contrasted between specific taxa. At the class level, Dothideomycetes differed significantly from Eurotiomycetes (pairwise PERMANOVA, *p* = 0.025) and Mucoromycetes (pairwise PERMANOVA, *p* = 0.025), while Sordariomycetes also diverged from Eurotiomycetes (pairwise PERMANOVA, *p* = 0.025) and Mucoromycetes (pairwise PERMANOVA, *p* = 0.026).

To assess whether these carbon utilization patterns are shaped by the ecological origin of the isolates, we investigated the influence of the host plant species, the plant compartment and the geographic region in France from which the fungi have been isolated. A PERMANOVA analysis on the complete carbon utilization profiles revealed no significant influence of host plant species, plant compartment, or geographic region in France on carbon utilization (PERMANOVA, *p* > 0.05; Supplementary Table S3; Figure 2B).

### 3.4 Specific carbon utilization profile associated with competitive abilities

In order to investigate whether a carbon utilization signature of competitiveness against *Rs* and *Fg* can be detected, we compared carbon utilization profiles of competitive isolates and non-competitive isolates.

**Figure 3:**
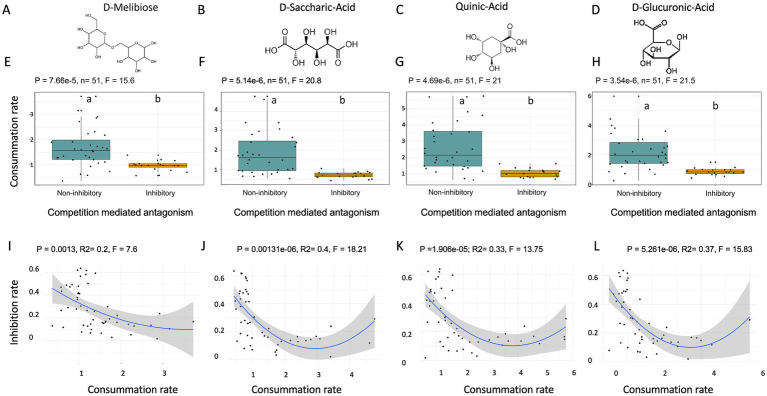
Differentiation of the utilization of four metabolites between competitive isolates and non-competitive isolates against *Rs* and isolated from oilseed rape. **(A–D)** Chemical structures of the 4 analyzed metabolites: D-Melibiose **(A)**, D-Saccharic Acid **(B)**, Quinic Acid **(C)**, and D-Glucuronic Acid **(D)**. **(E–H)** Boxplots showing carbon substrate utilization rates in fungal isolates grouped by competitive isolates and non-competitive isolates against *Rs* for D-Melibiose (**E**; Kruskall-Wallis, *p* = 0.0000766, *F* = 15.6), D-Saccharic Acid (**F**; Kruskall-Wallis, *p* = 0.00000514, *F* = 20.8), Quinic Acid (**G**; Kruskall-Wallis, *p* = 0.0000469, *F* = 21), and D-Glucuronic Acid (**H**; Kruskall-Wallis, *p* = 0.00000354, *F* = 21.5). Significant differences in utilization rates were observed across all metabolites. **(I–L)** Polynomial regression analyses showing a significant inverse relationship between metabolite utilization rate and inhibition rate for D-Melibiose (**I**: *p* = 0.0013, Adjusted R^2^ = 0.2, *F* = 7.6), D-Saccharic Acid (**J**: *p* = 0.00131, Adjusted R^2^ = 0.4, *F* = 18.21), Quinic Acid (**K**: *p* = 1.9e-5, Adjusted R^2^ = 0.33, *F* = 13.75), and D-Glucuronic Acid (**L**: *p* = 5.26e−6, Adjusted R^2^ = 0.37, *F* = 15.83). Regression lines (blue) are shown with 95% confidence intervals (shaded area). These findings suggest that higher carbon substrat utilization is associated with a reduced ability to inhibit *Rs* growth.

Isolates from oilseed rape that had strong competitive abilities against *Rs* had a significantly lower metabolic distance to *Rs* (i.e., using similar carbon substrates) than non-competitive isolates, whether competition-mediated antagonism was considered as a categorical or continuous variable (Kruskal-Wallis; *p* = 0.014; *F* = 6.03; Supplementary Figure S8A; ANOVA; *p* = 0.004; R^2^ = 0.17; Supplementary Figure S8C). Therefore, isolates using the same carbon substrates as *Rs* exhibited stronger *Rs* growth inhibition. Similar results were observed when examining the number of carbon substrates used by competitive isolates versus non-competitive isolates. Specifically, competitive isolates against *Rs* used significantly fewer carbon substrates compared to non-competitive isolates (Kruskal-Wallis; *p* = 0.000322; *F* = 12.9; ANOVA, *p* = 0.0002, R2 = 0.23, *F* = 16.13; Supplementary Figures S9A,C). The observed carbon utilization signature was confirmed using a principal component analysis (PCA, Supplementary Figure S10D) and a PERMANOVA (PERMANOVA, R^2^ = 0.09296, p = 0.003; Supplementary Figure S10D) highlighting significant differences between carbon utilization profile of competitive and non-competitive isolates. To consolidate these results and identify the carbon substrates that may explain competition-mediated antagonism against *Rs*, we used three complementary statistical analyses: a Random Forest (RF), a PPLS-DA and a PCA (Supplementary Figures S10A–C). All three approaches confirmed that carbon utilization profiles can explain the observed competition-mediated antagonism (competitive isolates vs. non-competitive isolates) (model accuracy score RF = 92%, *p* = 4.9e-04; PPLS-DA; 999 permutations; CER = 0.13333, *p* = 0.001, R2 = 89.2; Supplementary Figures S10A–C). These analyses of the carbon utilization profiles of isolates revealed specific carbon substrates that distinguish competitive isolates from non-competitive isolates against *Rs*. Four substrates were identified by all three analyses (RF, PCA, PPLS-DA) as being significantly associated with the level of competition-mediated antagonism: quinic acid (F12), D-saccharic acid (G01), D-melibiose (D04) and D-glucuronic acid (C03) (Figures 3A–D). These four carbon molecules were systematically and significantly less utilized by competitive isolates (Kruskal-Wallis test; *p* < 0.05; Figures 3E–H). Interestingly, the relationship between inhibition rate and substrate utilization was significant but non-linear (polynomial model; *p* < 0.05; Figures 3I–L).

In contrast, for wheat isolates tested against *Fg,* the metabolic distance to *Fg* (Supplementary Figures S8B,D) was similar between competitive and non-competitive isolates as well as the number of carbon substrates consumed (Kruskal-Wallis, *p* > 0.05; ANOVA, *p* > 0.05, Supplementary Figures S9B,D). To validate the fact that competitive and non-competitive isolates wheat isolates do not have a different metabolic profile, we used the same approach as for oilseed rape isolates. No difference in carbon utilization profiles was detected using a principal component analysis (PCA, Supplementary Figure S10E) and a PERMANOVA (PERMANOVA, *p* > 0.05; Supplementary Figure S10E). To consolidate these results, we also performed a Random Forest (RF) and a PPLS-DA analysis. Both approaches confirmed that the carbon utilization profiles did not explain the observed competitive abilities of wheat isolates against *Fg*.

### 3.5 Specific carbon utilization profile associated with antifungal activities

In order to investigate the presence of a carbon utilization signature for antifungal activity on *Rs* and *Fg* we compared the carbon utilization profiles of producer and non-producer isolates.

**Figure 4:**
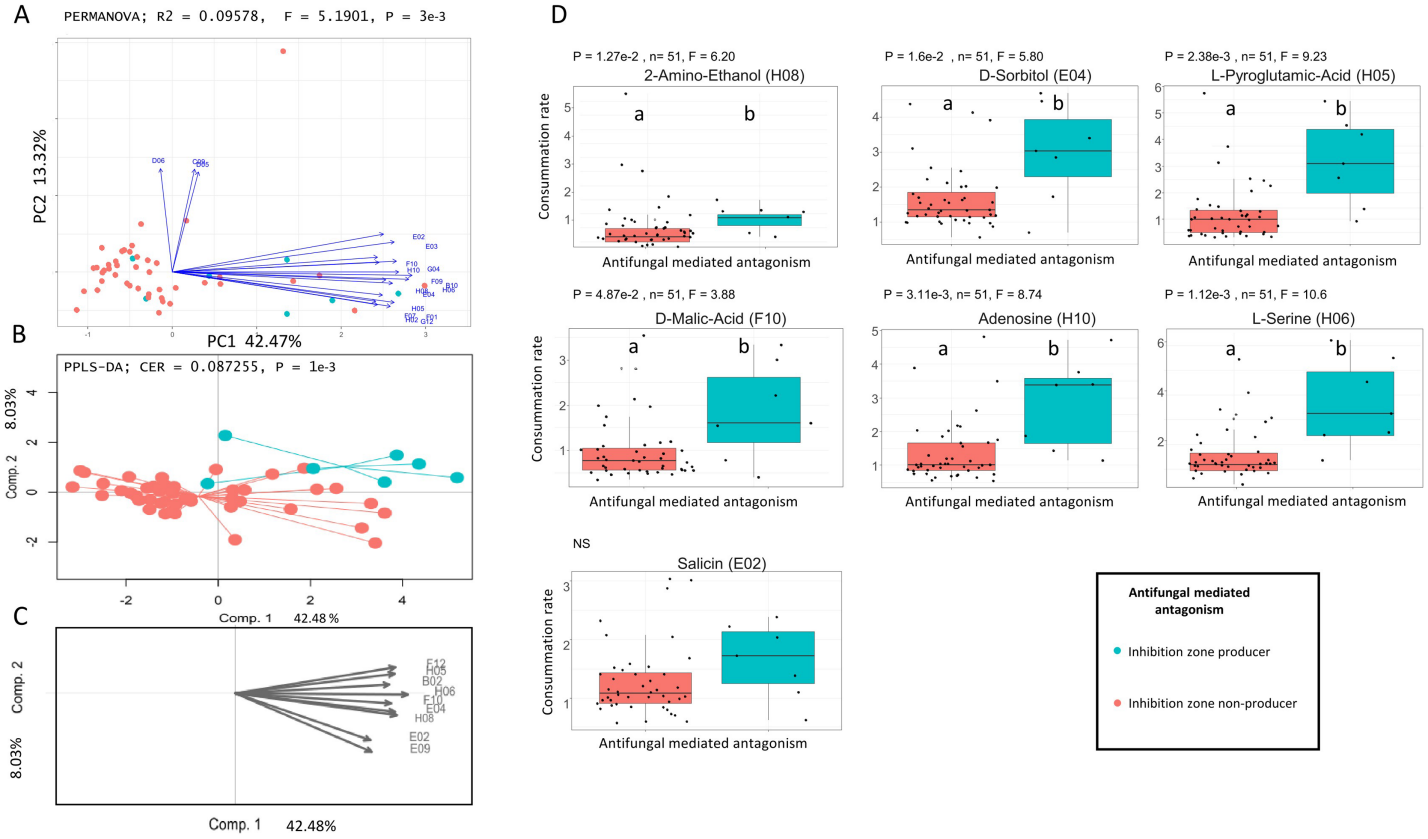
Differentiation of carbon utilization profiles between antifungal producer isolates and non-antifungal producer isolates from oilseed rape and tested against *Rs*. **(A)** Principal Component Analysis (PCA) based on Euclidean dissimilarity of carbon utilization profiles. Antifungal mediated antagonistic isolates are shown in turquoise, while non-antifungal producer isolates are shown in red. Vectors indicate the carbon substrates with the highest contribution to the ordination (threshold ≥ 0.8). PERMANOVA revealed a significant difference between groups (R^2^ = 0.09578, *F* = 5.1901, *p* = 0.003). **(B)** Partial Least Squares Discriminant Analysis (PPLS-DA) showing separation between antifungal producer isolates and non-antifungal producer isolates, with a classification error rate (CER) of 0.087255 and *p* = 0.001. **(C)** Loadings plot from the PPLS-DA identifying the most discriminant carbon substrates (threshold ≥ 0.8). **(D)** Boxplots showing the relative utilization of key discriminant carbon substrates identify by PCA and PPLS-DA: 2-Amino-Ethanol (H08) - *p* = 1.27e-2, *F* = 6.20, D-Sorbitol (E04) - *p* = 1.6e−2, *F* = 5.80, L-Pyroglutamic Acid (H05) - *p* = 2.38e−3, *F* = 9.23, D-Malic Acid (F10) - *p* = 4.87e−2, *F* = 3.88, Adenosine (H10) - *p* = 3.11e-3, *F* = 8.74, L-Serine (H06) - *p* = 1.12e−3, *F* = 10.6, Salicin (E02) - *p* = 0.112, *F* = 2.52 (not significant). Boxplots display individual values, group medians, and interquartile ranges. Letters indicate statistically significant differences based on Kruskal-Wallis (*p* < 0.05).

We used the same approach to investigate the carbon utilization patterns underlying the antifungal-mediated antagonism as previously with the competition-mediated antagonism. In this case, PCA (PERMANOVA; 999 permutations, Df = 1, SumOfSqs = 229.86, R^2^ = 0.09578, *F* = 5.1901, *p* = 0.003; Figure 4A) and PPLS-DA (999 permutations; CER = 0.08, *p* = 0.001, R2 = 67.9) but not RF (*p* > 0.05) revealed a significant difference in the consumption of carbon substrates between the two groups (i.e., inhibition zones producer and non-producer) of isolates derived from oilseed rape and tested against *Rs*. A total of 28 substrates were identified as discriminant by both analytical approaches. Unlike isolates showing a competition-mediated antagonism against *Rs*, those that showed an antifungal-mediated antagonism consistently consumed more substrates than those that did not (Kruskal-Wallis; *p* < 0.05; Figure 4B). This trend was confirmed by the overall number of carbon substrates utilized, with isolates producing inhibition zones using a larger diversity of carbon substrates (Supplementary Figure S11A) (Kruskal-Wallis; *p* = 0.00821; F-statistic = 6.99). However, because only 2 wheat isolates exhibited antifungal activity against *Fg,* the statistical power was too low to investigate the link with carbon utilization profile.

### 3.6 Proteomic markers of antagonism

In order to identify proteomic signatures associated with competitive isolates against our competitors, we used EsMecaTa to obtain enzyme commission numbers (ECs) deduced from reference proteomes available in databases. This strategy allowed us to identify ECs present only in competitive isolates against one of the two target competitors (*Fg* or *Rs*).

Firstly, we found no specific EC shared between all competitive isolates from wheat and tested against *Fg*. Consequently, while ECs associated with competitive abilities were identified, they were present only in a single proteomic model corresponding to one isolate. For wheat isolates reducing *Fg* growth, eight specific enzymatic commission numbers (ECs) were identified (Table 1), all belonging to a single proteomic model *Linnemannia* sp. associated with the isolate Ta_11. Among these, two are related to the Krebs cycle (EC 1.14.11.30 = HIF hydroxylase, EC 1.4.1.21 = aspartate dehydrogenase), suggesting a potential link with energy metabolism (Table 1). Three ECs (EC 3.4.21.7 = plasmin, EC 3.4.24.18 = meprin A; EC 3.4.24.63 = meprin B) are involved in protein and amino acid hydrolysis, while the remaining three (EC 2.4.1.68 = glycoprotein fucosyltransferase, EC 2.4.1.214 = glycoprotein 3-alpha-L-fucosyltransferase; EC 2.7.1.52 = fucokinase) participate in fructose degradation, a carbon source included in our carbon utilization profile (Table 1). However, no significant differences were observed in the utilization of fructose-containing substrates, either D-fructose (Kruskal-Wallis, *p* = 0.423, ANOVA; *p* > 0.05; Supplementary Figures S12B,D) or the D-Psicose/D-Fructose mixture (Kruskal-Wallis, *p* = 0.606, ANOVA, p > 0.05; Supplementary Figures S12B,D) between competitive isolates and non-competitive isolates.

**Table 1:** ECs identified in competitive isolates derived from wheat and tested on *Fg*.

EsMecaTa model	EC number	EC name	Classification	Isolate
Linnemannia	1.14.11.30	HIF hydroxylase	Krebs cycle	Ta_11
Linnemannia	1.4.1.21	Aspartate dehydrogenase	Krebs cycle	Ta_11
Linnemannia	2.4.1.214	Glycoprotein 3-alpha-L-fucosyltransferase	Carbon metabolism	Ta_11
Linnemannia	2.4.1.68	Glycoprotein fucosyltransferase	Carbon metabolism	Ta_11
Linnemannia	2.7.1.52	Fucokinase	Carbon metabolism	Ta_11
Linnemannia	3.4.21.7	Plasmin	Amino acid hydrolysis	Ta_11
Linnemannia	3.4.24.18	Meprin A	Amino acid hydrolysis	Ta_11
Linnemannia	3.4.24.63	Meprin B	Amino acid hydrolysis	Ta_11

Enzyme commission numbers (ECs) identified in the EsMecaTa metabolic model for wheat-derived competitive isolates assigned at genus level and tested against *Fg*. Each enzyme is associated with his EC number, his name, the functional category into which it has been classified (Krebs cycle, carbon metabolism, or amino acid hydrolysis), and the isolate in which it was detected.

Similarly, analysis of oilseed rape isolates revealed a set of exclusive enzymatic commission numbers (ECs) present only in competitive isolates. 14 ECs were present only in the predicted proteomes of isolates with highly competitive abilities against *Rs*. However, no shared ECs were identified between competitive isolates against *Fg* and against *Rs*, indicating distinct biochemical mechanisms depending on the competitor. The ECs identified correspond to three proteomic models (three taxonomic groups). A model corresponding to the genus *Trichoderma*, includes six isolates and reveals the presence of six competition-specific ECs (Table 2). These ECs are grouped into four main functional categories. One EC are involved in carbon metabolism (EC 3.2.1.164 = endo-1,6- beta - galactanase), one EC was linked to a protective mechanism against proteolytic degradation (EC 4.2.1.95 = kievitone hydratase), two ECs were identified in amino acid degradation pathways as well as in lipid and phospholipid metabolism (EC 2.1.1.103 = phosphoethanolamine methyltransferase, EC 2.5.1.26 = alkylglycerone-phosphate synthase), which are critical for maintaining cellular membrane integrity, one EC are unclassified (EC 1.13.12.5 = Renilla-luciferin oxygen2-oxidoreductase) and the last one was associated with secondary metabolism and more precisely in the biosynthetic pathway of penicillin and cephalosporin (EC 6.3.2.26 = ACV synthetase). A second model corresponding to the genus *Botrytis* with a single isolate highlighted a set of seven competition-specific ECs (Table 2). These ECs are grouped into three main functional categories. Two specific ECs were identified in amino acid degradation pathways as well as in lipid and phospholipid metabolism (EC 1.2.1.47 = 4-N-dimethylamino butyraldehyde dehydrogenase, EC 3.4.24.20 = peptidyl lysine metalloproteinase), which are critical for maintaining cellular membrane integrity. Additionally, 3 ECs were associated with secondary metabolism and involved in the biosynthesis of terpene quinones (EC 2.2.1.9 = SEPHCHC synthase, EC 4.2.1.113 = OSB synthase, EC 4.2.99.20 = SHCHC synthase). One EC is involved in carbon metabolism (EC 4.6.1.14 = glycosylphosphatidylinositol diacylglycerol-lyase), including inositol metabolism, which was also tested in Biolog FF microplates. The inositol metabolic pathway, enriched in competitive isolates against *Rs*, was significantly less consumed by competitive isolates compared to non-competitive isolates (Kruskal-Wallis test, *p* = 0.00005, ANOVA; *p* = 2.18e-11, *F* = 8.621, Supplementary Figures S12A,C). The last EC is unclassified (EC 4.4.1.32 = cpcE). Finally, the third model is associated with the family Mucoraceae, and comprises a single isolate and a single EC (Table 2). This EC is associated with secondary metabolism and especially the catalyses of cytokinins (EC 1.5.99.12 = cytokinin dehydrogenase).

**Table 2:** ECs identified in competitive isolates derived from oilseed rape and tested on *Rs*.

EsMecaTa model	EC number	EC name	Classification	Isolates
Trichoderma	1.13.12.5	Renilla-luciferin: oxygen2-oxidoreductase	Unclassified	Bn_01, Bn_03, Bn_05, Bn_10, Bn_30, Bn_04
Trichoderma	2.1.1.103	Phospho ethanolamine methyltransferase	Amino acid and lipid metabolism	Bn_01, Bn_03, Bn_05, Bn_10, Bn_30, Bn_04
Trichoderma	2.5.1.26	Alkylglycerone-phosphate synthase	Amino acid and lipid metabolism	Bn_01, Bn_03, Bn_05, Bn_10, Bn_30, Bn_04
Trichoderma	3.2.1.164	endo-1,6-beta-galactanase	Carbon metabolism	Bn_01, Bn_03, Bn_05, Bn_10, Bn_30, Bn_04
Trichoderma	4.2.1.95	Kievitone hydratase	Proteolitic degradation protection	Bn_01, Bn_03, Bn_05, Bn_10, Bn_30, Bn_04
Trichoderma	6.3.2.26	ACV synthetase	Secondary metabolism	Bn_01, Bn_03, Bn_05, Bn_10, Bn_30, Bn_04
Botrytis	1.2.1.47	4-N-trimethylamino butyraldehyde dehydrogenase	Amino acid and lipid metabolism	Bn_31
Botrytis	2.2.1.9	SEPHCHC synthase	Secondary metabolism	Bn_31
Botrytis	3.4.24.20	Peptidyl lysine metalloproteinase	Amino acid and lipid metabolism	Bn_31
Botrytis	4.2.1.113	OSB synthase	Secondary metabolism	Bn_31
Botrytis	4.2.99.20	SHCHC synthase	Secondary metabolism	Bn_31
Botrytis	4.4.1.32	cpcE	Unclassified	Bn_31
Botrytis	4.6.1.14	Glycosylphosphatidylinositol diacylglycerol-lyase	Carbon metabolism	Bn_31
Mucoraceae	1.5.99.12	Cytokinin dehydrogenase	Secondary metabolism	Bn_06

Enzyme commission numbers (ECs) identified in the EsMecaTa metabolic model for oilseed rape-derived competitive isolates assigned at genus level and tested against *Rs*. Each enzyme is associated with its EC number, its name, the functional category into which it has been classified (Unclassified, Amino acid and lipid metabolism, Carbon metabolism, Proteolytic degradation protection or Secondary metabolism), and the isolate in which it was detected.

### 3.7 Antagonistic potential of fungal isolates across competitors

**Figure 5:**
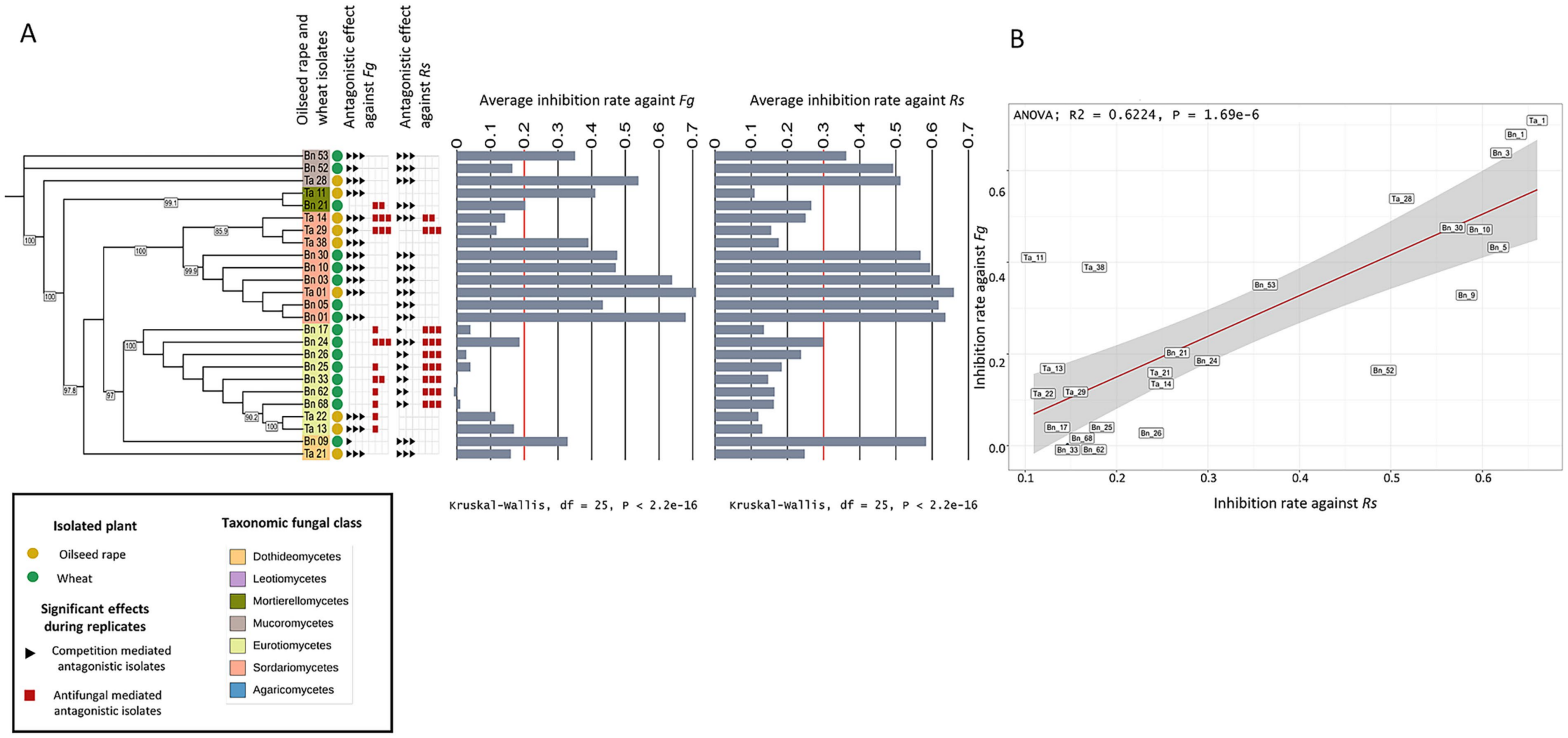
Antagonistic potential of fungal isolates against two competitors. **(A)** Maximum likelihood tree of the fungal isolates based on SSU sequences, annotated with the plant host species (oilseed rape in green and wheat in yellow), fungal taxonomic class (colored squares), and red and black squares next to each isolate denote significant competition-mediated antagonism and antifungal-mediated antagonism, respectively in a biological replicate. Average inhibition rates against *Fg* and *Rs* are shown as bar plots with a red line marking the limit between competitive isolates and non-competitive isolates. A Kruskal-Wallis test indicates significant differences among isolates for both competitors (*Fg*: χ^2^ = 229.9, df = 25, *p* < 2.2e−16; *Rs*: χ^2^ = 215.59, df = 25, *p* < 2.2e-16). **(B)** Correlation between inhibition rates against *Fg* and *Rs*, with a linear regression line (R^2^ = 0.6224, *p* = 1.69e-6) and 95% confidence interval (gray shading).

In order to test whether competition-mediated antagonism and/or antifungal-mediated antagonism are competitor-specific, we tested all antagonistic isolates on both competitors in dual confrontation tests. Of the 25 isolates tested on both competitors (16 isolated from oilseed rape and 9 isolated from wheat), 9 isolates significantly reduced the growth of both competitors in all three replicates of the experiment (Kruskal Wallis; *p* < 0.05; Figure 5A). These isolates belonged to the Mucoromycetes, Sordariomycetes and Dothideomycetes classes and had a similar average inhibition rate in the presence of both competitors (0.463 when interacting with *Fg* and 0.49 when interacting with *Rs*). Therefore, both competitors are similarly affected by the same fungal groups. Interestingly, the ability of isolates to reduce growth of both competitors was highly correlated (ANOVA; *p* = 1.69e-6; F-statistic: 40.55; R2: 0.62; Figure 5B) suggesting that the ability to reduce growth of a single competitor explains 68% of the ability to reduce growth of another competitor. Regarding antifungal-mediated antagonism, among the nine isolates previously identified, only two were able to do antifungal-mediated antagonism repeatedly on the two competitors (Figure 5A). One isolate was isolated from oilseed rape and one from wheat and belong to the Eurotiomycetes (Bn_24) and Sordariomycetes (Ta_29), respectively.

## 4 Discussion

### 4.1 Broad diversity in carbon utilization of fungal isolates but limited ecological structuring

In any given environment, the metabolism of fungi is shaped by inherited metabolic capacities and by context-dependent abiotic and biotic constraints (McGuire et al., 2010). From this eco-evolutionary perspective, this potential reflects selective pressures that acted on the fungus lineages over time. Consequently, one might expect that fungi inhabiting distinct ecological niches would exhibit divergent carbon utilization signatures, indicative of local adaptations and environmental filtering (McGuire et al., 2010; Saunders et al., 2010; Talbot et al., 2014). In plant-associated microbiota, these carbon utilization traits are influenced by both plant-dependent factors (e.g., root exudates) and plant-independent variables such as soil physicochemical properties. While many studies have successfully described the variations in mycobiota composition across diverse ecological contexts (e.g., climatic gradients, host plant species; Thiergart et al., 2020), including databases and meta-analyses (Větrovský et al., 2019; Větrovský et al., 2020), much less is known about the carbon utilization capacity of individual fungi within natural fungal communities.

Leveraging a large collection of fungal isolates from two host plants, two root-associated compartments and three geographical regions in France, we investigated whether the ecological context influences fungal carbon utilization capacities. Our results reveal a wide spectrum of carbon utilization profiles among isolates. The diversity of carbon used by an isolate can be considered as a proxy of niche width (LeBlanc et al., 2017) and our results reveal a range of niche width highly generalist carbon usage (i.e., isolates capable of metabolizing almost all tested carbon sources; e.g. *Cladosporium* sp. Ta_37 and *Penicillium* sp. Bn_44 with 90 carbon substrates used; Supplementary Figure S7) to highly specialized carbon usage (e.g., *Fusarium* sp. Ta_14 with 10 substrates used; Supplementary Figure S7). However, despite this diversity we found no clear structuring of carbon utilization profiles based on ecological origin. None of the host plant species, the compartment or the geographical origin significantly structured isolates’ carbon utilization profile (Figure 1; Supplementary Table S3). Instead, carbon usage was more strongly associated with fungal taxonomy, suggesting that in our experimental system the evolutionary lineage better predicts carbon substrate preferences than local environmental conditions (Figure 2; Supplementary Table S3). Similar results have been obtained by comparing smaller sets of fungal endophytes from semi-arid regions (Knapp and Kovács, 2016) and oomycetes (Masigol et al., 2023) highlighting species specific carbon utilization profiles. Interestingly, while we did not detect host-specific patterns, LeBlanc et al., (2017) did detect host-specific carbon utilization profiles by focusing on 84 Fusarium species. It is important to note that only two plant species were tested in the present study, potentially explaining this difference with previous studies, and calling for larger host plant screenings. Taken together this suggests that patterns of niche width differentiation through metabolic specialization can be detected at both precise and large taxonomic scales.

Consistent with previous reports highlighting the high metabolic plasticity of soil fungi (Camenzind et al., 2020) this finding suggests carbon utilization potential may not be tightly constrained by ecological settings. In our dataset, more than half of the isolates utilized over 60 substrates, (52.7% above the median of 62, Supplementary Figure S7) suggesting widespread generalism. Several studies have also shown that endophytic fungi, notably those belonging to the genera *Fusarium*, *Alternaria* or *Cladosporium*, can modify their carbon utilization profile according to the nutritional environment or the identity of the host plant (Schulz and Boyle, 2005). Similarly, many soil-associated fungal species, often classified as opportunistic saprotrophs or facultative endophytes, display a wide ecological amplitude and a metabolic repertoire enabling them to exploit different carbon niches (Rodriguez et al., 2009; Porras-Alfaro and Bayman, 2011). These observations reinforce the idea that many plant-associated fungi are not strictly specialized, but rather metabolic generalists adaptable to the fluctuating conditions of root ecosystems, supporting the idea that many root-associated fungi are generalists and are capable of colonizing multiple plant species. Within the root environment, fungi are exposed to a heterogeneous mix of carbon compounds, requiring them to prioritize the most effectively assimilable source substrates. Fungal carbon catabolite repression (CCR) systems allow such prioritization by enabling the preferential use of more energetically favorable compounds (Adnan et al., 2018). CCR is a regulatory mechanism in fungi that allows preferential utilization of easily metabolizable carbon sources, such as glucose, over less favorable ones (Adnan et al., 2018). CCR plays a crucial role in fungal metabolic plasticity and stress responses, which are essential for disease progression in pathogenic fungi. These mechanisms likely represent an evolutionary trade-off between specialization and generalization, shaped by fluctuating resource availability in the rhizosphere (Philippot et al., 2013). Considering that the rhizosphere of plants is considered to be one of the most dynamic interfaces on Earth (Philippot et al., 2013) it is likely that temporal variation in carbon availability also plays a key role in shaping fungal metabolic strategies. While our current profiling has identified differences in carbon substrate utilization according to fungal isolate lineage, uncovering host-mediated constraints on carbon metabolism may require more complex assays with dynamic and diverse carbon sources availability. Incorporating a broader diversity of host plants and fungal taxa would also likely provide opportunities to identify species-specific host-driven carbon utilization.

### 4.2 Carbon substrate utilization shapes antagonistic interactions between fungi

While the carbon utilization of individual fungal isolates provides insights into their ecological strategy and evolutionary history, fungi in natural environments rarely act in isolation. They constantly engage in competitive interactions, particularly for limiting resources, questioning the influence of carbon utilization traits for fungal-fungal interactions. Antagonistic interactions can involve a spectrum of strategies, from rapid resource exploitation and/or niche occupation to the production of antifungal compounds that inhibit the growth of competitors (Mousa and Raizada, 2013; Lugtenberg et al., 2016; Sasse et al., 2018; Hu et al., 2020). Our results demonstrate that many root-associated fungi from wheat and oilseed rape exhibit antagonistic activities against fungal competitors primarily through general competition-mediated antagonism and in some cases specifically by antifungal-mediated antagonism (Figure 1). The observed phenotypes were maintained after 28 days of confrontation, highlighting the importance of the competitive interactions for fungal establishment and survival. Our results indicate that the antagonistic potential of fungal isolates follows phylogenetic patterns for oilseed rape-associated isolates but not wheat-associated isolates, suggesting that fungal taxa have indeed developed contrasted strategies to compete with other fungi. The observed phylogenetic patterns may reflect potential conservation of metabolic pathways within fungal lineages. Previous studies have shown that trait like enzyme profiles and carbon utilization strategies often exhibit phylogenetic signal among fungi (Hedges et al., 2004). Similarly, some biosynthetic gene clusters (BGCs), particularly those involved in the production of secondary metabolites such as antifungals, can be phylogenetically conserved in certain clades (Wisecaver et al., 2014; Spatafora et al., 2017). Further comparative genomics would be needed to determine whether the phylogenetic pattern of fungal-fungal interaction traits reflects conserved functional capacities or ecological convergence. In our system the competitive abilities of isolates were negatively correlated to their metabolic distance to the competitor (Supplementary Figure S8), demonstrating that metabolic proximity, and thus niche overlap, determine competitive interactions between fungi. Interestingly, antagonism against *Rs also* correlated with the use of fewer carbon substrates (Supplementary Figures S9A,C), suggesting a reduction in niche width for competitive isolates compared to non-competitive isolates. This negative correlation between niche width and competitive ability suggests that there might be a competitive cost for using a large number of carbon substrates (i.e., generalist carbon source utilization). This supports the idea that generalist strategies in carbon utilization, while advantageous in fluctuating environments, may be less favorable in more stable conditions.

In contrast, and while we did not validate that antifungal compounds were produced, isolates producing inhibition zones consumed a higher number of carbon substrates than other fungi (Supplementary Figure S11) indicating that diverse carbon substrates may be required to produce molecules responsible for this inhibition. Alternatively, the production of inhibitive compounds might be associated with generalist strategies. Previous work showed that glucose concentration plays a crucial role in the biosynthesis of antifungal volatile organic compounds by *Aureobasidium pullulans* (Yalage Don et al., 2021). While our experimental design did not allow to detect the presence of antifungal volatile organic compounds, it could be hypothesized that carbon metabolism is involved in the biosynthesis of various antifungal molecules, including volatile molecules. Collectively, our results suggest that carbon sources and concentrations may significantly impact the production of antifungal compounds in fungi, and that antifungal-mediated competition shapes fungal metabolic profiles.

Overall, our results indicate that carbon utilization interacts with both the competition-mediated and antifungal-mediated antagonism. Surprisingly we found no link between antagonistic abilities and the carbon utilization profiles for wheat isolates. Strikingly however, we found that the antagonistic abilities of fungi were highly correlated between the two competitors tested (i.e., *Rs* or *Fg*; Figure 5B) demonstrating that a large fraction of the variations observed between isolates are due to competitor-independent mechanisms. Consequently, the observed absence of metabolism-driven competitive abilities in wheat isolates could reflect host-dependent mechanisms. Alternatively, because fewer isolates were tested from wheat, this could reflect a lack of statistical power to identify meaningful carbon utilization patterns, calling for the investigation of a larger fungal isolate pool. In contrast, antifungal-mediated antagonism was competitor-specific (i.e., *Rs* or *Fg*; Figure 5), thus pointing to an inducible mechanism activated in response to a molecular detection and recognition of competitors. Alternatively, this specificity could result from the intrinsic tolerance or resistance of competitor fungi to antifungal molecules. Interestingly, our data show that isolates capable of antifungal-mediated antagonism are generally not the most competitive. This contrast suggests metabolic specialization from a trade-off between the production of antifungal metabolites and the rapid exploitation of environmental resources.

### 4.3 Carbon substrate utilization traits as a functional signature of fungal-fungal antagonism

Herein, we combined two complementary approaches to determine whether specific metabolic patterns could explain fungal-fungal interactions. We analyzed both individual fungal isolates’ carbon utilization potential through Biolog profiling and estimated metabolic capabilities based on taxonomy by mining databases for reference proteomes. Our results indicate that specific substrates are associated with the observed antagonistic abilities of our isolates against fungal competitors. We identified four substrates associated with competition-mediated antagonism against *Rs*: D-melibiose, saccharic acid, quinic acid and D-glucuronic acid (Figure 3). Surprisingly, the relation between the utilization of these substrates and competitive abilities was non-linear (Figure 3). Isolates with high utilization of these carbon sources had consistently low antagonistic potential while those that efficiently metabolized the 4 carbon sources were more prone to display high antagonistic abilities. Because the utilization of these sources is reduced in highly competitive isolates, it is unlikely that their metabolism directly impacts competition but rather that it is connected to important metabolic pathways determining competition. In addition, competitive isolates tended to metabolize a lower diversity of carbon sources (i.e., narrow niche width), suggesting that high competitive abilities are connected to specialized carbon usage. Conversely, isolates displaying inhibition zones used specific carbon sources in greater quantities (Figure 4) and an overall larger diversity of carbon sources, suggesting that the metabolism of specific carbon sources is required for the biosynthesis of the metabolites responsible for the antifungal activity (Yalage Don et al., 2021).

Analysis of predicted proteomes from oilseed rape isolates identified taxon specific enzymatic functions present uniquely in antagonistic isolates. Specifically, these enzymatic functions are involved in lipid metabolism, amino acid metabolism, secondary metabolite biosynthesis and carbon metabolism (Table 2). Consistent with the differential usage of carbon substrates, antagonist isolates of the genus *Linnemannia* are predicted to produce enzymes carbon metabolism and amino acid hydrolysis, supporting the idea that these isolates mobilize central metabolic functions to support their antagonistic activity (Table 1). Taken together, the observation that metabolic distance to the competitor is correlated with antagonistic abilities and that antagonist-specific carbon utilization profiles and predicted enzymatic reactions can be identified, suggest that antagonistic potential could be predictable. While carbon utilization does not fully predict antagonistic abilities of individual fungi, they may be considered as markers to evaluate both competition-mediated and antifungal-mediated antagonistic potential. A number of previous studies have previously screened fungal isolates to identify fungal derived antifungal molecules (Tanney et al., 2016; Xu et al., 2016; Macías-Rubalcava and Sánchez-Fernández, 2017; Deshmukh et al., 2018). However, screening collections of individual fungal isolates is often time consuming and is generally limited to a specific target pathogen, making it difficult to scale to large collections. Our work provides evidence for a set of predictive characteristics to identify highly competitive and inhibitive isolates based on: (i) carbon utilization profile including niche width and metabolic distance to the competitor, (ii) specific carbon substrate utilization and (iii) predicted enzymatic reactions.

Aside from predicting antagonistic potential, our results highlight the role of carbon usage strategies in fungal-fungal interactions and highlight that competition based antagonistic interactions are frequent between plant-associated fungi. While significant efforts have been done to take into account the role of microbe-microbe interactions in microbiome assembly (Hunter et al., 2010; Bakker et al., 2014) and plant holobiont functioning (Mendes et al., 2011), these efforts have been focused on bacterial-bacterial (Cabrefiga et al., 2007) and bacterial-fungal interactions (Getzke et al., 2019). In the meantime, fungal-fungal interactions have received little attention and the results obtained herein call for considering competitive fungal-fungal interactions in the context of the plant holobiont to decipher the complexity of the plant-microbiome assembly. More specifically, profiling carbon utilization profiles of root-associated fungi offers insights into broader ecological strategies and may reveal the hidden role of nutrient usage strategies in structuring complex micro-microbe interaction networks.

## Data Availability

All the data used are available as supplementary data. The fungal collection data and metadata (including ITS and SSU sequences) are publicly available in recherche.data.gouv.fr in the following repositery: https://doi.org/10.57745/ABASFM.
