# Supplementary material for: Carbon substrates utilization determine antagonistic fungal-fungal interactions among root-associated fungi
Source: Front Microbiol. 2025 Aug 14;16:1645107. doi: 10.3389/fmicb.2025.1645107 (PMC12391134; doi:10.3389/fmicb.2025.1645107)
Supplement: Supplementary file 1 [file Data_Sheet_1.docx]

Supplementary Material

# Supplementary Figures


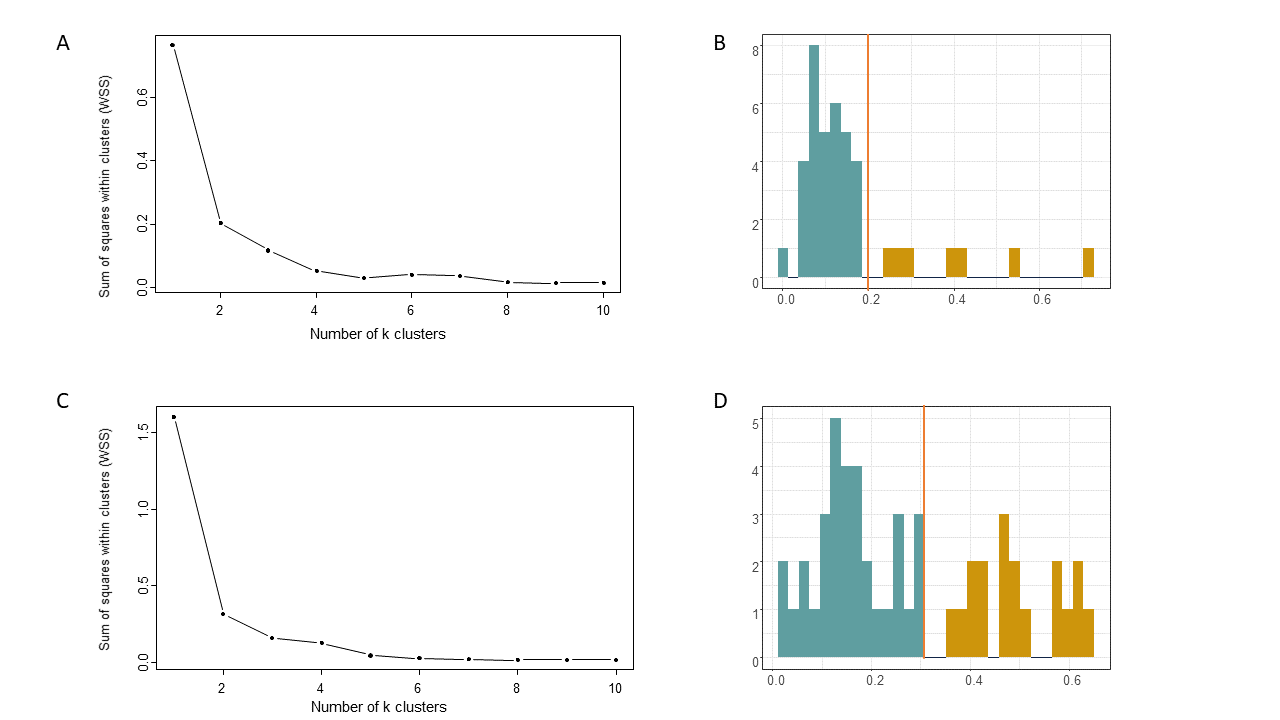


**Supplementary Figure 1.** **Distribution of inhibition rates of fungal isolates against *Fg* and *Rs*.** Sum of squares within clusters (WSS) given different number of k clusters based on kmeans clustering of inhibition rates for fungal isolates tested against *Fg* (A) and *Rs* (C). Histograms showing the frequency distribution of inhibition rates for fungal isolates tested against *Fg* (B) and *Rs* (D). The red vertical line indicates the cutoff value above which isolates are considered as inhibitory (yellow color) and under which isolates are considered as non-inhibitory isolates (blue color).


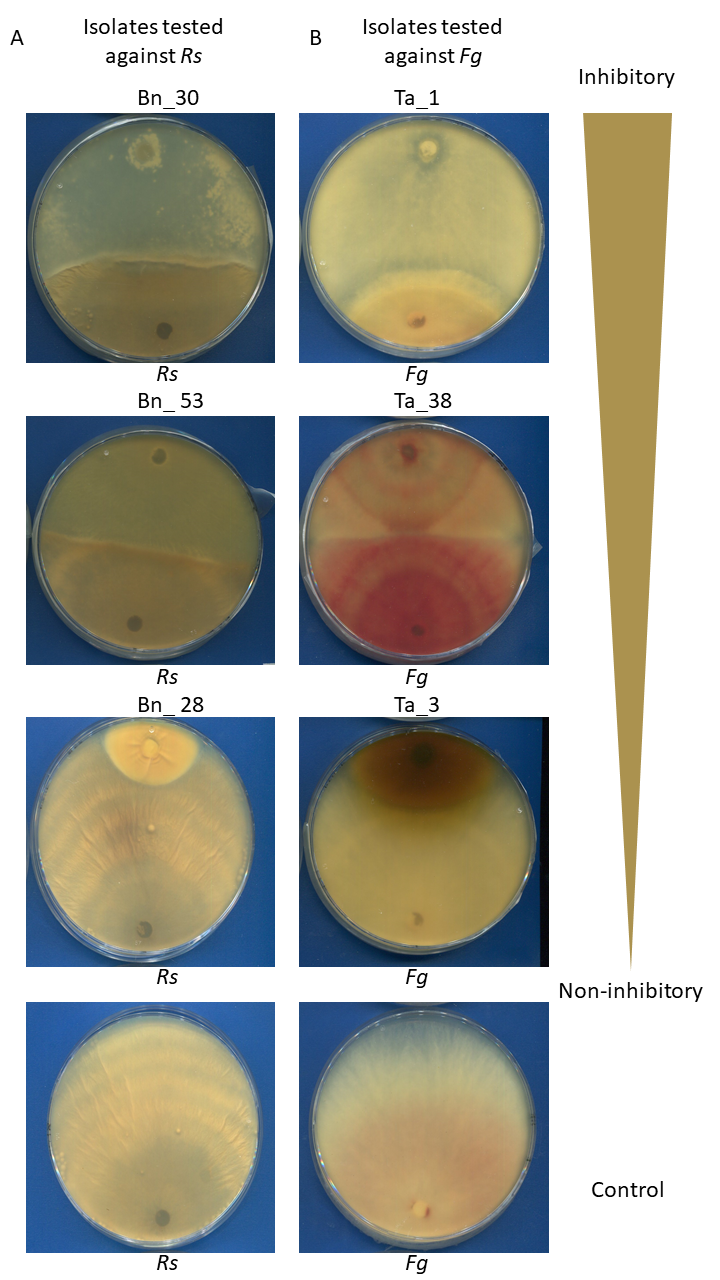


**Supplementary Figure 2. Examples of the range of phenotypes obtained in dual-competition assays.**


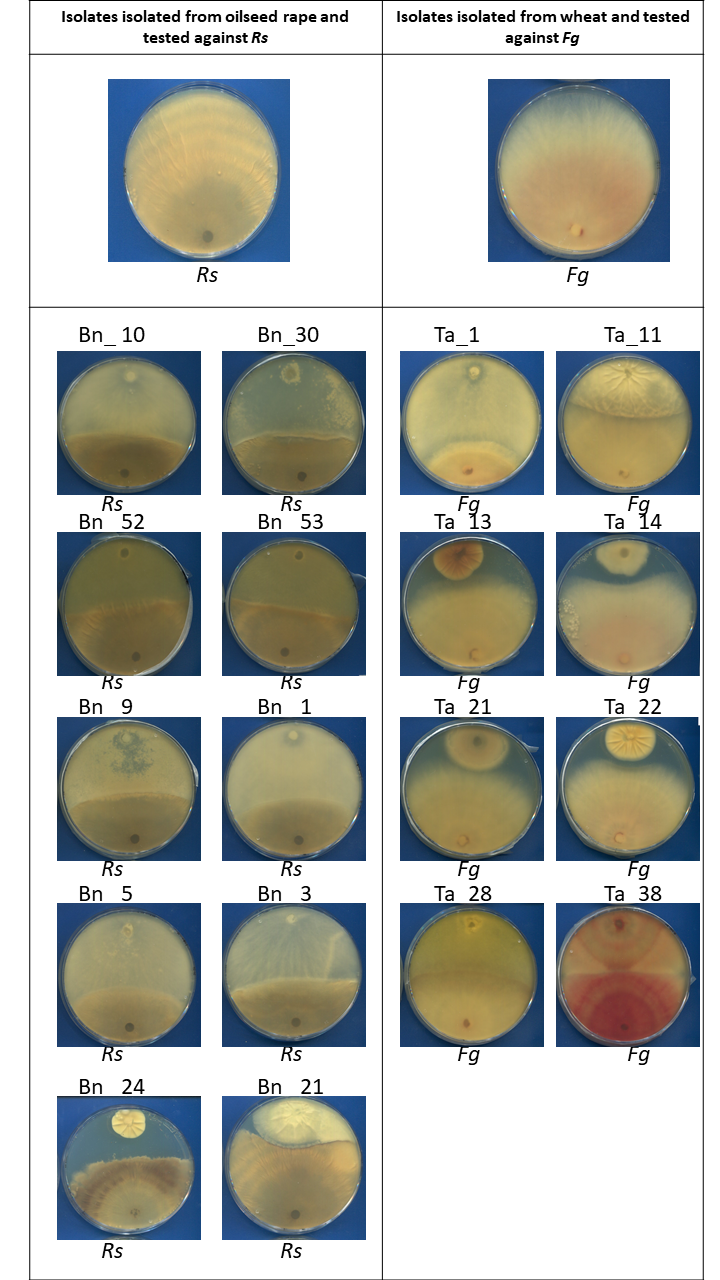


**Supplementary Figure 3.** **Examples of dual-competition assays results for isolates exhibiting competition-mediated antagonism against *Rs* or *Fg*.** A single representative picture out of the 9 independent replicates is shown for each interaction. The pictures on the right show isolates from wheat tested against *Fg*, and the pictures on the left show isolates from rapeseed tested against *Rs*.


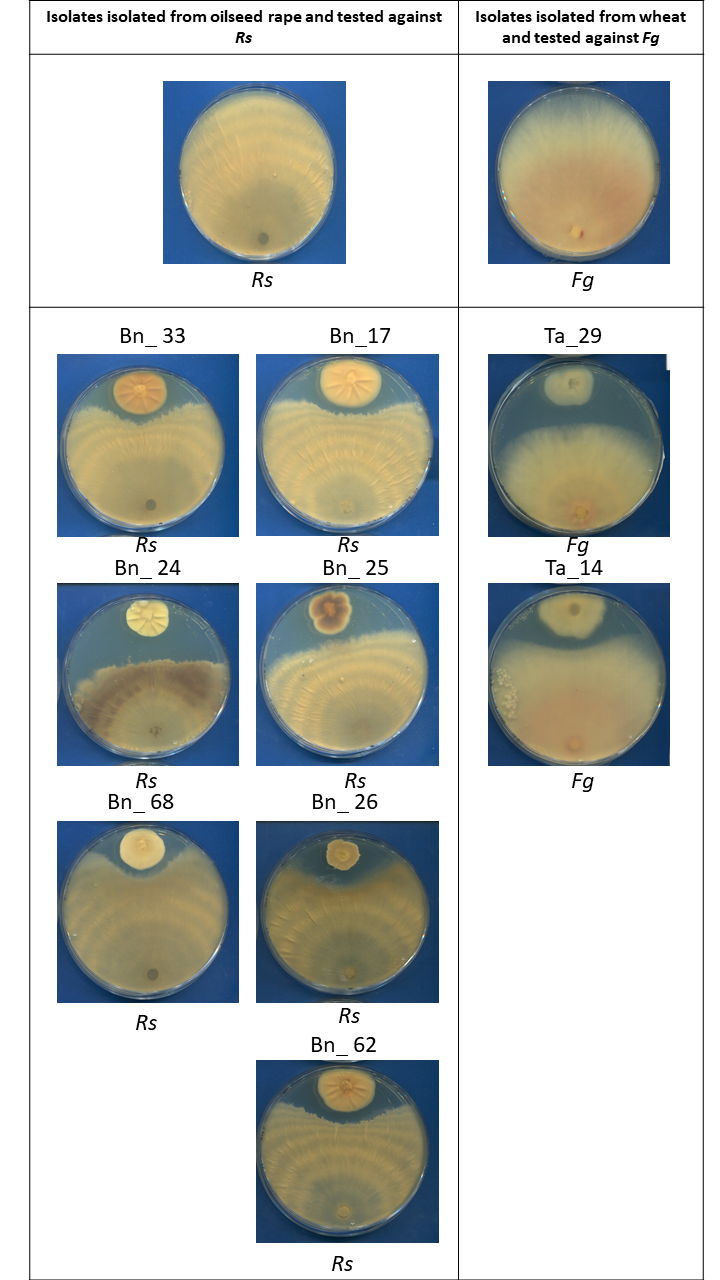


**Supplementary Figure 4.** **Examples of dual-competition assays results dual-competition assays of isolates identified as forming inhibition zones and therefore exhibiting antifungal-mediated antagonism on *Rs* and *Fg*.** A single representative picture out of the 9 independent replicates is shown for each interaction. The pictures on the right show isolates from wheat tested against *Fg*, and the images on the left show isolates from rapeseed tested against *Rs*.


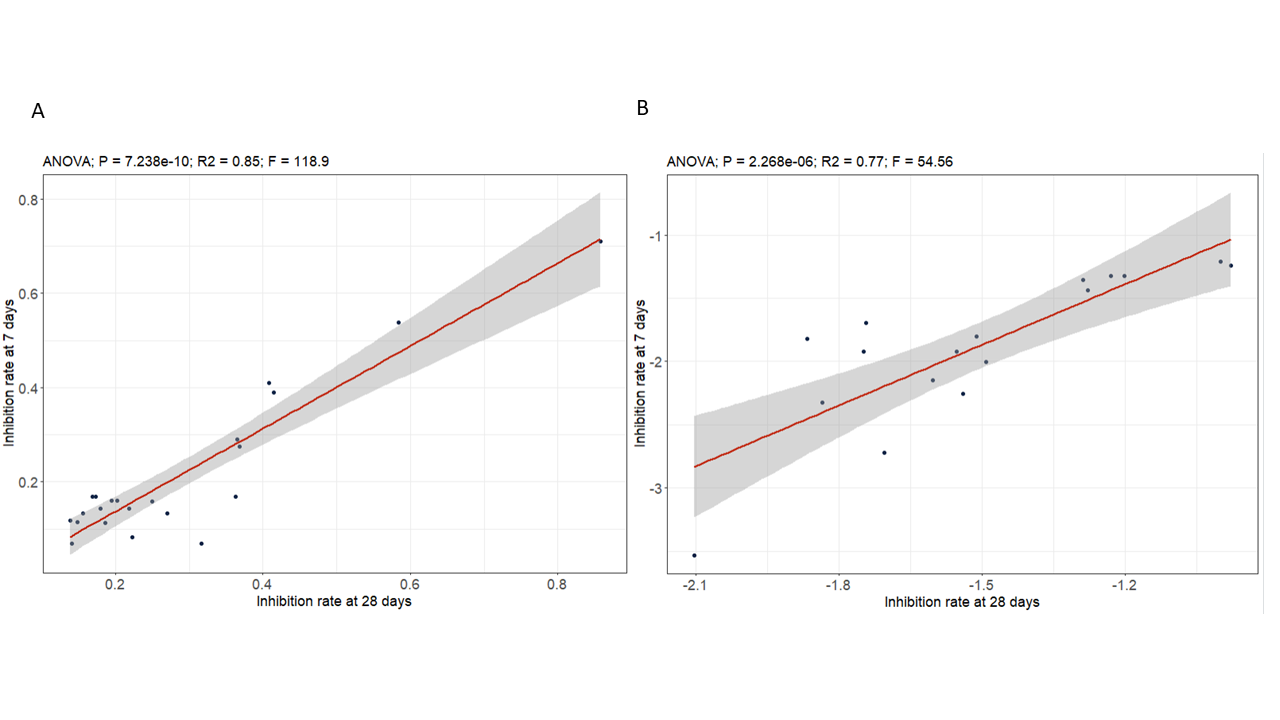


**Supplementary Figure 5.** **Link between inhibition rates at 7 days and 28 days after inoculation.** Correlation between inhibition rates at 7 days and 28 days for (A) isolates tested against *Fg* and isolated from wheat (ANOVA; R² = 0.85, P = 7.23e-10, F = 118.9) and (B) isolates tested against *Rs* and isolated from oilseed rape (ANOVA; R² = 0.77, P = 2.27e-06, F = 54.56).


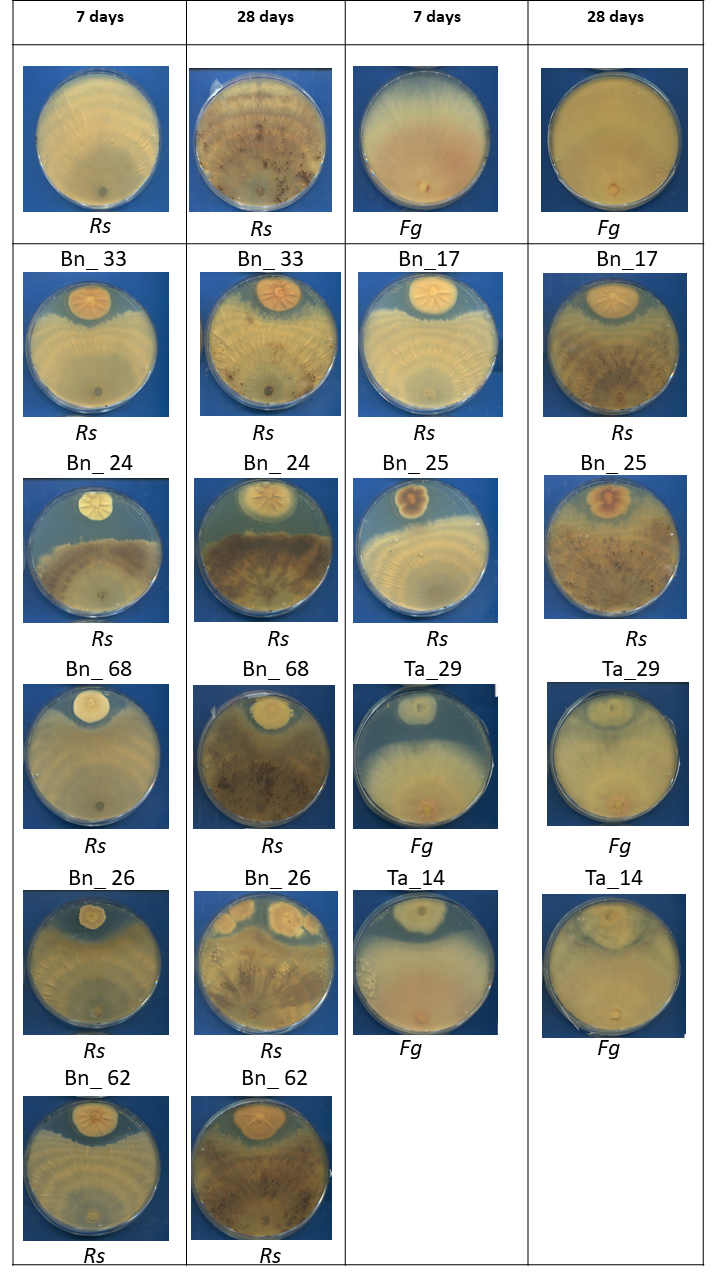


**Supplementary Figure 6. Pictures of Petri dishes showing dual-competition assays of isolates identified as forming inhibition zones and therefore able of antifungal-mediated antagonism on *Rs* and *Fg* at 7 days and 28 days after inoculation.**


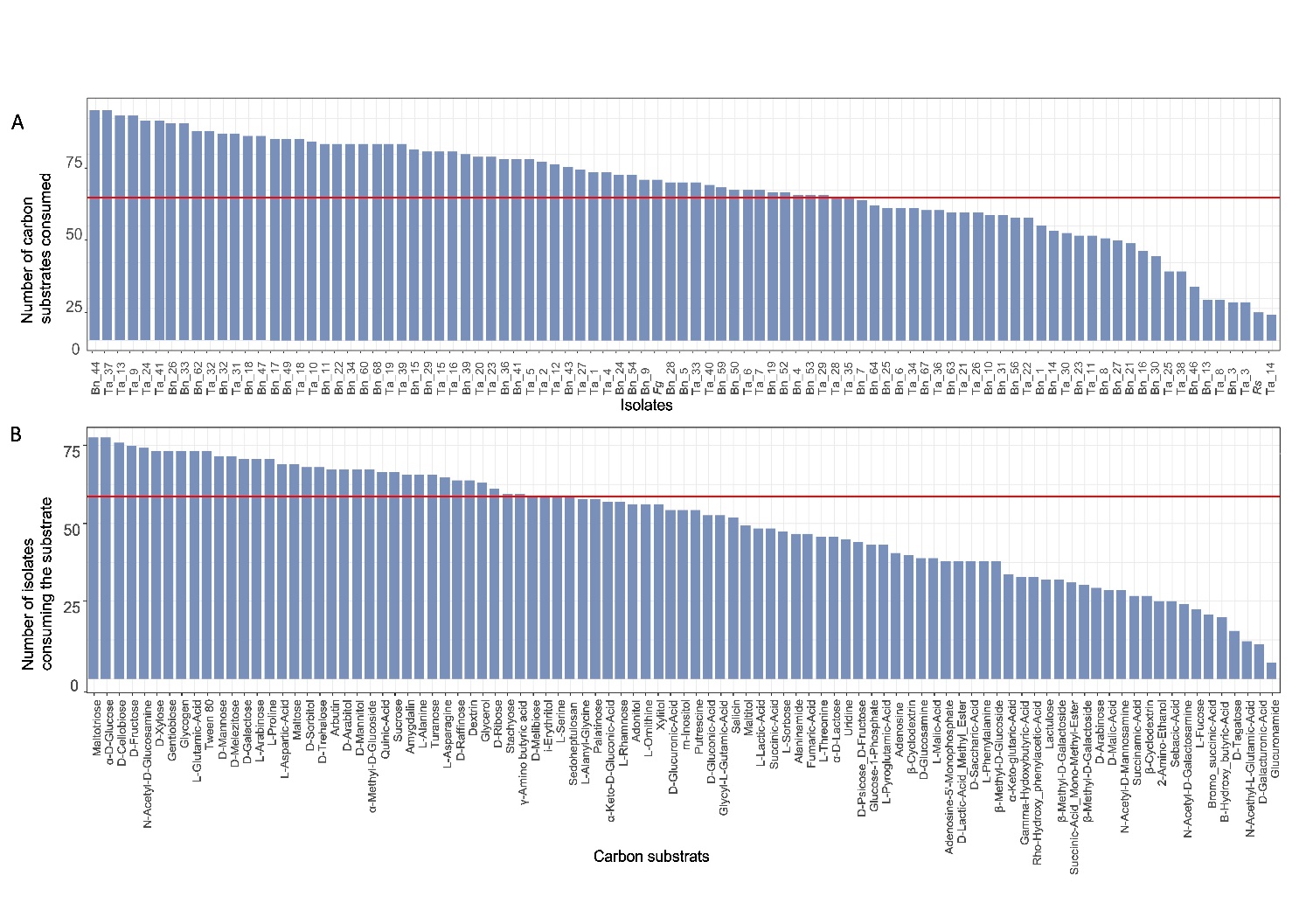


**Supplementary Figure 7.** **Variability in carbon substrate utilization across fungal isolates**. (A) Number of carbon substrates utilized by each fungal isolate. Each bar represents one isolate. The red line indicates the mean number of substrates consumed across all isolates (mean = 56). (B) Number of fungal isolates that utilized each carbon substrate. Each bar represents one substrate. The red line indicates the mean number of isolates able to consume the substrates (mean = 56).


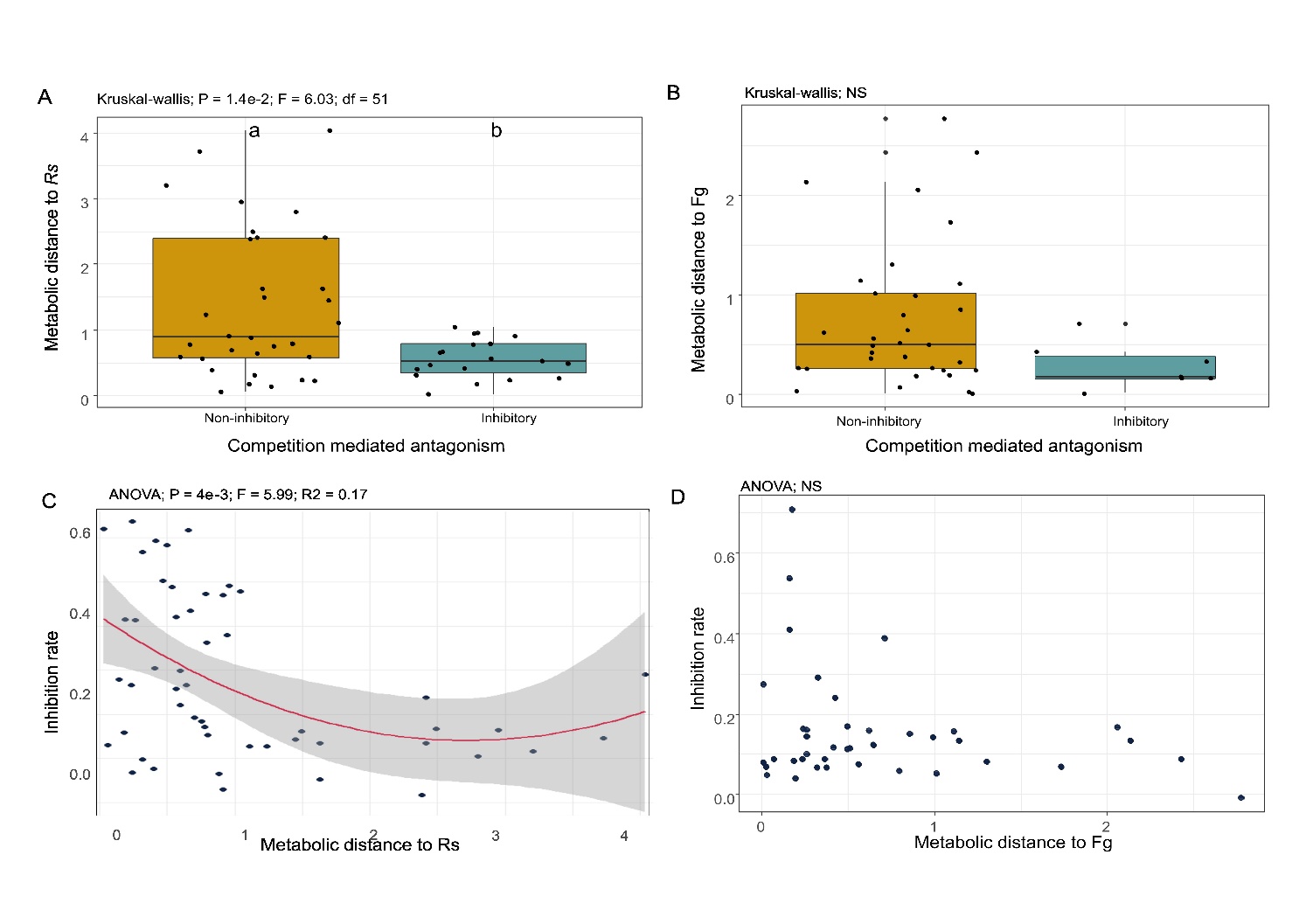


**Supplementary Figure 8.** **Relationship between metabolic distance and inhibition rate.** (A) Metabolic distance to *Rs* according to inhibition rate (classified as "inhibitory" or "non-inhibitory"). A Kruskal-Wallis test reveals a significant difference in metabolic distance between inhibition rate groups (p = 0.014; F = 6.03; df = 51). (B) Metabolic distance to *Fg* according to inhibition rate. (C) Linear regression showing a significant negative relationship between metabolic distance to *Rs* and inhibition rate (ANOVA, p = 0.004389; R² = 0.1368). (D) Scatter plot of inhibition rate as a function of metabolic distance to *Fg*.


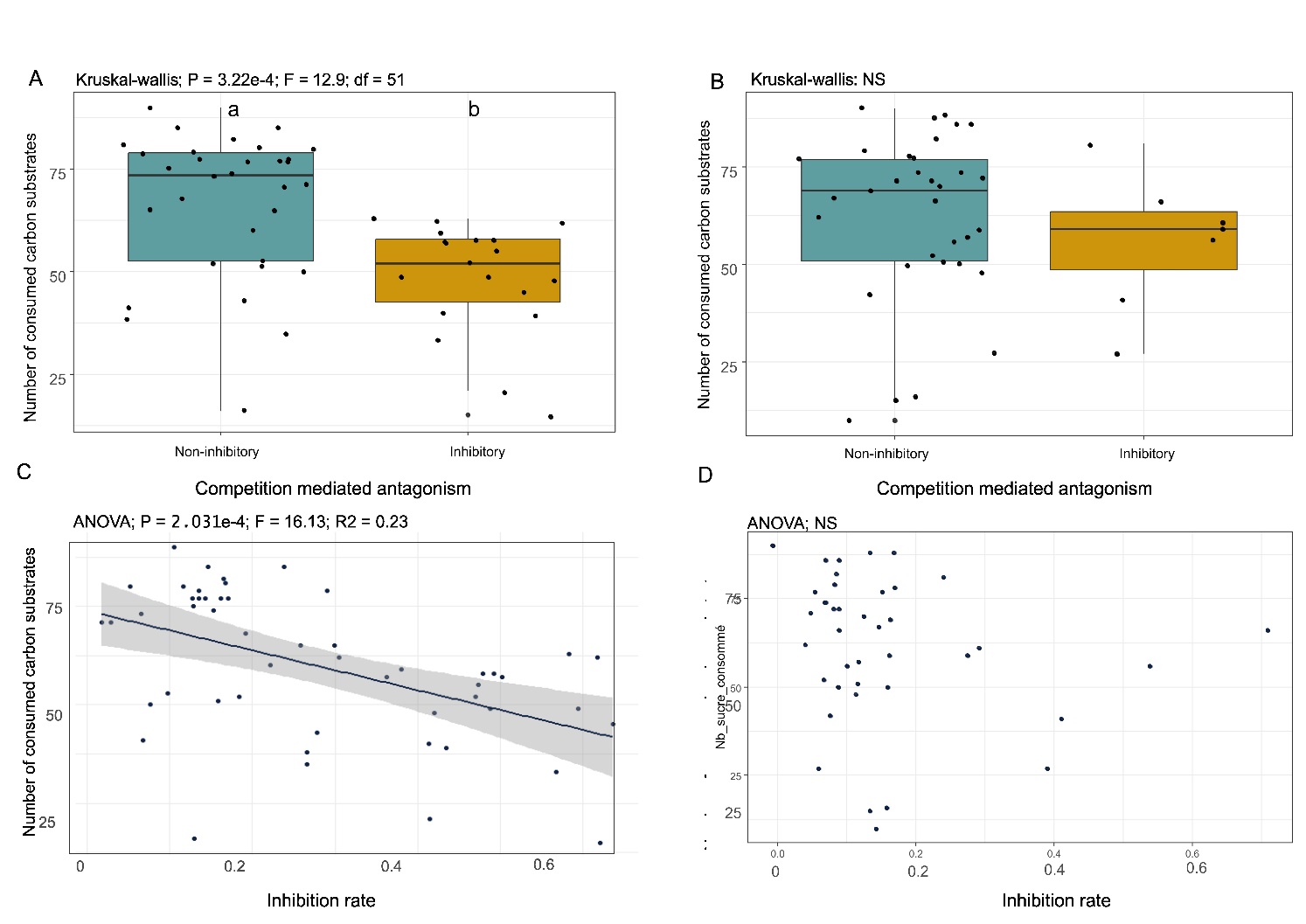


**Supplementary Figure 9.**  **Effect of inhibition capacity and antibiotic production on the metabolic versatility of oilseed rape and wheat.** (A, C) Comparison of the number of utilized carbon substrates between inhibitory (yellow) and non-inhibitory (blue) isolates from oilseed rape against Rs (A) and wheat against *Fg* (C), based on Kruskal–Wallis tests (P = 3.22e−4 for A; P > 0.05 for C). (B, D) Linear regression analyses of the relationship between inhibition rate (continuous variable) and the number of consumed carbon substrates in isolates from oilseed rape against Rs (B) and wheat against Fg (D). A significant negative correlation was found in B (ANOVA, P = 2.03e−4; F = 16.13; R² = 0.23), while no significant relationship was observed in D (P > 0.05).


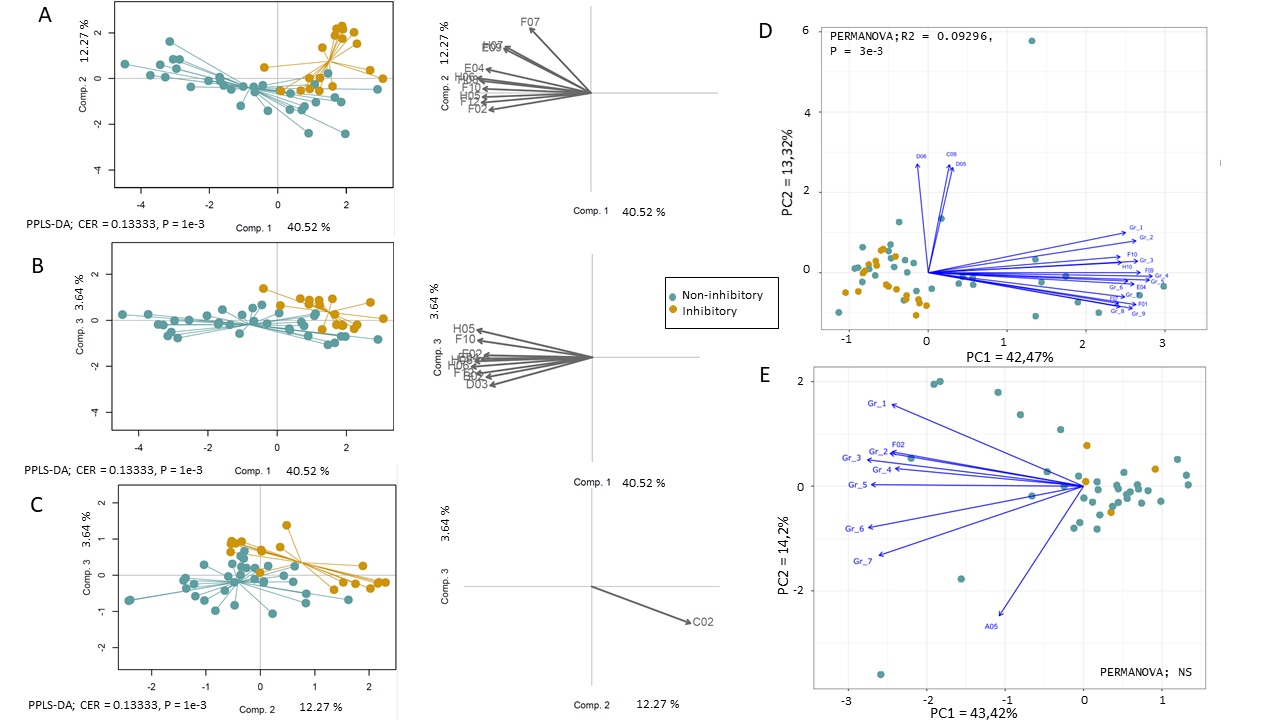


**Supplementary Figure 10.** **Differentiation of carbon substrate utilization of fungal isolates according to inhibition levels.** (A-C) Sparse Partial Least Squares Discriminant Analysis (sPLS-DA) illustrates the discrimination of samples based on inhibition strength (Inhibitory vs. Non-inhibitory) across different component combinations: (A) Component 1 vs. Component 2, (B) Component 1 vs. Component 3, and (C) Component 2 vs. Component 3. Each point represents a sample and is linked to its group centroid. Arrows indicate the most discriminative variables. The mean classification error rate (CER) is 12.4% ± 1.2 (based on 999 permutations), with a P of 0.001 indicating significant separation between groups. (D) Principal Coordinates Analysis (PCoA) based on carbon utilization profiles against *Rs*. Samples are colored by inhibition strength (Inhibitory vs. Non-inhibitory). Blue arrows indicate variable contributions to the ordination. Groups are specified in Table S4 and correspond to the carbon sources correlated with each other, PERMANOVA: R² =0.09, p = 0.003, showing significant compositional differences between inhibition levels. (E) Principal Coordinates Analysis (PCoA) based on microbial inhibition profiles against *Fg*, using the same structure as in (D), with vectors showing feature contributions.


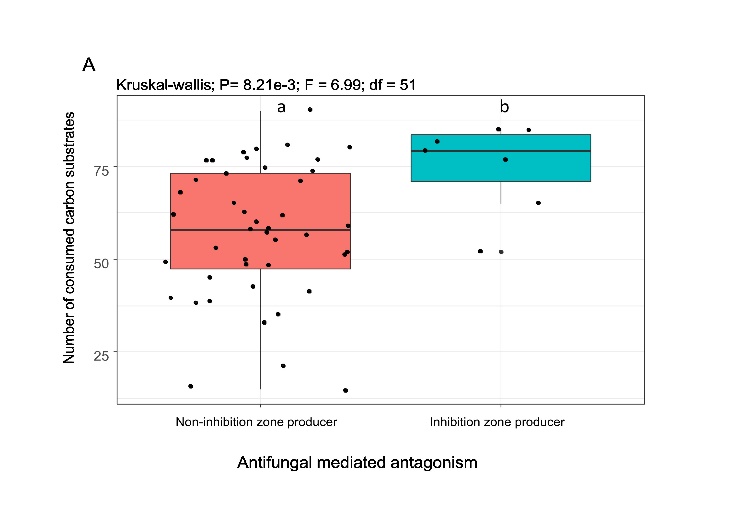


**Supplementary Figure 11.** **Effect of the number of consumed carbon substrates in isolates from oilseed rape on their ability to produce inhibition zones against Rs.** (A) Comparison of the number of consumed carbon substrates between inhibition zone producer isolates (turquoise) and non-producer isolates (red) isolates from oilseed rape and tested against Rs.


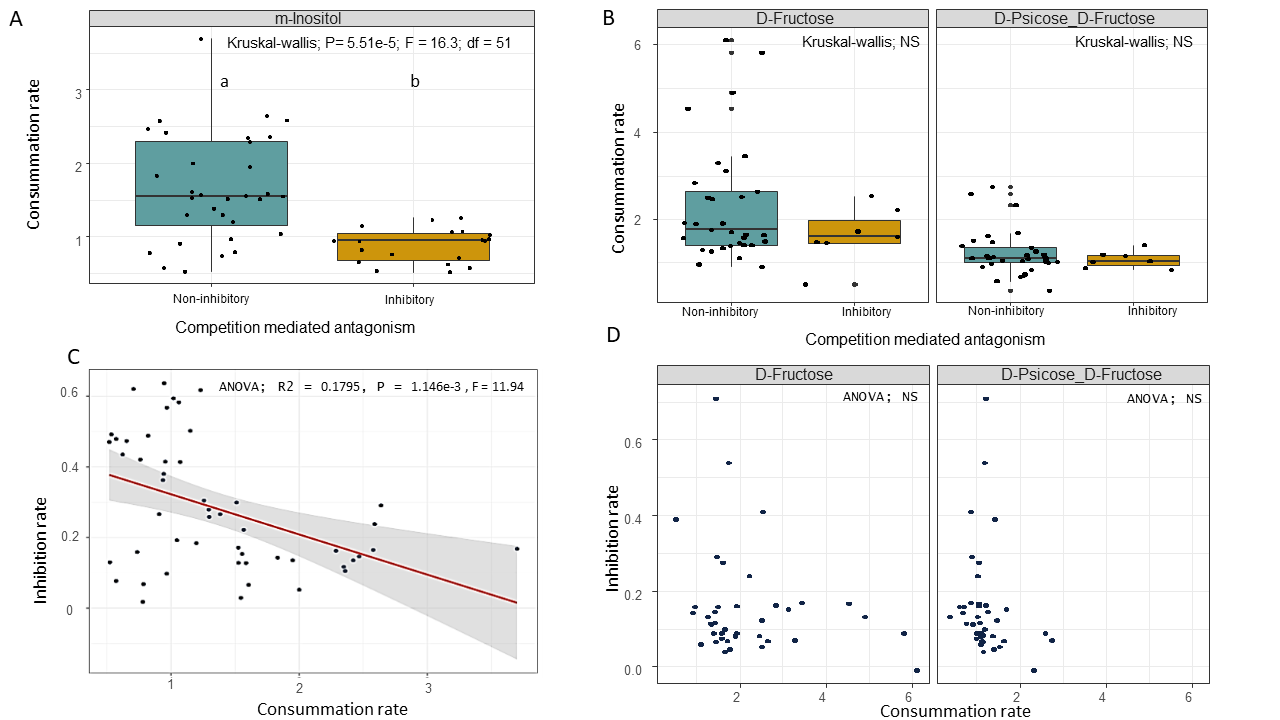


**Supplementary Figure 12.** **Relationship between carbon source consumption and fungal inhibition.** (A) Comparison of m-inositol consumption rates between inhibitory and non-inhibitory fungal isolates. A significant difference was observed (Kruskal–Wallis test; P = 5.51×10⁻⁵, F = 16.3, df = 51), with non-inhibitory isolates consuming significantly more m-inositol. Different letters indicate statistically significant differences (P < 0.05). (B) No significant differences in consumption rates of D-fructose and D-psicose_D-fructose between inhibitory and non-inhibitory isolates (Kruskal–Wallis; P > 0.05). (C) Correlation between m-inositol consumption and inhibition rate (ANOVA; R² = 0.1795, P = 0.00115, F = 11.94), suggesting a potential link between carbon source use and antifungal capacity. (D) No significant correlations were observed for D-fructose or D-psicose_D-fructose (ANOVA; P > 0.05).

# Supplementary Tables

**Supplementary Table 1.** Description of carbon sources and associated chemical guilds in Biolog FF microplates. Each well (Well) corresponds to a specific compound (SourceC) present in the Biolog FF microplate. Substrates are grouped into chemical guilds based on their molecular nature. The table also indicates whether each compound serves as a carbon source and/or a nitrogen source for fungal metabolism.

| **Well** | **SourceC** | **Chemical guild** | **Carbon substrate** | **Nitrogen substrate** |  | **Well** | **SourceC** | **Chemical guild** | **Source de carbone** | **source d'azote** |
| --- | --- | --- | --- | --- | --- | --- | --- | --- | --- | --- |
| A01 | Control | Control | - |  |  | E01 | D-Ribose | Carbohydrates | Yes | No |
| A02 | Tween 80 | Polymers | Yes | No |  | E02 | Salicin | Miscellaneous | Yes | No |
| A03 | N-Acetyl-D-Galactosamine | Carbohydrates | Yes | Yes |  | E03 | Sedoheptulosan | Carbohydrates | Yes | No |
| A04 | N-Acetyl-D-Glucosamine | Carbohydrates | Yes | Yes |  | E04 | D-Sorbitol | Carbohydrates | Yes | No |
| A05 | N-Acetyl-D-Mannosamine | Carbohydrates | Yes | Yes |  | E05 | L-Sorbose | Carbohydrates | Yes | No |
| A06 | Adonitol | Carbohydrates | Yes | No |  | E06 | Stachyose | Carbohydrates | Yes | No |
| A07 | Amygdalin | Miscellaneous | Yes | Yes |  | E07 | Sucrose | Carbohydrates | Yes | No |
| A08 | D-Arabinose | Carbohydrates | Yes | No |  | E08 | D-Tagatose | Carbohydrates | Yes | No |
| A09 | L-Arabinose | Carbohydrates | Yes | No |  | E09 | D-Trehalose | Carbohydrates | Yes | No |
| A10 | D-Arabitol | Carbohydrates | Yes | No |  | E10 | Turanose | Carbohydrates | Yes | No |
| A11 | Arbutin | Carbohydrates | Yes | No |  | E11 | Xylitol | Carbohydrates | Yes | No |
| A12 | D-Cellobiose | Carbohydrates | Yes | No |  | E12 | D-Xylose | Carbohydrates | Yes | No |
| B01 | α-Cyclodextrin | Polymers | Yes | No |  | F01 | γ-Amino butyric acid | Amino acids | Yes | Yes |
| B02 | β-Cyclodextrin | Polymers | Yes | No |  | F02 | Bromo_succinic-Acid | Miscellaneous | Yes | No |
| B03 | Dextrin | Polymers | Yes | No |  | F03 | Fumaric-Acid | Carboxylic acids | Yes | No |
| B04 | i-Erythritol | Carbohydrates | Yes | No |  | F04 | β-Hydroxy_butyric-Acid | Carboxylic acids | Yes | No |
| B05 | D-Fructose | Carbohydrates | Yes | No |  | F05 | Gamma-Hydoxybutyric-Acid | Carboxylic acids | Yes | No |
| B06 | L-Fucose | Carbohydrates | Yes | No |  | F06 | Rho-Hydroxy_phenylacetic-Acid | Carboxylic acids | Yes | No |
| B07 | D-Galactose | Carbohydrates | Yes | No |  | F07 | α-Keto-glutaric-Acid | Carboxylic acids | Yes | No |
| B08 | D-Galacturonic-Acid | Carboxylic acids | Yes | No |  | F08 | D-Lactic-Acid_Methyl_Ester | Miscellaneous | Yes | No |
| B09 | Gentiobiose | Carbohydrates | Yes | No |  | F09 | L-Lactic-Acid | Carboxylic acids | Yes | No |
| B10 | D-Gluconic-Acid | Carboxylic acids | Yes | No |  | F10 | D-Malic-Acid | Carboxylic acids | Yes | No |
| B11 | D-Glucosamine | Amines/amides | Yes | Yes |  | F11 | L-Malic-Acid | Carboxylic acids | Yes | No |
| B12 | α-D-Glucose | Carbohydrates | Yes | No |  | F12 | Quinic-Acid | Carboxylic acids | Yes | No |
| C01 | Glucose-1-Phosphate | Miscellaneous | Yes | No |  | G01 | D-Saccharic-Acid | Carboxylic acids | Yes | No |
| C02 | Glucuronamide | Amines/amides | Yes | Yes |  | G02 | Sebacic-Acid | Carboxylic acids | Yes | No |
| C03 | D-Glucuronic-Acid | Carboxylic acids | Yes | No |  | G03 | Succinamic-Acid | Amines/amides | Yes | Yes |
| C04 | Glycerol | Miscellaneous | Yes | No |  | G04 | Succinic-Acid | Carboxylic acids | Yes | No |
| C05 | Glycogen | Polymers | Yes | No |  | G05 | Succinic-Acid_Mono-Methyl-Ester | Miscellaneous | Yes | No |
| C06 | m-Inositol | Carbohydrates | Yes | No |  | G06 | N-Acethyl-L-Glutamic-Acid | Carboxylic acids | Yes | Yes |
| C07 | 2-Keto-D-Gluconic-Acid | Carboxylic acids | Yes | No |  | G07 | Alaninamide | Amines/amides | Yes | Yes |
| C08 | α-D-Lactose | Carbohydrates | Yes | No |  | G08 | L-Alanine | Amino acids | Yes | Yes |
| C09 | Lactulose | Carbohydrates | Yes | No |  | G09 | L-Alanyl-Glycine | Amino acids | Yes | Yes |
| C10 | Maltitol | Carbohydrates | Yes | No |  | G10 | L-Asparagine | Amino acids | Yes | Yes |
| C11 | Maltose | Carbohydrates | Yes | No |  | G11 | L-Aspartic-Acid | Amino acids | Yes | Yes |
| C12 | Maltotriose | Carbohydrates | Yes | No |  | G12 | L-Glutamic-Acid | Amino acids | Yes | Yes |
| D01 | D-Mannitol | Carbohydrates | Yes | No |  | H01 | Glycyl-L-Gutamic-Acid | Amino acids | Yes | Yes |
| D02 | D-Mannose | Carbohydrates | Yes | No |  | H02 | L-Ornithine | Amino acids | Yes | Yes |
| D03 | D-Melezitose | Carbohydrates | Yes | No |  | H03 | L-Phenylalanine | Amino acids | Yes | Yes |
| D04 | D-Melibiose | Carbohydrates | Yes | No |  | H04 | L-Proline | Amino acids | Yes | Yes |
| D05 | α-Methyl-D-Galactoside | Carbohydrates | Yes | No |  | H05 | L-Pyroglutamic-Acid | Amino acids | Yes | Yes |
| D06 | β-Methyl-D-Galactoside | Carbohydrates | Yes | No |  | H06 | L-Serine | Amino acids | Yes | Yes |
| D07 | α-Methyl-D-Glucoside | Carbohydrates | Yes | No |  | H07 | L-Threonine | Amino acids | Yes | Yes |
| D08 | β-Methyl-D-Glucoside | Carbohydrates | Yes | No |  | H08 | 2-Amino-Ethanol | Amines/amides | Yes | Yes |
| D09 | Palatinose | Carbohydrates | Yes | No |  | H09 | Putrescine | Amines/amides | Yes | Yes |
| D10 | D-Psicose_D-Fructose | Carbohydrates | Yes | No |  | H10 | Adenosine | Miscellaneous | Yes | No |
| D11 | D-Raffinose | Carbohydrates | Yes | No |  | H11 | Uridine | Miscellaneous | Yes | No |
| D12 | L-Rhamnose | Carbohydrates | Yes | No |  | H12 | Adenosine-5'-Monophosphate | Miscellaneous | Yes | No |

**Supplementary Table 2.** Metadata and taxonomic classification of fungal isolates selected from the culture collection. Each strain (STRAIN_ID) is annotated with its isolation compartment. Taxonomic affiliation was determined based on the consensus of two molecular markers (ITS1 and SSU). Assignments from kingdom to species level were obtained via BLAST searches against the NCBI nucleotide database for both barcodes.

| Strain  number | SSU Assignation | ITS1 Assignation | concencus phylum | concencus class | concencus order | concencus famille | Concencus genre |
| --- | --- | --- | --- | --- | --- | --- | --- |
| Bn_01 | Trichoderma lixii | Trichoderma gamsii | Ascomycota | Sordariomycetes | Hypocreales | Hypocreaceae | Trichoderma |
| Bn_03 | Trichoderma viride | Trichoderma koningiopsis | Ascomycota | Sordariomycetes | Hypocreales | Hypocreaceae | Trichoderma |
| Bn_04 | Trichoderma harzianum | Trichoderma tomentosum | Ascomycota | Sordariomycetes | Hypocreales | Hypocreaceae | Trichoderma |
| Bn_05 | Trichoderma lixii | Trichoderma sp. | Ascomycota | Sordariomycetes | Hypocreales | Hypocreaceae | Trichoderma |
| Bn_06 | Actinomucor elegans | Actinomucor elegans | Mucoromycota | Mucoromycetes | Mucorales | Mucoraceae | Actinomucor |
| Bn_07 | Mucor circinelloides | Mucoraceae sp. | Mucoromycota | Mucoromycetes | Mucorales | Mucoraceae | #N/A |
| Bn_08 | Absidia glauca | Absidia glauca | Mucoromycota | Mucoromycetes | Mucorales | Cunninghamellaceae | Absidia |
| Bn_09 | Cladosporium maltirimosum | Trichoderma tomentosum | Ascomycota | Dothideomycetes | Cladosporiales | Cladosporiaceae | #N/A |
| Bn_10 | Trichoderma polysporum | Trichoderma viridarium | Ascomycota | Sordariomycetes | Hypocreales | Hypocreaceae | Trichoderma |
| Bn_11 | Penicillium digitatum | Penicillium solitum | Ascomycota | Eurotiomycetes | Eurotiales | Aspergillaceae | Penicillium |
| Bn_13 | Exophiala equina | Exophiala sp. | Ascomycota | Eurotiomycetes | Chaetothyriales | Herpotrichiellaceae | Exophiala |
| Bn_14 | Epicoccum sorghinum | Epicoccum nigrum | Ascomycota | Dothideomycetes | Pleosporales | Didymellaceae | Epicoccum |
| Bn_15 | Cladosporium maltirimosum | Cladosporiaceae sp. | Ascomycota | Dothideomycetes | Cladosporiales | Cladosporiaceae | #N/A |
| Bn_16 | Fusarium sp. | Fusarium avenaceum | Ascomycota | Sordariomycetes | Hypocreales | Nectriaceae | Fusarium |
| Bn_17 | Penicillium soppii | Penicillium dipodomyicola | Ascomycota | Eurotiomycetes | Eurotiales | Aspergillaceae | Penicillium |
| Bn_18 | Epicoccum sorghinum | Clonostachys rosea | Ascomycota | Dothideomycetes | Pleosporales | #N/A | #N/A |
| Bn_19 | Mucor circinelloides | Mucoromycotina sp. | Mucoromycota | Mucoromycetes | Mucorales | Mucoraceae | #N/A |
| Bn_21 | Mortierella alpina | Mortierella alpina | Mucoromycota | Mortierellomycetes | Mortierellales | Mortierellaceae | Mortierella |
| Bn_22 | Penicillium roqueforti | Penicillium sp. | Ascomycota | Eurotiomycetes | Eurotiales | Aspergillaceae | Penicillium |
| Bn_23 | Torula longan | Torula acaciae | Ascomycota | Dothideomycetes | Pleosporales | Torulaceae | Torula |
| Bn_24 | Penicillium roqueforti | Penicillium janczewskii | Ascomycota | Eurotiomycetes | Eurotiales | Aspergillaceae | Penicillium |
| Bn_25 | Penicillium soppii | Penicillium sp. | Ascomycota | Eurotiomycetes | Eurotiales | Aspergillaceae | Penicillium |
| Bn_26 | Penicillium sp. | Penicillium sp. | Ascomycota | Eurotiomycetes | Eurotiales | Aspergillaceae | Penicillium |
| Bn_27 | Mortierella sp. | Mortierella elongata | Mucoromycota | Mortierellomycetes | Mortierellales | Mortierellaceae | Mortierella |
| Bn_28 | Fusarium oxysporum | Penicillium aurantiogriseum | Ascomycota | Sordariomycetes | Hypocreales | Nectriaceae | #N/A |
| Bn_29 | Penicillium sp. | Penicillium allii | Ascomycota | Eurotiomycetes | Eurotiales | Aspergillaceae | Penicillium |
| Bn_30 | Trichoderma harzianum | Trichoderma velutinum | Ascomycota | Sordariomycetes | Hypocreales | Hypocreaceae | Trichoderma |
| Bn_31 | Botrytis cinerea | Botrytis cinerea | Ascomycota | Leotiomycetes | Hypocreales | Sclerotiniaceae | Botrytis |
| Bn_32 | Penicillium digitatum | Penicillium glandicola | Ascomycota | Eurotiomycetes | Eurotiales | Aspergillaceae | Penicillium |
| Bn_33 | Penicillium digitatum | Penicillium griseofulvum | Ascomycota | Eurotiomycetes | Eurotiales | Aspergillaceae | Penicillium |
| Bn_34 | Penicillium digitatum | Penicillium janthinellum | Ascomycota | Eurotiomycetes | Eurotiales | Aspergillaceae | Penicillium |
| Bn_36 | Mortierella sp. | NA | #N/A | #N/A | #N/A | #N/A | #N/A |
| Bn_39 | Neonectria lugdunensis | Dactylonectria macrodidyma | Ascomycota | Sordariomycetes | Hypocreales | Nectriaceae | #N/A |
| Bn_41 | Penicillium digitatum | Penicillium sp. | Ascomycota | Eurotiomycetes | Eurotiales | Aspergillaceae | Penicillium |
| Bn_43 | Penicillium sp. | Cladosporium anthropophilum | Ascomycota | Eurotiomycetes | Eurotiales | Aspergillaceae | #N/A |
| Bn_44 | Penicillium digitatum | Penicillium sp. | Ascomycota | Eurotiomycetes | Eurotiales | Aspergillaceae | Penicillium |
| Bn_46 | Fusarium sporotrichioides | Fusarium cerealis | Ascomycota | Sordariomycetes | Hypocreales | Nectriaceae | Fusarium |
| Bn_47 | Penicillium digitatum | Penicillium sp. | Ascomycota | Eurotiomycetes | Eurotiales | Aspergillaceae | Penicillium |
| Bn_49 | Mucor circinelloides | Acremonium sp. | Mucoromycota | Mucoromycetes | Mucorales | Mucoraceae | #N/A |
| Bn_50 | Mucor circinelloides | Mucor circinelloides | Mucoromycota | Mucoromycetes | Mucorales | Mucoraceae | Mucor |
| Bn_52 | Mucor circinelloides | Mucoromycotina sp. | Mucoromycota | Mucoromycetes | Mucorales | Mucoraceae | #N/A |
| Bn_53 | Mucor circinelloides | Gibberella pulicaris | Mucoromycota | Mucoromycetes | Mucorales | Mucoraceae | #N/A |
| Bn_54 | Necromortierella dichotoma | Epicoccum nigrum | Mucoromycota | Mucoromycetes | Mortierellales | Mortierellaceae | #N/A |
| Bn_56 | Fusarium culmorum | Fungi | Mucoromycota | Mucoromycetes | Mucorales | Mucoraceae | Fusarium |
| Bn_59 | Cladosporium maltirimosum | Cladosporium sp. | Ascomycota | Dothideomycetes | Cladosporiales | Cladosporiaceae | Cladosporium |
| Bn_60 | Mucor circinelloides | Mucoraceae sp. | Mucoromycota | Mucoromycetes | Mucorales | Mucoraceae | #N/A |
| Bn_62 | Penicillium roqueforti | Penicillium griseofulvum | Ascomycota | Eurotiomycetes | Eurotiales | Aspergillaceae | Penicillium |
| Bn_63 | Pseudeurotium sp. | Pseudeurotium bakeri | Ascomycota | Leotiomycetes | Thelebolales | Pseudeurotiaceae | Pseudeurotium |
| Bn_64 | Acremonium antarcticum | Uncultured fungus | Ascomycota | Sordariomycetes | Pleosporales | #N/A | #N/A |
| Bn_67 | Pyrenochaeta nobilis | Pyrenochaetopsis sp. | Ascomycota | Dothideomycetes | Pleosporales | Pyrenochaetopsidaceae | Pyrenochaetopsis |
| Bn_68 | Penicillium sp. | Penicillium bialowiezense | Ascomycota | Eurotiomycetes | Eurotiales | Aspergillaceae | Penicillium |
| Ta_01 | Trichoderma lixii | Trichoderma sp. | Ascomycota | Sordariomycetes | Hypocreales | Hypocreaceae | Trichoderma |
| Ta_02 | Fusarium sporotrichioides | Chaetomium sp. | Ascomycota | Sordariomycetes | Hypocreales | Nectriaceae | #N/A |
| Ta_03 | Chaetopyrena penicillata | Epicoccum nigrum | Ascomycota | Dothideomycetes | Pleosporales | Didymellaceae | #N/A |
| Ta_04 | Penicillium roqueforti | Penicillium sp. | Ascomycota | Eurotiomycetes | Eurotiales | Aspergillaceae | Penicillium |
| Ta_05 | Penicillium soppii | Penicillium aurantiogriseum | Ascomycota | Eurotiomycetes | Eurotiales | Aspergillaceae | Penicillium |
| Ta_06 | Fusarium sp. | Fusarium acuminatum | Ascomycota | Sordariomycetes | Hypocreales | Nectriaceae | Fusarium |
| Ta_07 | Penicillium sp. | Penicillium sp. | Ascomycota | Eurotiomycetes | Eurotiales | Aspergillaceae | Penicillium |
| Ta_08 | Alternaria ethzedia | Alternaria infectoria | Ascomycota | Dothideomycetes | Pleosporales | Pleosporaceae | Alternaria |
| Ta_09 | Penicillium roqueforti | Penicillium dipodomyicola | Ascomycota | Eurotiomycetes | Eurotiales | Aspergillaceae | Penicillium |
| Ta_10 | Mortierella sp. | Mortierella gamsii | Mucoromycota | Mortierellomycetes | Mortierellales | Mortierellaceae | Mortierella |
| Ta_11 | Linnemannia sclerotiella | Linnemannia elongata | Mucoromycota | Mortierellomycetes | Mortierellales | Mortierellaceae | Linnemannia |
| Ta_12 | Alternaria poaceicola | Alternaria hordeiaustralica | Ascomycota | Dothideomycetes | Pleosporales | Pleosporaceae | Alternaria |
| Ta_13 | Penicillium roqueforti | Penicillium dipodomyicola | Ascomycota | Eurotiomycetes | Eurotiales | Aspergillaceae | Penicillium |
| Ta_14 | Fusarium equiseti | Fusarium sp. | Ascomycota | Sordariomycetes | Hypocreales | Nectriaceae | Fusarium |
| Ta_15 | Cladosporium maltirimosum | Cladosporium cladosporioides | Ascomycota | Dothideomycetes | Cladosporiales | Cladosporiaceae | Cladosporium |
| Ta_16 | Penicillium soppii | Penicillium scabrosum | Ascomycota | Eurotiomycetes | Eurotiales | Aspergillaceae | Penicillium |
| Ta_18 | Clonostachys kunmingensis | Penicillium canescens | Ascomycota | Sordariomycetes | Hypocreales | Bionectriaceae | #N/A |
| Ta_19 | Penicillium sp. | Penicillium christenseniae | Ascomycota | Eurotiomycetes | Eurotiales | Aspergillaceae | Penicillium |
| Ta_20 | Penicillium roqueforti | Penicillium freii | Ascomycota | Eurotiomycetes | Eurotiales | Aspergillaceae | Penicillium |
| Ta_21 | Alternaria ethzedia | Alternaria sp. | Ascomycota | Dothideomycetes | Pleosporales | Pleosporaceae | Alternaria |
| Ta_22 | Penicillium digitatum | Penicillium sp. | Ascomycota | Eurotiomycetes | Eurotiales | Aspergillaceae | Penicillium |
| Ta_23 | Cladosporium maltirimosum | Cladosporium sp. | Ascomycota | Dothideomycetes | Cladosporiales | Cladosporiaceae | Cladosporium |
| Ta_24 | Penicillium brevicompactum | Penicillium brevicompactum | Ascomycota | Eurotiomycetes | Eurotiales | Aspergillaceae | Penicillium |
| Ta_25 | Cladosporium maltirimosum | Cladosporium cf. | Ascomycota | Dothideomycetes | Cladosporiales | Cladosporiaceae | Cladosporium |
| Ta_26 | Clonostachys krabiensis | Penicillium aurantiocandidum | Ascomycota | Sordariomycetes | Hypocreales | Bionectriaceae | #N/A |
| Ta_27 | Mucor circinelloides | Mucor circinelloides | Mucoromycota | Mucoromycetes | Mucorales | Mucoraceae | Mucor |
| Ta_28 | Mucor hiemalis | Mucor hiemalis | Mucoromycota | Mucoromycetes | Mucorales | Mucoraceae | Mucor |
| Ta_29 | Fusarium solani | Nectria sp. | Ascomycota | Sordariomycetes | Hypocreales | Nectriaceae | #N/A |
| Ta_30 | Penicillium soppii | Penicillium aurantiogriseum | Ascomycota | Eurotiomycetes | Eurotiales | Aspergillaceae | Penicillium |
| Ta_31 | Fusarium proliferatum | Fusarium sp. | Ascomycota | Sordariomycetes | Hypocreales | Nectriaceae | Fusarium |
| Ta_32 | Chaetomium globosum | Penicillium polonicum | Ascomycota | Sordariomycetes | Sordariales | Chaetomiaceae | #N/A |
| Ta_33 | Penicillium soppii | Penicillium aurantiogriseum | Ascomycota | Eurotiomycetes | Eurotiales | Aspergillaceae | Penicillium |
| Ta_34 | Penicillium digitatum | Penicillium sp. | Ascomycota | Eurotiomycetes | Eurotiales | Aspergillaceae | Penicillium |
| Ta_35 | Trichoderma lixii | Cladosporium cf. | Ascomycota | Sordariomycetes | Hypocreales | Hypocreaceae | Trichoderma |
| Ta_36 | Penicillium digitatum | Penicillium sp. | Ascomycota | Eurotiomycetes | Eurotiales | Aspergillaceae | Penicillium |
| Ta_37 | Cladosporium maltirimosum | Cladosporium allicinum | Ascomycota | Dothideomycetes | Cladosporiales | Cladosporiaceae | Cladosporium |
| Ta_38 | Fusarium sporotrichioides | Fusarium cerealis | Ascomycota | Sordariomycetes | Hypocreales | Nectriaceae | Fusarium |
| Ta_39 | Clonostachys kunmingensis | Clonostachys rosea | Ascomycota | Sordariomycetes | Hypocreales | Bionectriaceae | Clonostachys |
| Ta_40 | Fusarium oxysporum | Fusarium sp. | Ascomycota | Sordariomycetes | Hypocreales | Nectriaceae | Fusarium |
| Ta_41 | Cladosporium maltirimosum | Cladosporium tenuissimum | Ascomycota | Dothideomycetes | Cladosporiales | Cladosporiaceae | Cladosporium |

**Supplementary Table 3.** Results of permutational multivariate analysis of variance (PERMANOVA) based on Euclidean distances of carbon utilization profiles (95 carbon substrates) among fungal isolates. Permutational multivariate analysis of variance (PERMANOVA) was used to assess whether fungal taxonomic levels (class, order, family) explain variation in carbon source utilization. Euclidean distances were calculated from consumption rates of multiple carbon substrates. The table reports the P-value (Pr(>F)), F-statistic (F), coefficient of determination (R²), sum of squares (SumOfSqs), and degrees of freedom (Df) for each taxonomic level

|  | **Pr(>F)** | **F** | **R2** | **SumOfSqs** | **Df** |
| --- | --- | --- | --- | --- | --- |
| Class | 0.002 | 3.3101 | 0.15983 | 1396.9 | 5 |
| order | 0.003 | 2.6072 | 0.2204 | 1926.3 | 9 |
| family | 0.003 | 2.3204 | 0.3113 | 2720.8 | 15 |
| genus | 0.021 | 1.8046 | 0.34801 | 3041.6 | 21 |
| species | 0.005 | 2.049 | 0.57956 | 5065.3 | 37 |
| Crop | 0.592 | 0.6524 | 0.00712 | 62.2 | 1 |

**Supplementary Table 4.** **Groups of carbon substrates with correlated consumption patterns.** Carbon sources were clustered into groups based on similarities in fungal consumption profiles across isolates. Substrates within each group showed significant positive correlations in utilization, suggesting potential functional or metabolic relatedness.

| Groupe | Substrates |
| --- | --- |
| Gr_1 | E02, A07 |
| Gr_2 | E03, A09, E12, A12 |
| Gr_3 | G04, F11, F03 |
| Gr_4 | B10, B03 |
| Gr_5 | H06, C04, C11, G03 |
| Gr_6 | H08, G01, D11, B09, C06, D01, D04, D08, D09, D12, E06, E07, E10, C03, C07, F12 |
| Gr_7 | H05, F06 |
| Gr_8 | H02, H03, H04, E09, A11 |
| Gr_9 | G12, G08, G10, G09, G11, A04, G02, G05, E01 |

**Supplementary Table 5. Utilization rates of different carbon substrates for all isolates.** This table shows the utilization rates of all carbon substrates studied for all isolates. These values were used to create Figure 2A. The carbon substrates are classified in the same order as in this figure.

| Isolates | Tween  80 | N-Acetyl-D-Galactos-  amine | N-Acetyl-D-Glucos-  amine | N-Acetyl-D-Mannos  amine | Adonitol | Amygdalin | D-Arabinose | L-Arabinose | D-Arabitol | Arbutin | D-Cellobiose |
| --- | --- | --- | --- | --- | --- | --- | --- | --- | --- | --- | --- |
| Bn_01 | 1.27 | 0.91 | 1.31 | 0.92 | 1.01 | 1.24 | 0.74 | 1.22 | 1.28 | 1.13 | 1.30 |
| Bn_10 | 1.69 | 0.74 | 1.01 | 0.77 | 0.90 | 1.50 | 0.67 | 1.61 | 1.73 | 1.85 | 1.61 |
| Bn_11 | 1.24 | 0.98 | 1.56 | 1.03 | 1.16 | 2.09 | 0.74 | 1.66 | 1.43 | 2.10 | 1.86 |
| Bn_13 | 1.46 | 0.76 | 1.79 | 0.62 | 0.99 | 0.72 | 0.96 | 1.27 | 1.09 | 0.89 | 1.29 |
| Bn_14 | 1.17 | 0.41 | 0.38 | 0.74 | 0.77 | 1.23 | 0.27 | 1.01 | 0.58 | 0.96 | 1.48 |
| Bn_15 | 1.59 | 0.91 | 2.57 | 0.92 | 0.86 | 1.46 | 0.84 | 1.86 | 1.64 | 2.80 | 2.28 |
| Bn_16 | 0.90 | 0.65 | 0.89 | 0.55 | 0.99 | 0.84 | 0.63 | 1.03 | 1.23 | 1.15 | 1.19 |
| Bn_17 | 2.92 | 0.55 | 4.36 | 0.56 | 0.91 | 2.54 | 1.05 | 3.22 | 1.29 | 3.87 | 4.17 |
| Bn_18 | 2.51 | 0.77 | 4.61 | 0.80 | 2.48 | 2.17 | 1.18 | 2.74 | 2.70 | 4.86 | 3.08 |
| Bn_19 | 1.69 | 0.95 | 3.95 | 1.02 | 2.01 | 1.23 | 1.12 | 2.07 | 2.26 | 1.99 | 1.93 |
| Bn_21 | 2.54 | 0.51 | 2.58 | 1.06 | 0.69 | 0.28 | 0.82 | 0.98 | 0.62 | 0.44 | 1.51 |
| Bn_22 | 1.79 | 0.57 | 2.06 | 0.53 | 0.92 | 1.74 | 1.28 | 2.18 | 1.18 | 2.27 | 2.31 |
| Bn_23 | 1.11 | 0.92 | 1.44 | 0.99 | 0.96 | 1.06 | 0.55 | 0.78 | 1.18 | 0.63 | 1.52 |
| Bn_24 | 1.51 | 1.27 | 1.53 | 0.95 | 1.70 | 1.61 | 0.97 | 1.49 | 1.15 | 1.45 | 1.63 |
| Bn_25 | 1.49 | 0.48 | 2.87 | 0.55 | 1.41 | 0.73 | 1.63 | 2.65 | 1.77 | 1.21 | 2.73 |
| Bn_26 | 2.55 | 1.16 | 3.20 | 0.59 | 2.91 | 2.91 | 1.29 | 3.90 | 2.02 | 4.70 | 3.36 |
| Bn_27 | 2.16 | 0.72 | 1.67 | 0.56 | 0.76 | 0.81 | 0.58 | 0.57 | 0.90 | 1.20 | 0.83 |
| Bn_28 | 0.95 | 1.09 | 1.41 | 1.08 | 1.27 | 1.36 | 0.82 | 1.51 | 1.12 | 1.29 | 1.65 |
| Bn_29 | 1.24 | 0.91 | 1.64 | 0.71 | 1.35 | 1.87 | 0.65 | 1.52 | 1.31 | 2.03 | 1.70 |
| Bn_03 | 0.91 | 0.56 | 1.14 | 0.70 | 0.81 | 1.24 | 0.57 | 0.88 | 1.11 | 0.95 | 0.95 |
| Bn_30 | 1.06 | 1.00 | 1.22 | 1.05 | 1.03 | 1.07 | 0.98 | 1.06 | 1.07 | 0.88 | 1.20 |
| Bn_31 | 1.82 | 0.72 | 1.14 | 0.53 | 0.73 | 0.96 | 1.00 | 1.43 | 0.76 | 2.12 | 1.70 |
| Bn_32 | 2.94 | 0.70 | 6.13 | 0.76 | 4.30 | 3.95 | 1.46 | 3.44 | 3.55 | 4.41 | 4.40 |
| Bn_33 | 3.12 | 0.72 | 4.98 | 0.73 | 1.13 | 3.08 | 1.24 | 3.90 | 1.65 | 4.29 | 5.02 |
| Bn_34 | 1.74 | 0.69 | 2.27 | 0.54 | 0.99 | 1.30 | 1.06 | 2.45 | 1.20 | 2.63 | 2.43 |
| Bn_36 | 2.93 | 0.81 | 3.89 | 1.93 | 1.85 | 1.44 | 0.68 | 2.31 | 2.08 | 3.92 | 3.09 |
| Bn_39 | 1.26 | 1.26 | 1.54 | 1.11 | 0.95 | 1.31 | 0.47 | 1.14 | 1.69 | 1.68 | 1.42 |
| Bn_04 | 1.44 | 1.23 | 1.52 | 1.03 | 1.01 | 1.23 | 1.01 | 1.37 | 1.36 | 0.83 | 1.54 |
| Bn_41 | 1.42 | 0.73 | 2.13 | 0.43 | 1.05 | 1.25 | 0.78 | 1.82 | 1.03 | 1.79 | 2.25 |
| Bn_43 | 1.51 | 0.93 | 1.71 | 1.49 | 1.18 | 1.55 | 0.67 | 1.17 | 1.18 | 1.90 | 1.51 |
| Bn_44 | 3.59 | 0.73 | 3.01 | 1.55 | 3.37 | 3.11 | 1.04 | 3.15 | 3.75 | 3.31 | 3.78 |
| Bn_46 | 0.99 | 0.82 | 1.04 | 0.80 | 0.91 | 0.67 | 0.93 | 1.08 | 0.75 | 0.81 | 1.09 |
| Bn_47 | 2.47 | 0.51 | 2.12 | 1.17 | 1.76 | 2.02 | 1.05 | 2.39 | 2.21 | 2.38 | 2.77 |
| Bn_49 | 3.43 | 0.74 | 5.32 | 0.83 | 1.50 | 1.26 | 0.98 | 2.96 | 3.47 | 2.50 | 2.35 |
| Bn_05 | 1.79 | 1.02 | 1.70 | 0.83 | 0.92 | 1.52 | 0.98 | 1.64 | 1.36 | 1.59 | 1.90 |
| Bn_50 | 1.56 | 1.22 | 3.37 | 1.04 | 2.20 | 1.00 | 0.95 | 1.95 | 2.04 | 1.76 | 1.72 |
| Bn_52 | 1.79 | 1.08 | 3.63 | 1.08 | 2.07 | 1.02 | 0.92 | 2.00 | 1.83 | 1.84 | 1.89 |
| Bn_53 | 1.48 | 1.09 | 3.09 | 0.99 | 1.55 | 0.99 | 0.95 | 1.60 | 1.78 | 1.74 | 1.45 |
| Bn_54 | 1.22 | 1.10 | 1.16 | 1.07 | 1.26 | 1.23 | 1.10 | 1.16 | 1.12 | 1.16 | 1.55 |
| Bn_56 | 1.20 | 0.92 | 2.35 | 1.50 | 1.48 | 0.97 | 0.68 | 1.25 | 1.35 | 1.18 | 1.07 |
| Bn_59 | 1.27 | 0.92 | 1.75 | 1.06 | 1.42 | 1.45 | 0.92 | 1.21 | 1.33 | 1.80 | 1.55 |
| Bn_06 | 2.21 | 0.64 | 4.35 | 0.59 | 1.49 | 1.15 | 0.86 | 1.86 | 1.67 | 2.16 | 1.86 |
| Bn_60 | 1.92 | 0.54 | 2.62 | 0.53 | 0.82 | 0.97 | 0.80 | 2.92 | 1.14 | 2.81 | 3.15 |
| Bn_62 | 3.24 | 0.76 | 5.51 | 0.77 | 1.23 | 4.00 | 1.77 | 4.31 | 1.62 | 5.26 | 5.45 |
| Bn_63 | 1.60 | 0.89 | 2.01 | 0.94 | 1.31 | 1.47 | 0.63 | 1.34 | 0.98 | 1.93 | 1.71 |
| Bn_64 | 1.13 | 0.94 | 1.22 | 1.01 | 1.07 | 1.06 | 0.42 | 1.25 | 1.04 | 1.09 | 1.32 |
| Bn_67 | 1.35 | 0.71 | 1.28 | 0.71 | 1.02 | 1.25 | 0.44 | 0.91 | 0.74 | 1.13 | 1.23 |
| Bn_68 | 2.37 | 0.75 | 1.95 | 0.71 | 2.35 | 1.76 | 1.11 | 3.30 | 1.34 | 3.40 | 2.26 |
| Bn_07 | 1.61 | 1.01 | 3.71 | 1.16 | 1.74 | 1.20 | 1.05 | 1.90 | 1.85 | 1.82 | 1.79 |
| Bn_08 | 0.76 | 0.80 | 1.74 | 1.07 | 1.22 | 1.48 | 0.43 | 0.42 | 1.27 | 1.52 | 1.29 |
| Bn_09 | 1.34 | 1.10 | 1.34 | 1.27 | 1.19 | 1.27 | 1.10 | 1.14 | 1.24 | 0.98 | 1.33 |
| Fg | 2.02 | 2.14 | 1.50 | 1.10 | 1.65 | 1.79 | 1.43 | 2.65 | 1.09 | 1.48 | 2.43 |
| Rs | 1.28 | 1.33 | 0.90 | 0.90 | 0.36 | 0.49 | 0.74 | 1.25 | 0.89 | 0.29 | 1.25 |
| Ta_01 | 1.37 | 1.24 | 1.55 | 1.13 | 1.10 | 1.41 | 1.07 | 1.35 | 1.43 | 1.41 | 1.55 |
| Ta_10 | 2.48 | 0.68 | 4.80 | 0.68 | 0.92 | 1.96 | 0.94 | 2.36 | 1.22 | 2.93 | 2.83 |
| Ta_11 | 2.83 | 0.83 | 3.66 | 0.72 | 1.00 | 0.79 | 0.51 | 0.45 | 0.83 | 1.51 | 0.96 |
| Ta_12 | 1.43 | 1.00 | 1.48 | 0.62 | 1.29 | 2.07 | 0.85 | 1.99 | 1.77 | 1.99 | 3.08 |
| Ta_13 | 2.89 | 0.74 | 5.84 | 0.71 | 1.38 | 3.78 | 1.81 | 4.62 | 1.92 | 4.94 | 5.16 |
| Ta_14 | 0.95 | 0.23 | 0.85 | 0.45 | 0.78 | 0.74 | 0.36 | 0.88 | 0.67 | 0.83 | 1.07 |
| Ta_15 | 2.31 | 1.14 | 2.24 | 0.95 | 1.12 | 2.69 | 1.19 | 2.17 | 1.88 | 2.38 | 2.77 |
| Ta_16 | 1.57 | 0.83 | 1.33 | 0.90 | 1.10 | 1.58 | 0.81 | 1.55 | 1.27 | 1.44 | 1.59 |
| Ta_18 | 2.14 | 0.80 | 3.41 | 0.73 | 1.96 | 1.76 | 1.62 | 2.38 | 2.54 | 3.52 | 2.43 |
| Ta_19 | 2.33 | 1.36 | 2.53 | 0.92 | 1.24 | 1.47 | 1.11 | 2.67 | 2.18 | 1.85 | 2.38 |
| Ta_02 | 2.60 | 0.93 | 1.40 | 1.12 | 0.73 | 2.07 | 1.52 | 1.89 | 0.65 | 0.90 | 2.21 |
| Ta_20 | 1.64 | 1.15 | 1.56 | 1.15 | 1.35 | 1.83 | 0.86 | 1.94 | 1.75 | 1.75 | 1.87 |
| Ta_21 | 1.07 | 0.46 | 1.37 | 0.53 | 1.57 | 1.87 | 0.76 | 1.41 | 1.24 | 1.39 | 1.94 |
| Ta_22 | 1.12 | 0.80 | 1.17 | 0.96 | 1.01 | 1.29 | 0.62 | 1.31 | 1.06 | 1.28 | 1.33 |
| Ta_23 | 1.70 | 1.14 | 1.50 | 1.00 | 1.34 | 1.44 | 0.86 | 1.54 | 1.23 | 2.10 | 1.88 |
| Ta_24 | 2.95 | 0.80 | 3.76 | 0.73 | 1.16 | 1.94 | 1.06 | 5.53 | 0.97 | 5.87 | 4.41 |
| Ta_25 | 1.14 | 0.90 | 0.94 | 0.87 | 0.72 | 0.88 | 0.66 | 0.82 | 0.97 | 1.02 | 1.33 |
| Ta_26 | 1.21 | 0.99 | 1.31 | 0.98 | 1.10 | 1.23 | 0.72 | 1.37 | 1.16 | 1.08 | 1.37 |
| Ta_27 | 1.48 | 1.07 | 3.15 | 0.97 | 1.76 | 0.87 | 1.07 | 1.87 | 1.97 | 1.74 | 1.71 |
| Ta_28 | 1.97 | 0.91 | 2.51 | 0.93 | 1.87 | 1.13 | 1.09 | 1.62 | 1.95 | 1.78 | 1.69 |
| Ta_29 | 1.39 | 0.89 | 1.40 | 0.89 | 1.25 | 1.31 | 0.72 | 1.45 | 1.51 | 1.08 | 1.23 |
| Ta_03 | 0.65 | 0.52 | 0.50 | 0.99 | 0.44 | 0.60 | 0.22 | 0.90 | 0.70 | 0.94 | 0.95 |
| Ta_30 | 1.15 | 0.97 | 1.28 | 1.23 | 0.97 | 1.10 | 0.93 | 1.36 | 1.02 | 1.03 | 1.54 |
| Ta_31 | 1.69 | 1.53 | 1.48 | 0.86 | 1.17 | 1.02 | 1.02 | 1.94 | 1.86 | 1.50 | 2.10 |
| Ta_32 | 1.66 | 1.13 | 1.38 | 1.12 | 1.16 | 1.56 | 1.03 | 1.74 | 1.40 | 1.39 | 1.73 |
| Ta_33 | 1.27 | 1.14 | 1.45 | 1.10 | 1.09 | 1.56 | 0.95 | 1.46 | 1.21 | 1.17 | 1.62 |
| Ta_34 | 1.12 | 1.01 | 1.22 | 1.02 | 0.99 | 1.33 | 0.87 | 1.32 | 1.06 | 1.10 | 1.43 |
| Ta_35 | 1.29 | 0.95 | 1.16 | 0.89 | 0.82 | 1.28 | 0.83 | 1.41 | 1.03 | 1.01 | 1.45 |
| Ta_36 | 1.16 | 0.96 | 1.23 | 0.94 | 1.13 | 1.36 | 0.66 | 1.22 | 1.02 | 1.30 | 1.27 |
| Ta_37 | 3.87 | 2.30 | 1.58 | 2.56 | 3.09 | 1.73 | 1.34 | 5.33 | 3.69 | 4.19 | 5.56 |
| Ta_38 | 1.55 | 0.23 | 1.44 | 0.75 | 0.76 | 0.85 | 1.07 | 1.01 | 1.44 | 0.85 | 1.29 |
| Ta_39 | 2.47 | 0.82 | 2.95 | 0.75 | 1.88 | 1.52 | 1.61 | 2.52 | 2.34 | 3.26 | 1.98 |
| Ta_04 | 1.37 | 0.94 | 1.14 | 0.89 | 1.21 | 1.36 | 0.90 | 1.47 | 1.12 | 1.16 | 1.46 |
| Ta_40 | 1.35 | 0.77 | 1.49 | 0.85 | 1.11 | 1.36 | 0.59 | 1.36 | 1.36 | 1.29 | 1.33 |
| Ta_41 | 2.48 | 1.22 | 3.10 | 1.37 | 2.40 | 2.10 | 1.49 | 2.57 | 2.06 | 3.13 | 3.02 |
| Ta_05 | 1.47 | 1.09 | 1.27 | 1.18 | 1.05 | 1.62 | 0.97 | 1.66 | 1.48 | 1.38 | 1.76 |
| Ta_06 | 1.12 | 0.63 | 1.84 | 0.87 | 1.90 | 1.32 | 0.77 | 1.78 | 1.85 | 1.25 | 1.78 |
| Ta_07 | 1.72 | 0.99 | 1.64 | 1.19 | 1.32 | 1.59 | 0.82 | 1.73 | 1.22 | 1.76 | 1.91 |
| Ta_08 | 0.63 | 0.41 | 0.63 | 0.50 | 0.74 | 1.19 | 0.44 | 0.92 | 0.75 | 0.83 | 0.96 |
| Ta_09 | 3.18 | 0.82 | 6.24 | 0.63 | 1.27 | 4.03 | 1.92 | 5.04 | 1.91 | 5.18 | 5.29 |

| Isolates | α-Cyclodextrin | β-Cyclodextrin | Dextrin | i-Erythritol | D-Fructose | L-Fucose | D-Galactose | D-Galacturonic-Acid | Gentiobiose | D-Gluconic-Acid | D-Glucosamine |
| --- | --- | --- | --- | --- | --- | --- | --- | --- | --- | --- | --- |
| Bn_01 | 0.64 | 0.44 | 1.19 | 1.27 | 1.29 | 1.01 | 1.12 | 0.37 | 1.19 | 0.37 | 0.88 |
| Bn_10 | 0.95 | 0.71 | 1.36 | 1.71 | 1.77 | 1.60 | 1.65 | 0.45 | 1.80 | 0.42 | 0.63 |
| Bn_11 | 1.01 | 1.14 | 1.33 | 1.35 | 1.92 | 1.03 | 1.38 | 1.02 | 1.49 | 1.28 | 0.63 |
| Bn_13 | 1.47 | 0.93 | 0.85 | 0.78 | 0.78 | 0.49 | 0.78 | 0.25 | 0.82 | 0.45 | 0.35 |
| Bn_14 | 0.70 | 0.46 | 0.58 | 1.13 | 1.43 | 0.69 | 1.27 | 0.10 | 1.01 | 0.52 | 0.21 |
| Bn_15 | 1.16 | 0.81 | 2.10 | 1.80 | 2.08 | 0.25 | 2.29 | 0.23 | 2.01 | 2.45 | 1.06 |
| Bn_16 | 0.72 | 0.51 | 0.88 | 0.66 | 0.85 | 0.82 | 1.00 | 0.23 | 1.04 | 1.06 | 0.54 |
| Bn_17 | 2.26 | 1.80 | 2.95 | 2.66 | 3.82 | 0.44 | 2.98 | 0.88 | 1.71 | 4.03 | 1.66 |
| Bn_18 | 1.94 | 1.58 | 2.59 | 2.79 | 2.56 | 1.19 | 2.07 | 0.42 | 2.40 | 4.56 | 1.33 |
| Bn_19 | 1.28 | 0.81 | 1.68 | 1.07 | 1.75 | 0.43 | 1.99 | 0.32 | 1.45 | 2.65 | 1.12 |
| Bn_21 | 1.57 | 0.75 | 0.26 | 0.43 | 2.58 | 0.34 | 2.25 | 0.19 | 1.04 | 0.61 | 1.80 |
| Bn_22 | 1.23 | 1.10 | 1.80 | 0.82 | 2.26 | 0.94 | 1.54 | 1.89 | 2.11 | 2.25 | 1.71 |
| Bn_23 | 0.86 | 0.66 | 0.54 | 0.80 | 1.06 | 1.15 | 1.44 | 0.14 | 1.17 | 0.84 | 0.38 |
| Bn_24 | 0.87 | 0.77 | 1.40 | 0.82 | 1.56 | 1.10 | 1.06 | 0.57 | 1.28 | 1.44 | 0.89 |
| Bn_25 | 2.51 | 1.23 | 1.90 | 1.00 | 2.50 | 0.39 | 0.88 | 0.76 | 1.46 | 2.31 | 1.00 |
| Bn_26 | 1.83 | 2.23 | 3.31 | 1.37 | 3.36 | 0.92 | 2.04 | 2.49 | 2.52 | 3.87 | 0.79 |
| Bn_27 | 1.50 | 0.77 | 0.72 | 0.56 | 2.70 | 0.44 | 2.11 | 0.21 | 0.88 | 0.52 | 2.02 |
| Bn_28 | 0.72 | 0.69 | 1.24 | 1.08 | 1.63 | 1.14 | 1.56 | 0.46 | 1.45 | 1.35 | 0.95 |
| Bn_29 | 1.32 | 1.24 | 1.37 | 1.21 | 1.70 | 0.78 | 1.17 | 0.89 | 1.61 | 1.39 | 0.78 |
| Bn_03 | 0.50 | 0.36 | 0.97 | 1.02 | 1.09 | 0.59 | 0.97 | 0.31 | 1.06 | 0.50 | 0.74 |
| Bn_30 | 0.73 | 0.55 | 1.13 | 1.03 | 1.15 | 0.94 | 1.11 | 0.24 | 1.15 | 0.85 | 0.93 |
| Bn_31 | 1.48 | 1.31 | 1.72 | 1.10 | 1.43 | 0.43 | 1.41 | 0.84 | 0.95 | 1.28 | 0.98 |
| Bn_32 | 2.58 | 2.38 | 2.75 | 1.90 | 4.14 | 0.69 | 2.10 | 0.73 | 2.54 | 4.90 | 2.99 |
| Bn_33 | 2.45 | 2.01 | 3.33 | 3.15 | 4.70 | 0.53 | 3.36 | 0.94 | 2.36 | 5.19 | 2.19 |
| Bn_34 | 1.25 | 1.40 | 1.89 | 0.97 | 2.34 | 0.71 | 1.92 | 1.64 | 2.08 | 2.69 | 1.67 |
| Bn_36 | 1.25 | 0.82 | 3.40 | 1.16 | 2.73 | 0.75 | 2.67 | 0.23 | 2.57 | 3.80 | 2.69 |
| Bn_39 | 1.10 | 0.73 | 0.82 | 1.53 | 1.40 | 0.42 | 1.39 | 0.45 | 1.04 | 0.72 | 0.31 |
| Bn_04 | 0.84 | 0.70 | 1.27 | 1.16 | 1.47 | 0.92 | 1.32 | 0.34 | 1.32 | 0.65 | 1.03 |
| Bn_41 | 1.17 | 1.12 | 1.36 | 0.99 | 1.84 | 0.58 | 1.35 | 1.37 | 1.70 | 2.05 | 1.21 |
| Bn_43 | 0.90 | 0.68 | 1.10 | 1.63 | 1.68 | 1.09 | 1.79 | 0.30 | 1.67 | 1.48 | 0.87 |
| Bn_44 | 2.30 | 3.45 | 3.87 | 2.70 | 4.20 | 0.69 | 3.10 | 1.56 | 3.57 | 3.35 | 1.09 |
| Bn_46 | 0.59 | 0.79 | 0.79 | 1.01 | 1.14 | 0.50 | 0.79 | 0.34 | 1.05 | 0.93 | 0.78 |
| Bn_47 | 1.64 | 2.13 | 2.15 | 1.60 | 2.39 | 0.59 | 1.61 | 0.92 | 2.26 | 2.40 | 0.57 |
| Bn_49 | 1.80 | 0.87 | 2.75 | 0.97 | 3.42 | 0.74 | 3.30 | 0.32 | 1.82 | 5.64 | 2.62 |
| Bn_05 | 0.84 | 0.73 | 1.50 | 1.41 | 1.52 | 1.16 | 1.67 | 0.43 | 1.67 | 0.74 | 0.81 |
| Bn_50 | 1.12 | 0.76 | 1.77 | 1.16 | 1.53 | 0.41 | 1.76 | 0.37 | 1.48 | 2.42 | 1.39 |
| Bn_52 | 0.91 | 0.73 | 1.66 | 1.17 | 1.37 | 0.38 | 1.88 | 0.29 | 1.38 | 2.59 | 1.22 |
| Bn_53 | 0.95 | 0.60 | 1.58 | 1.23 | 1.47 | 0.43 | 1.78 | 0.28 | 1.17 | 1.90 | 1.03 |
| Bn_54 | 0.91 | 0.93 | 0.86 | 1.15 | 1.43 | 1.07 | 1.28 | 0.22 | 1.58 | 1.31 | 0.27 |
| Bn_56 | 0.75 | 0.45 | 1.16 | 0.96 | 1.04 | 0.47 | 1.02 | 0.37 | 0.88 | 1.88 | 0.75 |
| Bn_59 | 0.81 | 0.61 | 0.97 | 1.45 | 2.08 | 0.76 | 1.87 | 0.35 | 1.67 | 1.71 | 0.73 |
| Bn_06 | 1.35 | 0.75 | 1.69 | 1.14 | 1.45 | 0.44 | 1.40 | 0.23 | 1.07 | 0.49 | 1.14 |
| Bn_60 | 1.31 | 1.43 | 1.91 | 0.94 | 2.85 | 0.44 | 1.60 | 1.68 | 2.61 | 3.07 | 1.98 |
| Bn_62 | 2.40 | 1.65 | 3.83 | 3.18 | 4.63 | 0.67 | 4.18 | 0.86 | 2.25 | 4.94 | 1.89 |
| Bn_63 | 1.21 | 1.22 | 1.44 | 0.98 | 1.59 | 0.51 | 1.40 | 0.17 | 1.30 | 0.74 | 0.71 |
| Bn_64 | 0.67 | 0.45 | 1.24 | 1.17 | 1.32 | 0.99 | 1.20 | 0.12 | 1.16 | 1.00 | 1.18 |
| Bn_67 | 1.01 | 0.80 | 0.65 | 1.34 | 1.54 | 0.26 | 1.76 | 0.35 | 1.15 | 0.40 | 0.33 |
| Bn_68 | 1.72 | 1.57 | 2.18 | 0.65 | 2.93 | 0.91 | 1.78 | 1.72 | 1.65 | 2.89 | 0.66 |
| Bn_07 | 1.30 | 0.84 | 1.73 | 1.04 | 1.64 | 0.45 | 1.83 | 0.35 | 1.31 | 2.41 | 1.13 |
| Bn_08 | 0.62 | 0.44 | 0.89 | 0.70 | 1.56 | 0.68 | 1.14 | 0.15 | 1.30 | 0.56 | 1.00 |
| Bn_09 | 0.85 | 0.71 | 1.22 | 1.26 | 1.37 | 1.18 | 1.22 | 0.41 | 1.20 | 0.89 | 0.90 |
| Fg | 1.18 | 1.61 | 2.53 | 0.91 | 0.95 | 2.66 | 1.29 | 0.46 | 1.60 | 1.29 | 1.09 |
| Rs | 0.70 | 0.65 | 0.74 | 0.46 | 1.17 | 0.81 | 1.21 | 0.11 | 0.67 | 0.69 | 0.20 |
| Ta_01 | 0.79 | 0.54 | 1.31 | 1.40 | 1.46 | 1.14 | 1.43 | 0.41 | 1.35 | 0.66 | 1.10 |
| Ta_10 | 2.37 | 1.51 | 2.63 | 2.12 | 3.45 | 0.52 | 2.10 | 0.62 | 1.45 | 3.50 | 1.62 |
| Ta_11 | 1.24 | 0.59 | 1.32 | 0.89 | 2.53 | 0.45 | 2.54 | 0.18 | 0.78 | 0.60 | 1.69 |
| Ta_12 | 1.19 | 0.92 | 1.60 | 2.44 | 2.83 | 0.80 | 0.50 | 0.21 | 2.90 | 0.58 | 0.76 |
| Ta_13 | 3.24 | 2.32 | 4.18 | 3.47 | 4.53 | 0.64 | 4.22 | 1.46 | 2.57 | 4.89 | 1.99 |
| Ta_14 | 0.71 | 0.63 | 0.75 | 0.45 | 0.93 | 0.27 | 0.86 | 0.09 | 0.88 | 0.90 | 0.57 |
| Ta_15 | 1.40 | 0.98 | 1.63 | 2.02 | 2.65 | 0.52 | 2.17 | 0.72 | 2.21 | 2.05 | 1.43 |
| Ta_16 | 0.73 | 0.97 | 1.52 | 1.34 | 1.70 | 0.84 | 1.56 | 0.72 | 1.39 | 1.49 | 1.18 |
| Ta_18 | 1.49 | 1.35 | 2.06 | 2.69 | 2.47 | 1.03 | 1.80 | 0.48 | 2.02 | 3.56 | 1.07 |
| Ta_19 | 1.24 | 1.31 | 2.63 | 2.22 | 3.11 | 0.68 | 2.03 | 1.67 | 1.97 | 2.63 | 1.35 |
| Ta_02 | 2.73 | 2.97 | 2.49 | 1.74 | 2.51 | 1.68 | 0.40 | 0.33 | 1.20 | 0.40 | 0.59 |
| Ta_20 | 0.73 | 0.98 | 1.60 | 1.54 | 1.93 | 1.11 | 1.75 | 0.63 | 1.73 | 1.64 | 1.34 |
| Ta_21 | 0.76 | 0.56 | 0.80 | 1.55 | 1.50 | 0.39 | 0.93 | 0.34 | 1.67 | 0.34 | 0.66 |
| Ta_22 | 0.48 | 0.65 | 1.11 | 0.92 | 1.34 | 0.61 | 1.23 | 0.56 | 1.22 | 1.23 | 0.86 |
| Ta_23 | 0.93 | 0.84 | 1.27 | 1.31 | 1.90 | 1.08 | 1.89 | 0.43 | 1.38 | 1.71 | 0.96 |
| Ta_24 | 2.70 | 2.71 | 4.84 | 1.30 | 5.82 | 0.83 | 2.10 | 3.23 | 2.18 | 5.15 | 1.33 |
| Ta_25 | 0.85 | 0.69 | 1.19 | 0.92 | 1.11 | 0.29 | 0.39 | 0.10 | 1.12 | 0.94 | 0.53 |
| Ta_26 | 0.63 | 0.81 | 1.18 | 1.12 | 1.40 | 0.83 | 1.22 | 0.46 | 1.26 | 1.01 | 0.96 |
| Ta_27 | 1.04 | 0.71 | 1.63 | 1.09 | 1.41 | 0.39 | 1.65 | 0.76 | 1.45 | 2.29 | 1.21 |
| Ta_28 | 0.75 | 0.53 | 1.63 | 1.00 | 1.73 | 0.86 | 1.66 | 0.36 | 1.39 | 0.99 | 1.00 |
| Ta_29 | 0.62 | 0.35 | 1.28 | 1.24 | 1.41 | 1.19 | 1.08 | 0.28 | 1.13 | 0.79 | 0.30 |
| Ta_03 | 0.63 | 0.46 | 0.72 | 0.69 | 1.27 | 0.73 | 1.17 | 0.15 | 1.09 | 0.52 | 0.15 |
| Ta_30 | 0.74 | 0.69 | 1.24 | 0.77 | 1.58 | 1.11 | 1.51 | 0.46 | 1.33 | 1.41 | 0.70 |
| Ta_31 | 1.02 | 1.34 | 1.51 | 1.35 | 2.21 | 0.99 | 1.05 | 0.38 | 1.24 | 1.99 | 0.82 |
| Ta_32 | 0.70 | 1.19 | 1.77 | 1.20 | 1.90 | 1.12 | 1.52 | 0.82 | 1.54 | 1.52 | 1.23 |
| Ta_33 | 0.79 | 0.99 | 1.04 | 1.29 | 1.65 | 1.15 | 1.54 | 0.55 | 1.43 | 1.41 | 1.06 |
| Ta_34 | 0.79 | 0.76 | 1.08 | 1.15 | 1.46 | 0.98 | 1.37 | 0.51 | 1.33 | 1.27 | 0.90 |
| Ta_35 | 0.67 | 0.99 | 1.55 | 1.14 | 1.65 | 1.02 | 1.29 | 0.67 | 1.22 | 1.28 | 1.01 |
| Ta_36 | 0.56 | 0.70 | 1.03 | 1.16 | 1.31 | 0.66 | 1.22 | 0.70 | 1.20 | 1.22 | 0.82 |
| Ta_37 | 2.75 | 2.40 | 3.87 | 1.89 | 6.11 | 0.47 | 2.10 | 0.50 | 5.89 | 4.66 | 1.63 |
| Ta_38 | 0.67 | 0.94 | 0.77 | 0.66 | 0.51 | 0.77 | 1.44 | 0.38 | 1.63 | 1.04 | 0.64 |
| Ta_39 | 1.35 | 1.19 | 2.06 | 2.68 | 2.51 | 1.15 | 1.49 | 0.54 | 1.78 | 2.78 | 0.94 |
| Ta_04 | 0.67 | 0.84 | 1.43 | 1.31 | 1.58 | 0.87 | 1.36 | 0.68 | 1.27 | 1.35 | 1.01 |
| Ta_40 | 0.71 | 0.63 | 1.34 | 0.74 | 1.47 | 0.94 | 1.32 | 1.06 | 1.29 | 1.16 | 1.03 |
| Ta_41 | 1.16 | 1.19 | 2.08 | 2.87 | 3.29 | 2.03 | 3.20 | 0.38 | 3.16 | 2.49 | 1.65 |
| Ta_05 | 0.81 | 0.86 | 1.39 | 1.05 | 1.76 | 1.20 | 1.66 | 0.50 | 1.45 | 1.53 | 0.90 |
| Ta_06 | 0.87 | 0.68 | 1.51 | 1.21 | 1.60 | 0.86 | 1.00 | 0.23 | 1.93 | 0.91 | 0.95 |
| Ta_07 | 0.80 | 0.77 | 1.54 | 1.21 | 1.91 | 1.00 | 1.57 | 0.66 | 1.67 | 1.74 | 0.83 |
| Ta_08 | 0.60 | 0.47 | 0.59 | 1.04 | 0.97 | 0.26 | 0.12 | 0.26 | 0.83 | 0.25 | 0.29 |
| Ta_09 | 3.44 | 2.63 | 4.44 | 3.76 | 4.92 | 0.65 | 4.52 | 1.85 | 2.62 | 5.25 | 2.16 |

| Isolates | α-D-Glucose | Glucose-1-Phosphate | Glucuronamide | D-Glucuronic-Acid | Glycerol | Glycogen | m-Inositol | 2-Keto-D-Gluconic-Acid | α-D-Lactose | Lactulose | Maltitol | Maltose |
| --- | --- | --- | --- | --- | --- | --- | --- | --- | --- | --- | --- | --- |
| Bn_01 | 1.24 | 0.92 | 0.15 | 0.82 | 1.18 | 1.21 | 0.95 | 1.10 | 1.06 | 0.95 | 0.88 | 0.97 |
| Bn_10 | 1.86 | 1.22 | 0.26 | 0.69 | 1.49 | 1.93 | 1.02 | 1.28 | 1.21 | 0.98 | 0.86 | 1.01 |
| Bn_11 | 2.01 | 1.29 | 0.19 | 1.58 | 2.07 | 1.69 | 1.52 | 1.81 | 1.76 | 0.87 | 1.13 | 1.79 |
| Bn_13 | 0.92 | 0.59 | 0.46 | 0.72 | 0.68 | 0.73 | 0.52 | 0.52 | 0.48 | 0.39 | 0.60 | 0.84 |
| Bn_14 | 1.47 | 0.36 | 0.14 | 1.38 | 1.07 | 1.41 | 1.29 | 1.43 | 1.12 | 0.87 | 1.13 | 1.37 |
| Bn_15 | 2.30 | 1.54 | 0.26 | 1.49 | 1.10 | 1.63 | 1.53 | 2.12 | 2.12 | 1.74 | 1.00 | 1.55 |
| Bn_16 | 1.14 | 0.49 | 0.18 | 0.69 | 0.81 | 0.86 | 0.91 | 1.01 | 0.75 | 1.08 | 1.11 | 0.86 |
| Bn_17 | 4.53 | 1.53 | 1.03 | 3.06 | 3.35 | 2.74 | 1.95 | 2.71 | 1.33 | 0.70 | 1.29 | 3.38 |
| Bn_18 | 2.82 | 1.33 | 0.46 | 4.44 | 3.29 | 3.20 | 2.35 | 4.52 | 0.72 | 0.70 | 0.73 | 2.32 |
| Bn_19 | 2.20 | 1.24 | 1.18 | 0.77 | 1.43 | 1.67 | 0.57 | 0.72 | 0.64 | 0.62 | 0.85 | 1.73 |
| Bn_21 | 2.11 | 0.54 | 0.63 | 1.88 | 0.43 | 2.40 | 1.38 | 0.40 | 0.85 | 0.64 | 0.81 | 1.50 |
| Bn_22 | 2.24 | 1.44 | 0.28 | 2.05 | 2.12 | 1.57 | 1.59 | 2.31 | 1.05 | 1.02 | 1.25 | 2.19 |
| Bn_23 | 1.49 | 0.85 | 0.65 | 1.41 | 0.79 | 1.01 | 0.78 | 1.06 | 1.39 | 1.29 | 0.98 | 1.09 |
| Bn_24 | 1.68 | 0.81 | 0.12 | 1.21 | 1.13 | 1.27 | 1.51 | 1.50 | 0.96 | 0.98 | 1.00 | 1.47 |
| Bn_25 | 3.73 | 2.13 | 0.37 | 2.48 | 1.33 | 1.81 | 1.20 | 2.02 | 1.02 | 0.47 | 0.51 | 2.81 |
| Bn_26 | 3.96 | 1.29 | 0.33 | 2.59 | 3.65 | 3.39 | 2.59 | 3.10 | 1.18 | 1.06 | 0.89 | 3.04 |
| Bn_27 | 1.50 | 1.32 | 0.82 | 1.50 | 1.98 | 3.07 | 0.62 | 0.53 | 0.49 | 0.64 | 0.87 | 1.95 |
| Bn_28 | 1.56 | 0.79 | 0.14 | 1.14 | 1.32 | 1.37 | 1.26 | 1.23 | 1.15 | 0.92 | 1.14 | 1.29 |
| Bn_29 | 1.88 | 1.59 | 0.16 | 1.58 | 1.98 | 1.63 | 1.55 | 1.79 | 1.69 | 0.76 | 1.16 | 1.71 |
| Bn_03 | 1.13 | 0.89 | 0.29 | 0.65 | 0.91 | 1.04 | 0.71 | 0.64 | 0.80 | 0.87 | 0.63 | 0.82 |
| Bn_30 | 1.16 | 0.76 | 0.12 | 0.97 | 0.98 | 1.09 | 0.97 | 1.05 | 1.05 | 0.97 | 1.04 | 0.95 |
| Bn_31 | 2.01 | 1.72 | 0.29 | 0.92 | 1.77 | 1.10 | 0.82 | 0.85 | 1.23 | 1.14 | 1.03 | 1.74 |
| Bn_32 | 4.82 | 1.62 | 0.66 | 3.61 | 4.37 | 1.95 | 3.70 | 3.09 | 0.82 | 0.78 | 0.95 | 4.08 |
| Bn_33 | 5.25 | 2.30 | 0.54 | 4.00 | 3.80 | 2.49 | 2.47 | 3.09 | 2.07 | 0.89 | 1.80 | 4.14 |
| Bn_34 | 3.02 | 1.53 | 0.32 | 2.21 | 2.33 | 2.29 | 1.83 | 1.98 | 0.78 | 0.90 | 1.52 | 2.43 |
| Bn_36 | 2.46 | 3.95 | 0.84 | 3.47 | 3.77 | 2.31 | 0.78 | 3.72 | 2.59 | 2.86 | 3.04 | 2.66 |
| Bn_39 | 1.49 | 0.84 | 0.19 | 1.50 | 1.81 | 1.30 | 1.61 | 0.85 | 1.47 | 0.98 | 1.27 | 1.50 |
| Bn_04 | 1.37 | 0.79 | 0.15 | 0.97 | 1.16 | 1.43 | 1.15 | 1.14 | 1.26 | 1.10 | 1.19 | 0.93 |
| Bn_41 | 2.68 | 1.03 | 0.24 | 1.77 | 1.74 | 1.82 | 1.55 | 1.54 | 1.14 | 1.17 | 0.93 | 1.91 |
| Bn_43 | 1.74 | 0.71 | 0.16 | 1.30 | 0.79 | 1.79 | 1.05 | 1.52 | 1.77 | 1.60 | 1.36 | 1.57 |
| Bn_44 | 3.89 | 1.62 | 1.03 | 3.04 | 2.68 | 3.93 | 2.36 | 2.93 | 1.63 | 0.74 | 2.62 | 3.57 |
| Bn_46 | 1.06 | 0.60 | 0.15 | 0.83 | 0.86 | 0.73 | 0.96 | 0.76 | 0.81 | 0.80 | 1.02 | 0.87 |
| Bn_47 | 2.60 | 1.94 | 0.23 | 2.19 | 1.82 | 2.04 | 2.00 | 2.03 | 1.54 | 0.84 | 1.16 | 2.41 |
| Bn_49 | 3.89 | 1.32 | 0.64 | 5.98 | 4.99 | 2.47 | 2.64 | 5.35 | 0.97 | 1.03 | 1.31 | 1.76 |
| Bn_05 | 1.81 | 1.50 | 0.38 | 0.99 | 1.96 | 1.61 | 1.23 | 1.38 | 1.33 | 1.24 | 1.15 | 1.35 |
| Bn_50 | 1.84 | 1.72 | 0.98 | 0.76 | 1.47 | 1.53 | 0.94 | 1.03 | 0.65 | 0.67 | 0.82 | 1.68 |
| Bn_52 | 1.95 | 1.36 | 1.10 | 0.84 | 1.31 | 1.34 | 0.53 | 0.64 | 0.63 | 0.70 | 0.75 | 1.75 |
| Bn_53 | 1.71 | 0.92 | 1.53 | 1.09 | 1.46 | 1.33 | 0.94 | 0.89 | 0.69 | 0.70 | 0.74 | 1.64 |
| Bn_54 | 1.50 | 0.92 | 0.27 | 0.86 | 0.81 | 1.60 | 1.30 | 1.37 | 1.22 | 1.12 | 1.57 | 1.23 |
| Bn_56 | 1.34 | 1.47 | 0.99 | 0.54 | 1.51 | 1.06 | 0.76 | 0.80 | 0.77 | 0.61 | 0.69 | 1.13 |
| Bn_59 | 1.85 | 0.80 | 0.17 | 1.47 | 0.86 | 1.86 | 1.57 | 1.63 | 1.64 | 1.38 | 1.09 | 1.70 |
| Bn_06 | 1.77 | 1.68 | 0.66 | 0.43 | 2.13 | 1.80 | 0.52 | 0.38 | 0.47 | 0.52 | 0.54 | 1.33 |
| Bn_60 | 3.43 | 1.27 | 0.27 | 2.77 | 2.77 | 2.24 | 2.42 | 2.13 | 0.99 | 1.04 | 1.22 | 2.86 |
| Bn_62 | 4.96 | 1.86 | 0.45 | 3.82 | 4.37 | 3.00 | 2.58 | 3.40 | 1.53 | 1.04 | 1.45 | 3.91 |
| Bn_63 | 1.74 | 0.71 | 0.23 | 0.24 | 0.60 | 1.41 | 0.57 | 0.84 | 0.80 | 1.10 | 1.01 | 1.19 |
| Bn_64 | 1.37 | 0.81 | 0.13 | 1.00 | 1.27 | 1.09 | 0.97 | 0.98 | 1.04 | 0.75 | 1.09 | 1.10 |
| Bn_67 | 1.49 | 1.18 | 0.18 | 2.05 | 0.23 | 1.16 | 0.74 | 1.84 | 1.28 | 1.40 | 1.09 | 1.48 |
| Bn_68 | 3.32 | 0.85 | 0.27 | 2.40 | 2.80 | 2.41 | 2.29 | 2.40 | 0.79 | 0.80 | 1.06 | 2.08 |
| Bn_07 | 2.08 | 1.06 | 0.90 | 0.64 | 1.05 | 1.70 | 0.65 | 0.68 | 0.70 | 0.68 | 0.72 | 1.61 |
| Bn_08 | 1.57 | 1.28 | 0.18 | 0.84 | 1.20 | 1.27 | 1.07 | 0.61 | 0.95 | 0.80 | 0.89 | 1.32 |
| Bn_09 | 1.23 | 0.81 | 0.11 | 1.01 | 1.15 | 1.27 | 1.06 | 1.05 | 1.24 | 1.18 | 1.12 | 1.00 |
| Fg | 2.21 | 1.34 | 0.66 | 1.91 | 1.29 | 1.51 | 0.88 | 1.95 | 1.08 | 0.60 | 1.86 | 1.58 |
| Rs | 0.70 | 0.61 | 0.28 | 0.89 | 0.60 | 0.75 | 0.74 | 0.88 | 0.96 | 0.81 | 0.86 | 0.74 |
| Ta_01 | 1.43 | 1.06 | 0.28 | 1.11 | 0.90 | 1.33 | 1.06 | 1.23 | 1.30 | 1.20 | 1.14 | 1.14 |
| Ta_10 | 4.13 | 1.76 | 0.57 | 2.15 | 3.10 | 2.69 | 1.43 | 2.31 | 1.08 | 0.74 | 1.44 | 3.00 |
| Ta_11 | 2.94 | 1.59 | 0.67 | 0.73 | 2.96 | 2.55 | 0.79 | 0.52 | 0.70 | 0.64 | 0.69 | 2.34 |
| Ta_12 | 2.63 | 2.63 | 0.52 | 1.55 | 2.00 | 1.66 | 0.86 | 2.37 | 2.11 | 1.92 | 1.65 | 2.27 |
| Ta_13 | 5.43 | 2.32 | 0.49 | 4.06 | 4.36 | 3.34 | 2.87 | 3.38 | 2.04 | 1.17 | 1.88 | 4.21 |
| Ta_14 | 0.90 | 0.47 | 0.11 | 0.58 | 0.26 | 0.83 | 0.80 | 0.81 | 0.92 | 0.60 | 1.12 | 0.69 |
| Ta_15 | 2.37 | 0.66 | 0.22 | 1.56 | 1.40 | 1.62 | 1.99 | 1.86 | 1.72 | 1.82 | 1.43 | 2.21 |
| Ta_16 | 1.69 | 1.01 | 0.13 | 1.50 | 1.67 | 1.58 | 1.46 | 1.42 | 1.10 | 1.12 | 1.11 | 1.51 |
| Ta_18 | 2.63 | 0.93 | 0.45 | 3.45 | 3.31 | 2.79 | 2.46 | 3.44 | 0.65 | 0.62 | 0.90 | 1.91 |
| Ta_19 | 3.27 | 1.18 | 0.35 | 1.79 | 2.41 | 2.04 | 2.37 | 1.69 | 1.19 | 0.93 | 0.88 | 2.06 |
| Ta_02 | 3.89 | 0.55 | 0.46 | 2.15 | 0.69 | 2.36 | 4.25 | 2.14 | 2.78 | 1.39 | 0.61 | 0.66 |
| Ta_20 | 1.91 | 1.14 | 0.19 | 1.29 | 1.65 | 1.79 | 1.70 | 0.98 | 1.16 | 0.94 | 1.17 | 1.63 |
| Ta_21 | 1.75 | 1.20 | 0.19 | 0.73 | 1.33 | 1.12 | 0.69 | 1.59 | 1.36 | 1.40 | 1.17 | 1.47 |
| Ta_22 | 1.30 | 0.68 | 0.12 | 1.07 | 1.19 | 1.19 | 1.26 | 1.18 | 0.86 | 0.82 | 0.91 | 1.18 |
| Ta_23 | 2.02 | 0.86 | 0.18 | 1.43 | 0.94 | 1.85 | 1.64 | 1.53 | 1.75 | 1.70 | 1.55 | 1.72 |
| Ta_24 | 7.86 | 0.85 | 0.60 | 1.90 | 3.01 | 4.40 | 2.74 | 2.99 | 0.85 | 1.84 | 1.11 | 5.00 |
| Ta_25 | 1.06 | 0.57 | 0.13 | 0.73 | 0.70 | 1.00 | 0.99 | 0.79 | 0.96 | 0.92 | 0.90 | 1.04 |
| Ta_26 | 1.34 | 1.01 | 0.12 | 1.05 | 1.18 | 1.20 | 1.20 | 1.20 | 0.91 | 0.87 | 0.94 | 1.16 |
| Ta_27 | 1.92 | 1.20 | 1.06 | 1.04 | 1.38 | 1.65 | 1.02 | 1.15 | 0.67 | 0.67 | 1.17 | 1.73 |
| Ta_28 | 1.87 | 1.05 | 0.95 | 0.87 | 1.44 | 1.94 | 0.82 | 0.93 | 0.73 | 0.73 | 0.87 | 1.22 |
| Ta_29 | 1.60 | 0.94 | 0.21 | 1.00 | 1.18 | 1.21 | 1.08 | 0.67 | 0.77 | 0.63 | 1.12 | 1.30 |
| Ta_03 | 1.28 | 0.84 | 0.13 | 0.98 | 0.53 | 0.87 | 0.83 | 0.76 | 0.66 | 0.64 | 0.69 | 0.87 |
| Ta_30 | 1.55 | 0.57 | 0.12 | 0.95 | 1.35 | 1.19 | 1.42 | 1.05 | 0.98 | 0.79 | 0.98 | 1.28 |
| Ta_31 | 2.16 | 1.08 | 0.22 | 1.02 | 1.79 | 1.93 | 1.54 | 1.54 | 1.21 | 0.73 | 1.61 | 1.66 |
| Ta_32 | 1.90 | 0.91 | 0.16 | 1.44 | 1.76 | 1.63 | 1.52 | 1.55 | 1.17 | 1.15 | 1.17 | 1.62 |
| Ta_33 | 1.61 | 0.81 | 0.15 | 1.15 | 1.52 | 1.48 | 1.33 | 1.38 | 1.07 | 1.02 | 1.09 | 1.36 |
| Ta_34 | 1.48 | 0.88 | 0.11 | 1.07 | 1.34 | 1.26 | 1.29 | 1.21 | 1.05 | 1.02 | 1.07 | 1.27 |
| Ta_35 | 1.67 | 0.98 | 0.14 | 1.14 | 1.47 | 1.33 | 1.23 | 1.30 | 0.97 | 0.96 | 1.09 | 1.28 |
| Ta_36 | 1.29 | 0.78 | 0.09 | 1.21 | 1.29 | 1.22 | 1.19 | 1.20 | 0.91 | 0.80 | 0.97 | 1.23 |
| Ta_37 | 6.12 | 1.79 | 0.46 | 2.10 | 1.52 | 1.76 | 2.03 | 2.88 | 3.46 | 5.13 | 3.38 | 4.37 |
| Ta_38 | 1.01 | 0.60 | 0.14 | 0.53 | 1.03 | 1.22 | 1.02 | 0.48 | 1.12 | 0.79 | 0.49 | 0.55 |
| Ta_39 | 2.39 | 1.09 | 0.33 | 3.04 | 3.12 | 3.27 | 2.47 | 3.01 | 0.82 | 0.61 | 1.18 | 1.65 |
| Ta_04 | 1.54 | 0.79 | 0.15 | 1.29 | 1.40 | 1.40 | 1.31 | 1.28 | 1.00 | 0.92 | 1.03 | 1.38 |
| Ta_40 | 1.52 | 0.55 | 0.34 | 1.10 | 1.11 | 1.52 | 1.08 | 0.96 | 0.70 | 0.79 | 0.94 | 1.15 |
| Ta_41 | 3.13 | 1.66 | 0.30 | 2.30 | 1.16 | 2.97 | 2.80 | 2.68 | 3.15 | 3.11 | 2.05 | 2.96 |
| Ta_05 | 1.76 | 0.87 | 0.14 | 1.34 | 1.49 | 1.57 | 1.52 | 1.39 | 1.22 | 1.04 | 1.13 | 1.40 |
| Ta_06 | 1.54 | 1.01 | 0.40 | 1.12 | 0.67 | 1.62 | 1.68 | 1.48 | 1.36 | 1.53 | 1.51 | 1.65 |
| Ta_07 | 1.95 | 0.76 | 0.14 | 1.51 | 1.77 | 1.63 | 1.44 | 1.47 | 1.23 | 0.80 | 1.16 | 1.72 |
| Ta_08 | 1.10 | 1.08 | 0.21 | 0.61 | 1.28 | 0.50 | 0.56 | 0.85 | 0.82 | 1.08 | 0.76 | 0.90 |
| Ta_09 | 5.60 | 2.50 | 0.57 | 4.44 | 4.95 | 3.69 | 2.57 | 3.70 | 2.18 | 1.19 | 1.80 | 4.48 |

| Isolates | Maltotriose | D-Mannitol | D-Mannose | D-Melezitose | D-Melibiose | α-Methyl-D-Galactoside | β-Methyl-D-Galactoside | α-Methyl-D-Glucoside | β-Methyl-D-Glucoside | Palatinose | D-Psicose_D-Fructose |
| --- | --- | --- | --- | --- | --- | --- | --- | --- | --- | --- | --- |
| Bn_01 | 1.25 | 1.23 | 1.20 | 0.97 | 1.04 | 0.90 | 0.98 | 0.80 | 1.07 | 0.92 | 1.05 |
| Bn_10 | 1.78 | 1.51 | 1.42 | 1.16 | 1.39 | 1.14 | 0.84 | 0.93 | 1.18 | 0.98 | 0.97 |
| Bn_11 | 2.02 | 1.44 | 1.77 | 1.43 | 1.64 | 1.10 | 1.25 | 1.35 | 1.80 | 1.36 | 1.11 |
| Bn_13 | 1.44 | 0.43 | 0.35 | 0.74 | 0.37 | 0.48 | 0.63 | 0.49 | 0.36 | 0.49 | 0.41 |
| Bn_14 | 1.35 | 0.97 | 1.06 | 1.41 | 0.94 | 1.16 | 1.17 | 1.01 | 1.25 | 1.38 | 0.84 |
| Bn_15 | 2.17 | 1.93 | 1.84 | 2.22 | 2.31 | 1.39 | 2.00 | 1.03 | 2.26 | 2.21 | 1.49 |
| Bn_16 | 1.13 | 1.26 | 1.00 | 1.07 | 1.19 | 0.87 | 0.81 | 0.95 | 1.06 | 0.99 | 0.96 |
| Bn_17 | 4.28 | 3.28 | 3.47 | 1.57 | 1.11 | 0.40 | 0.44 | 1.25 | 1.30 | 1.58 | 0.82 |
| Bn_18 | 2.72 | 2.77 | 2.18 | 2.50 | 0.63 | 0.62 | 0.83 | 0.65 | 2.23 | 0.76 | 1.35 |
| Bn_19 | 2.12 | 0.82 | 1.80 | 1.38 | 0.98 | 0.79 | 0.67 | 0.55 | 1.27 | 0.79 | 0.79 |
| Bn_21 | 2.64 | 1.46 | 2.14 | 2.10 | 0.58 | 1.28 | 0.87 | 1.19 | 1.08 | 0.58 | 1.22 |
| Bn_22 | 2.36 | 1.43 | 2.04 | 2.08 | 1.21 | 0.56 | 1.21 | 0.79 | 1.57 | 1.30 | 0.67 |
| Bn_23 | 1.29 | 1.38 | 1.30 | 1.30 | 1.27 | 0.90 | 1.39 | 0.83 | 0.83 | 0.93 | 1.43 |
| Bn_24 | 1.62 | 1.51 | 1.49 | 1.60 | 1.68 | 1.30 | 1.34 | 0.84 | 1.36 | 1.55 | 1.16 |
| Bn_25 | 3.29 | 1.95 | 2.66 | 0.62 | 1.34 | 0.38 | 0.63 | 0.69 | 0.62 | 1.47 | 1.40 |
| Bn_26 | 3.77 | 3.34 | 3.13 | 3.20 | 2.92 | 1.82 | 0.85 | 0.87 | 3.07 | 2.09 | 1.45 |
| Bn_27 | 2.15 | 0.94 | 1.92 | 1.39 | 0.59 | 1.24 | 1.37 | 0.55 | 0.47 | 0.60 | 0.43 |
| Bn_28 | 1.59 | 1.37 | 1.53 | 1.27 | 1.04 | 0.90 | 1.27 | 1.14 | 1.42 | 1.11 | 1.33 |
| Bn_29 | 1.91 | 1.83 | 1.70 | 1.38 | 1.59 | 0.79 | 1.16 | 1.31 | 1.73 | 1.27 | 0.76 |
| Bn_03 | 1.19 | 1.17 | 1.00 | 0.68 | 0.97 | 0.91 | 0.77 | 0.84 | 0.66 | 0.91 | 0.49 |
| Bn_30 | 1.10 | 1.10 | 1.09 | 1.02 | 1.03 | 0.94 | 0.96 | 0.96 | 1.03 | 1.04 | 0.99 |
| Bn_31 | 2.02 | 1.41 | 1.51 | 1.62 | 0.70 | 0.43 | 0.91 | 0.74 | 1.69 | 1.59 | 0.68 |
| Bn_32 | 5.08 | 4.57 | 4.09 | 2.95 | 3.71 | 1.24 | 0.72 | 0.55 | 2.07 | 2.27 | 0.93 |
| Bn_33 | 5.18 | 4.18 | 3.81 | 2.04 | 1.28 | 0.49 | 0.61 | 1.70 | 1.87 | 2.12 | 0.96 |
| Bn_34 | 2.75 | 1.54 | 2.35 | 2.56 | 2.17 | 1.22 | 0.48 | 1.38 | 1.84 | 1.53 | 0.77 |
| Bn_36 | 2.48 | 2.84 | 2.81 | 2.41 | 2.86 | 3.26 | 3.15 | 3.18 | 2.84 | 2.75 | 3.32 |
| Bn_39 | 1.24 | 1.64 | 1.38 | 1.37 | 1.53 | 1.44 | 1.04 | 1.04 | 1.31 | 1.27 | 0.71 |
| Bn_04 | 1.33 | 1.27 | 1.30 | 1.30 | 1.21 | 1.12 | 1.14 | 1.11 | 1.29 | 1.22 | 1.02 |
| Bn_41 | 2.31 | 1.55 | 2.12 | 1.76 | 1.78 | 0.81 | 0.84 | 0.73 | 1.51 | 1.10 | 0.73 |
| Bn_43 | 1.65 | 1.26 | 1.45 | 1.63 | 1.73 | 1.62 | 1.60 | 1.07 | 1.68 | 1.53 | 1.18 |
| Bn_44 | 3.78 | 4.37 | 3.21 | 2.07 | 3.15 | 1.20 | 0.62 | 1.07 | 3.43 | 2.75 | 1.79 |
| Bn_46 | 1.06 | 0.78 | 0.91 | 1.00 | 0.98 | 0.95 | 0.55 | 0.87 | 0.80 | 1.00 | 0.61 |
| Bn_47 | 2.69 | 2.50 | 2.82 | 1.57 | 2.24 | 0.91 | 0.61 | 0.83 | 2.33 | 2.38 | 1.66 |
| Bn_49 | 1.23 | 2.82 | 3.42 | 3.30 | 0.74 | 1.14 | 1.00 | 0.85 | 0.71 | 0.90 | 1.70 |
| Bn_05 | 1.62 | 1.45 | 1.60 | 1.19 | 1.18 | 1.03 | 0.96 | 1.17 | 1.28 | 1.15 | 0.91 |
| Bn_50 | 1.88 | 0.74 | 1.65 | 1.37 | 0.96 | 0.76 | 0.63 | 0.53 | 1.19 | 0.80 | 1.08 |
| Bn_52 | 1.90 | 0.71 | 1.60 | 1.27 | 0.84 | 0.72 | 0.61 | 0.49 | 1.23 | 0.71 | 0.83 |
| Bn_53 | 1.75 | 0.74 | 1.42 | 1.21 | 0.96 | 0.81 | 0.75 | 0.89 | 1.14 | 0.70 | 1.29 |
| Bn_54 | 1.34 | 1.30 | 0.69 | 1.11 | 1.37 | 1.06 | 1.43 | 1.29 | 1.26 | 1.15 | 1.08 |
| Bn_56 | 1.36 | 1.07 | 1.14 | 1.16 | 0.99 | 0.87 | 0.79 | 0.73 | 0.92 | 0.86 | 0.90 |
| Bn_59 | 1.84 | 1.51 | 1.68 | 1.73 | 1.91 | 1.67 | 0.96 | 1.05 | 1.96 | 1.63 | 1.33 |
| Bn_06 | 1.96 | 0.89 | 1.00 | 0.99 | 0.57 | 0.65 | 0.56 | 0.56 | 1.35 | 1.09 | 0.55 |
| Bn_60 | 3.38 | 1.78 | 2.80 | 3.03 | 2.71 | 0.81 | 0.74 | 1.11 | 2.45 | 1.88 | 0.89 |
| Bn_62 | 5.23 | 4.05 | 3.93 | 1.68 | 1.63 | 0.51 | 0.71 | 1.31 | 1.67 | 1.77 | 0.93 |
| Bn_63 | 1.58 | 1.10 | 1.24 | 1.32 | 1.06 | 0.99 | 0.94 | 0.82 | 1.12 | 1.05 | 0.96 |
| Bn_64 | 1.27 | 1.00 | 1.16 | 1.18 | 1.29 | 1.01 | 0.98 | 1.12 | 1.31 | 1.16 | 0.69 |
| Bn_67 | 1.78 | 1.18 | 1.53 | 1.05 | 1.56 | 0.61 | 0.74 | 1.20 | 1.39 | 1.20 | 0.76 |
| Bn_68 | 3.25 | 2.53 | 2.64 | 2.73 | 1.90 | 1.07 | 0.86 | 1.11 | 2.52 | 1.35 | 1.24 |
| Bn_07 | 1.90 | 0.76 | 1.69 | 1.36 | 0.78 | 0.78 | 0.67 | 0.52 | 1.07 | 0.82 | 0.77 |
| Bn_08 | 1.41 | 1.70 | 1.55 | 0.81 | 1.01 | 0.98 | 1.06 | 0.80 | 0.99 | 0.80 | 0.96 |
| Bn_09 | 1.22 | 1.19 | 1.20 | 1.14 | 1.16 | 0.97 | 1.12 | 1.04 | 1.11 | 1.10 | 1.07 |
| Fg | 1.56 | 1.33 | 2.02 | 2.53 | 0.21 | 0.71 | 0.79 | 0.48 | 1.91 | 1.10 | 0.99 |
| Rs | 0.83 | 0.83 | 0.69 | 0.89 | 0.90 | 0.51 | 0.72 | 0.73 | 0.75 | 1.07 | 0.93 |
| Ta_01 | 1.43 | 1.22 | 1.27 | 1.13 | 1.30 | 0.95 | 1.05 | 1.14 | 1.13 | 1.17 | 1.20 |
| Ta_10 | 3.07 | 2.14 | 3.13 | 1.36 | 1.45 | 0.52 | 0.67 | 1.20 | 1.13 | 1.25 | 0.85 |
| Ta_11 | 2.48 | 0.92 | 2.44 | 0.89 | 0.80 | 0.79 | 0.68 | 0.67 | 0.70 | 0.63 | 0.84 |
| Ta_12 | 2.65 | 1.61 | 2.39 | 2.22 | 2.46 | 1.85 | 1.37 | 1.59 | 2.04 | 1.89 | 1.21 |
| Ta_13 | 5.40 | 4.57 | 4.44 | 2.22 | 1.81 | 0.53 | 0.75 | 1.88 | 1.98 | 2.26 | 1.05 |
| Ta_14 | 0.89 | 1.11 | 0.67 | 1.07 | 1.04 | 0.55 | 0.52 | 0.73 | 1.32 | 0.73 | 0.68 |
| Ta_15 | 2.54 | 2.20 | 1.73 | 2.21 | 2.30 | 1.29 | 1.98 | 1.02 | 2.14 | 1.80 | 1.62 |
| Ta_16 | 1.69 | 1.57 | 1.62 | 1.45 | 1.30 | 0.93 | 1.09 | 1.11 | 1.22 | 1.28 | 1.12 |
| Ta_18 | 2.33 | 2.51 | 2.24 | 2.41 | 0.67 | 0.68 | 0.83 | 0.64 | 2.06 | 0.76 | 1.36 |
| Ta_19 | 2.94 | 2.61 | 2.98 | 3.01 | 2.33 | 1.07 | 1.24 | 0.92 | 2.87 | 2.92 | 1.69 |
| Ta_02 | 2.77 | 1.17 | 1.64 | 1.14 | 1.83 | 1.36 | 2.02 | 3.49 | 1.50 | 2.44 | 1.48 |
| Ta_20 | 1.89 | 1.79 | 1.79 | 1.56 | 1.46 | 1.02 | 0.93 | 1.24 | 1.72 | 1.62 | 1.09 |
| Ta_21 | 1.69 | 1.36 | 1.51 | 0.95 | 1.32 | 1.02 | 0.72 | 0.75 | 1.50 | 1.12 | 0.71 |
| Ta_22 | 1.26 | 1.27 | 1.27 | 1.05 | 1.11 | 0.81 | 0.85 | 0.83 | 1.22 | 1.09 | 0.92 |
| Ta_23 | 1.78 | 1.47 | 1.48 | 1.81 | 1.82 | 1.81 | 1.66 | 1.12 | 1.71 | 1.64 | 1.11 |
| Ta_24 | 6.18 | 3.70 | 4.75 | 3.93 | 1.99 | 1.63 | 2.47 | 0.74 | 3.98 | 2.78 | 2.59 |
| Ta_25 | 1.49 | 1.00 | 0.70 | 1.23 | 1.21 | 0.87 | 1.00 | 0.85 | 0.92 | 1.18 | 1.11 |
| Ta_26 | 1.37 | 1.30 | 1.28 | 1.14 | 1.06 | 0.99 | 0.95 | 1.03 | 1.18 | 1.10 | 1.00 |
| Ta_27 | 1.75 | 1.23 | 1.71 | 1.64 | 1.04 | 0.81 | 0.67 | 0.78 | 1.30 | 0.83 | 1.26 |
| Ta_28 | 1.70 | 1.05 | 1.65 | 1.24 | 0.95 | 0.78 | 0.89 | 1.23 | 1.28 | 1.02 | 1.16 |
| Ta_29 | 1.43 | 1.51 | 1.32 | 1.41 | 1.01 | 0.89 | 0.84 | 0.91 | 1.09 | 1.40 | 1.06 |
| Ta_03 | 1.03 | 0.25 | 0.70 | 1.19 | 0.82 | 0.72 | 0.82 | 0.65 | 0.95 | 0.89 | 0.38 |
| Ta_30 | 1.56 | 1.16 | 1.41 | 1.02 | 0.97 | 1.03 | 0.85 | 0.96 | 1.34 | 1.15 | 0.99 |
| Ta_31 | 2.02 | 1.81 | 1.92 | 1.01 | 1.33 | 1.55 | 1.02 | 1.11 | 1.52 | 1.69 | 1.02 |
| Ta_32 | 1.85 | 1.65 | 1.78 | 1.54 | 1.47 | 1.01 | 1.25 | 1.24 | 1.42 | 1.50 | 1.15 |
| Ta_33 | 1.58 | 1.35 | 1.54 | 1.44 | 1.27 | 0.94 | 1.10 | 1.10 | 1.44 | 1.29 | 1.16 |
| Ta_34 | 1.46 | 1.19 | 1.38 | 1.21 | 1.05 | 0.82 | 0.88 | 1.03 | 1.28 | 1.20 | 1.15 |
| Ta_35 | 1.53 | 1.27 | 1.51 | 1.33 | 1.10 | 0.90 | 1.00 | 0.97 | 1.15 | 1.22 | 1.18 |
| Ta_36 | 1.30 | 1.16 | 1.28 | 1.06 | 1.06 | 0.84 | 0.81 | 0.79 | 1.11 | 1.04 | 0.75 |
| Ta_37 | 5.69 | 3.00 | 2.38 | 3.66 | 4.21 | 2.89 | 3.43 | 0.61 | 5.08 | 4.87 | 2.33 |
| Ta_38 | 1.05 | 0.88 | 0.90 | 1.28 | 0.91 | 0.38 | 0.49 | 0.67 | 0.83 | 0.86 | 1.41 |
| Ta_39 | 1.98 | 2.43 | 2.30 | 2.06 | 1.22 | 0.71 | 1.06 | 0.60 | 2.07 | 0.61 | 1.51 |
| Ta_04 | 1.52 | 1.37 | 1.46 | 1.38 | 1.24 | 1.00 | 0.98 | 1.13 | 1.09 | 1.23 | 1.02 |
| Ta_40 | 1.40 | 1.41 | 1.31 | 1.43 | 1.23 | 1.16 | 1.06 | 0.92 | 1.22 | 1.31 | 0.89 |
| Ta_41 | 3.22 | 2.34 | 2.79 | 3.25 | 3.28 | 2.99 | 2.94 | 1.61 | 3.15 | 2.92 | 2.74 |
| Ta_05 | 1.76 | 1.34 | 1.68 | 1.36 | 1.23 | 0.79 | 1.15 | 1.16 | 1.55 | 1.22 | 1.40 |
| Ta_06 | 1.83 | 1.47 | 1.71 | 1.52 | 1.37 | 1.23 | 1.00 | 1.74 | 1.24 | 1.58 | 1.04 |
| Ta_07 | 1.92 | 1.51 | 1.78 | 1.36 | 1.36 | 0.64 | 0.81 | 1.09 | 1.64 | 1.12 | 1.04 |
| Ta_08 | 0.96 | 0.89 | 1.21 | 0.82 | 0.71 | 0.78 | 0.74 | 0.66 | 0.92 | 0.89 | 0.58 |
| Ta_09 | 5.44 | 4.92 | 4.79 | 2.29 | 2.41 | 0.56 | 0.79 | 1.86 | 2.06 | 2.61 | 1.03 |

| Isolates | D-Raffinose | L-Rhamnose | D-Ribose | Salicin | Sedoheptulosan | D-Sorbitol | L-Sorbose | Stachyose | Sucrose | D-Tagatose | D-Trehalose | Turanose |
| --- | --- | --- | --- | --- | --- | --- | --- | --- | --- | --- | --- | --- |
| Bn_01 | 1.06 | 0.87 | 0.65 | 0.97 | 1.08 | 1.07 | 0.36 | 1.14 | 1.23 | 0.94 | 1.17 | 1.04 |
| Bn_10 | 1.29 | 0.77 | 1.26 | 1.15 | 1.47 | 1.18 | 0.75 | 1.33 | 1.79 | 1.03 | 1.38 | 1.10 |
| Bn_11 | 1.76 | 1.36 | 1.48 | 1.59 | 1.01 | 2.03 | 1.99 | 1.65 | 2.05 | 1.31 | 1.29 | 1.77 |
| Bn_13 | 0.61 | 0.70 | 1.56 | 0.60 | 0.44 | 0.58 | 0.54 | 0.49 | 0.59 | 0.45 | 0.71 | 0.83 |
| Bn_14 | 1.10 | 1.27 | 0.61 | 0.87 | 0.89 | 0.88 | 0.15 | 1.16 | 1.47 | 0.42 | 0.91 | 1.31 |
| Bn_15 | 2.41 | 2.06 | 1.69 | 2.05 | 1.98 | 1.99 | 0.67 | 2.05 | 2.37 | 0.28 | 2.13 | 2.31 |
| Bn_16 | 1.20 | 1.05 | 1.18 | 0.82 | 0.99 | 1.18 | 0.91 | 0.97 | 1.25 | 0.48 | 1.03 | 1.18 |
| Bn_17 | 2.78 | 2.26 | 4.33 | 1.39 | 2.12 | 3.41 | 3.24 | 1.19 | 3.45 | 0.42 | 1.78 | 3.22 |
| Bn_18 | 1.93 | 2.63 | 2.20 | 2.88 | 2.51 | 2.13 | 1.88 | 1.85 | 2.48 | 0.42 | 3.14 | 0.96 |
| Bn_19 | 0.78 | 0.70 | 2.32 | 1.02 | 1.55 | 1.56 | 1.07 | 0.98 | 0.76 | 0.77 | 1.80 | 0.93 |
| Bn_21 | 1.08 | 0.85 | 1.04 | 0.85 | 1.55 | 1.34 | 0.66 | 0.70 | 0.72 | 0.79 | 1.03 | 1.65 |
| Bn_22 | 1.85 | 1.90 | 2.58 | 1.21 | 1.16 | 2.47 | 1.82 | 1.42 | 2.14 | 0.47 | 1.68 | 2.14 |
| Bn_23 | 0.95 | 0.95 | 0.85 | 1.06 | 0.60 | 0.98 | 0.13 | 1.05 | 1.51 | 0.59 | 1.34 | 1.73 |
| Bn_24 | 1.53 | 1.23 | 1.36 | 1.10 | 1.27 | 1.73 | 1.26 | 1.44 | 1.52 | 0.90 | 1.49 | 1.46 |
| Bn_25 | 1.99 | 1.17 | 3.86 | 0.63 | 1.67 | 0.71 | 0.32 | 0.91 | 2.58 | 0.32 | 0.86 | 2.19 |
| Bn_26 | 2.78 | 3.29 | 4.01 | 2.39 | 3.06 | 3.04 | 2.86 | 1.83 | 3.16 | 0.99 | 2.77 | 3.00 |
| Bn_27 | 1.09 | 0.91 | 1.13 | 0.58 | 0.89 | 1.27 | 0.99 | 1.20 | 0.74 | 0.44 | 2.92 | 0.88 |
| Bn_28 | 1.27 | 1.35 | 1.11 | 0.99 | 0.62 | 1.56 | 1.35 | 1.17 | 1.55 | 0.90 | 1.33 | 1.39 |
| Bn_29 | 1.72 | 1.28 | 1.52 | 1.48 | 0.71 | 2.02 | 1.91 | 1.58 | 1.89 | 1.07 | 1.40 | 1.68 |
| Bn_03 | 0.86 | 0.63 | 0.67 | 0.71 | 0.59 | 1.02 | 0.90 | 0.71 | 0.89 | 0.71 | 0.91 | 0.87 |
| Bn_30 | 0.96 | 0.93 | 0.58 | 0.91 | 1.08 | 0.96 | 0.75 | 0.96 | 1.03 | 0.90 | 1.09 | 1.00 |
| Bn_31 | 1.59 | 1.21 | 1.69 | 0.84 | 1.17 | 1.50 | 1.27 | 1.22 | 1.34 | 0.55 | 0.77 | 1.82 |
| Bn_32 | 3.93 | 2.22 | 5.04 | 1.30 | 2.17 | 4.14 | 2.81 | 3.85 | 4.33 | 0.59 | 3.39 | 4.02 |
| Bn_33 | 2.77 | 3.29 | 5.27 | 2.23 | 2.43 | 4.70 | 3.82 | 1.87 | 4.07 | 0.57 | 2.50 | 4.08 |
| Bn_34 | 2.26 | 1.71 | 2.89 | 1.69 | 1.42 | 3.12 | 2.38 | 2.05 | 2.84 | 0.57 | 2.30 | 2.47 |
| Bn_36 | 2.71 | 3.10 | 1.64 | 3.04 | 3.39 | 1.70 | 0.37 | 3.51 | 2.69 | 0.65 | 2.90 | 3.05 |
| Bn_39 | 1.39 | 0.93 | 0.76 | 1.54 | 1.08 | 1.70 | 1.42 | 1.46 | 1.37 | 0.55 | 1.18 | 1.48 |
| Bn_04 | 1.12 | 1.13 | 0.75 | 1.02 | 1.36 | 1.22 | 0.45 | 1.11 | 1.31 | 1.05 | 1.29 | 1.14 |
| Bn_41 | 1.90 | 1.35 | 2.13 | 1.19 | 1.03 | 2.23 | 1.96 | 1.62 | 2.29 | 0.72 | 1.65 | 1.71 |
| Bn_43 | 1.61 | 1.65 | 1.11 | 1.11 | 1.16 | 1.82 | 1.56 | 1.83 | 1.70 | 0.30 | 1.43 | 1.67 |
| Bn_44 | 3.55 | 3.21 | 3.53 | 2.32 | 2.47 | 3.92 | 4.07 | 2.53 | 3.80 | 1.26 | 3.59 | 3.30 |
| Bn_46 | 1.01 | 1.03 | 1.05 | 0.60 | 0.89 | 1.01 | 0.30 | 0.90 | 1.11 | 1.03 | 0.91 | 0.69 |
| Bn_47 | 2.62 | 2.11 | 2.55 | 2.08 | 1.67 | 2.56 | 2.34 | 1.93 | 1.75 | 0.94 | 1.84 | 2.38 |
| Bn_49 | 1.14 | 1.06 | 4.23 | 3.02 | 3.24 | 4.39 | 0.82 | 1.04 | 4.31 | 0.88 | 5.20 | 1.08 |
| Bn_05 | 1.28 | 0.91 | 1.33 | 1.41 | 1.79 | 1.06 | 0.67 | 1.28 | 1.47 | 1.12 | 1.37 | 1.33 |
| Bn_50 | 0.92 | 0.69 | 2.26 | 1.04 | 1.44 | 1.40 | 1.08 | 0.82 | 0.78 | 0.80 | 1.85 | 1.50 |
| Bn_52 | 0.82 | 0.68 | 2.15 | 1.03 | 1.52 | 1.22 | 1.31 | 0.90 | 0.97 | 0.90 | 1.80 | 1.31 |
| Bn_53 | 0.78 | 0.64 | 1.81 | 0.90 | 1.29 | 1.34 | 0.95 | 1.01 | 1.12 | 1.32 | 1.47 | 1.28 |
| Bn_54 | 1.33 | 1.20 | 0.83 | 0.94 | 1.08 | 1.58 | 0.14 | 1.39 | 1.36 | 0.43 | 1.20 | 1.61 |
| Bn_56 | 0.78 | 0.64 | 1.52 | 0.73 | 0.99 | 1.19 | 0.70 | 1.04 | 0.86 | 0.79 | 1.23 | 1.26 |
| Bn_59 | 1.87 | 1.33 | 1.42 | 1.14 | 1.00 | 1.64 | 1.55 | 1.89 | 1.83 | 0.35 | 1.39 | 1.76 |
| Bn_06 | 0.93 | 0.78 | 1.58 | 1.41 | 1.56 | 1.36 | 0.64 | 0.82 | 1.15 | 0.61 | 1.94 | 0.67 |
| Bn_60 | 2.68 | 1.88 | 3.19 | 1.52 | 1.40 | 0.98 | 2.80 | 2.02 | 3.10 | 0.47 | 2.66 | 3.19 |
| Bn_62 | 2.66 | 2.68 | 5.37 | 1.72 | 2.17 | 4.47 | 2.66 | 1.37 | 4.46 | 0.50 | 2.08 | 4.24 |
| Bn_63 | 1.26 | 0.97 | 1.13 | 1.41 | 1.08 | 0.97 | 0.18 | 1.26 | 1.32 | 0.98 | 0.71 | 1.05 |
| Bn_64 | 1.27 | 1.04 | 0.72 | 0.96 | 1.23 | 1.27 | 0.74 | 1.29 | 1.29 | 0.38 | 0.97 | 1.23 |
| Bn_67 | 1.57 | 1.01 | 0.91 | 0.81 | 0.67 | 1.16 | 0.31 | 0.89 | 1.10 | 0.26 | 1.45 | 1.63 |
| Bn_68 | 1.76 | 2.40 | 3.12 | 2.04 | 1.89 | 2.86 | 2.63 | 1.10 | 2.68 | 0.89 | 1.91 | 2.26 |
| Bn_07 | 0.78 | 0.67 | 2.18 | 1.10 | 1.38 | 1.16 | 1.23 | 0.81 | 0.79 | 0.76 | 1.61 | 1.34 |
| Bn_08 | 0.84 | 0.84 | 0.63 | 1.33 | 0.99 | 1.64 | 0.11 | 1.00 | 1.08 | 0.45 | 1.52 | 0.89 |
| Bn_09 | 1.09 | 1.09 | 0.85 | 1.02 | 1.13 | 1.13 | 0.68 | 1.06 | 1.12 | 0.91 | 1.26 | 1.08 |
| Fg | 1.21 | 1.01 | 0.98 | 0.61 | 1.39 | 1.48 | 0.16 | 0.91 | 0.61 | 0.48 | 1.16 | 2.00 |
| Rs | 1.15 | 0.98 | 0.68 | 1.17 | 0.57 | 0.78 | 0.51 | 0.99 | 0.71 | 0.49 | 0.80 | 0.80 |
| Ta_01 | 1.12 | 1.10 | 0.86 | 1.06 | 1.23 | 1.12 | 0.57 | 1.08 | 1.35 | 0.89 | 1.40 | 1.30 |
| Ta_10 | 2.19 | 2.16 | 2.28 | 1.13 | 1.85 | 2.14 | 1.81 | 1.26 | 2.27 | 0.56 | 3.17 | 3.00 |
| Ta_11 | 0.86 | 0.82 | 1.01 | 0.80 | 1.01 | 0.88 | 1.05 | 0.88 | 0.88 | 0.49 | 2.86 | 0.90 |
| Ta_12 | 2.34 | 1.77 | 1.37 | 2.38 | 1.34 | 2.57 | 1.19 | 1.74 | 2.59 | 0.68 | 1.24 | 2.30 |
| Ta_13 | 3.19 | 3.70 | 4.05 | 2.18 | 2.69 | 4.59 | 3.66 | 2.16 | 4.68 | 0.70 | 2.96 | 4.49 |
| Ta_14 | 0.89 | 1.02 | 0.96 | 0.86 | 0.87 | 0.77 | 0.45 | 0.68 | 0.97 | 0.41 | 0.51 | 0.63 |
| Ta_15 | 2.51 | 1.45 | 1.81 | 1.50 | 1.65 | 2.27 | 1.30 | 1.60 | 2.32 | 0.74 | 2.21 | 2.08 |
| Ta_16 | 1.50 | 1.38 | 1.25 | 1.02 | 1.15 | 1.61 | 1.58 | 1.32 | 1.68 | 1.08 | 1.39 | 1.49 |
| Ta_18 | 1.89 | 2.53 | 1.52 | 2.47 | 2.67 | 2.09 | 2.02 | 1.63 | 2.39 | 1.34 | 2.62 | 0.93 |
| Ta_19 | 2.34 | 2.05 | 2.74 | 1.32 | 2.07 | 3.05 | 2.67 | 1.83 | 3.03 | 1.44 | 2.29 | 1.38 |
| Ta_02 | 1.58 | 1.71 | 2.64 | 1.69 | 1.89 | 0.60 | 0.34 | 1.89 | 3.68 | 1.74 | 2.02 | 1.93 |
| Ta_20 | 1.68 | 1.62 | 1.35 | 1.26 | 1.19 | 1.85 | 1.77 | 1.21 | 1.81 | 1.01 | 1.65 | 1.74 |
| Ta_21 | 1.33 | 1.00 | 0.76 | 0.60 | 1.06 | 1.24 | 0.79 | 1.27 | 1.46 | 0.39 | 0.96 | 1.35 |
| Ta_22 | 1.23 | 1.02 | 1.05 | 0.98 | 0.93 | 1.32 | 1.27 | 0.94 | 1.30 | 0.86 | 1.11 | 1.23 |
| Ta_23 | 1.83 | 1.67 | 1.40 | 1.04 | 1.26 | 1.94 | 1.45 | 1.78 | 1.77 | 0.27 | 1.65 | 1.86 |
| Ta_24 | 3.61 | 3.91 | 6.11 | 1.58 | 5.01 | 5.01 | 2.75 | 2.00 | 5.04 | 0.89 | 3.35 | 4.34 |
| Ta_25 | 1.16 | 0.97 | 0.62 | 0.79 | 1.01 | 1.11 | 0.23 | 0.92 | 1.20 | 0.69 | 1.35 | 1.16 |
| Ta_26 | 1.22 | 1.20 | 1.02 | 0.98 | 0.82 | 1.24 | 1.19 | 1.08 | 1.35 | 0.92 | 1.17 | 1.22 |
| Ta_27 | 0.87 | 0.85 | 1.96 | 0.98 | 1.42 | 1.85 | 0.88 | 0.93 | 1.15 | 0.86 | 1.53 | 1.76 |
| Ta_28 | 0.92 | 0.90 | 1.77 | 1.10 | 2.11 | 1.08 | 0.97 | 0.94 | 1.15 | 0.99 | 1.70 | 1.19 |
| Ta_29 | 1.04 | 0.93 | 0.77 | 1.14 | 1.18 | 1.34 | 1.47 | 1.07 | 1.45 | 0.79 | 1.11 | 1.44 |
| Ta_03 | 0.97 | 0.93 | 0.51 | 0.42 | 0.86 | 0.53 | 0.12 | 0.94 | 0.91 | 0.13 | 0.72 | 1.08 |
| Ta_30 | 1.36 | 1.15 | 1.07 | 0.97 | 0.48 | 1.55 | 1.38 | 0.88 | 1.53 | 0.70 | 1.05 | 1.38 |
| Ta_31 | 2.18 | 2.00 | 1.98 | 1.20 | 0.83 | 1.28 | 1.36 | 1.70 | 1.36 | 1.66 | 2.08 | 1.87 |
| Ta_32 | 1.66 | 1.54 | 1.45 | 1.20 | 1.42 | 1.69 | 1.64 | 1.34 | 1.85 | 1.06 | 1.39 | 1.62 |
| Ta_33 | 1.43 | 1.31 | 1.29 | 1.19 | 0.78 | 1.52 | 1.39 | 1.07 | 1.61 | 1.11 | 1.26 | 1.43 |
| Ta_34 | 1.34 | 1.22 | 1.16 | 0.95 | 0.65 | 1.38 | 1.14 | 1.08 | 1.42 | 0.86 | 1.15 | 1.29 |
| Ta_35 | 1.36 | 1.30 | 1.28 | 0.93 | 1.27 | 1.56 | 1.40 | 1.03 | 1.60 | 0.89 | 1.18 | 1.39 |
| Ta_36 | 1.16 | 1.00 | 0.94 | 1.03 | 0.88 | 1.29 | 1.21 | 1.04 | 1.27 | 0.85 | 1.12 | 1.14 |
| Ta_37 | 5.18 | 3.35 | 3.35 | 2.25 | 4.36 | 3.41 | 1.87 | 2.92 | 3.77 | 0.83 | 4.76 | 3.83 |
| Ta_38 | 0.88 | 0.57 | 1.10 | 0.93 | 1.24 | 0.78 | 0.13 | 1.01 | 0.44 | 0.20 | 0.82 | 0.83 |
| Ta_39 | 1.58 | 2.11 | 1.63 | 2.21 | 2.52 | 2.44 | 1.57 | 1.70 | 2.16 | 0.75 | 2.22 | 0.77 |
| Ta_04 | 1.35 | 1.32 | 1.12 | 0.90 | 1.19 | 1.58 | 1.37 | 1.17 | 1.59 | 0.93 | 1.21 | 1.36 |
| Ta_40 | 1.27 | 1.10 | 1.44 | 0.95 | 1.03 | 1.11 | 1.15 | 1.38 | 1.23 | 0.48 | 1.19 | 1.19 |
| Ta_41 | 3.10 | 2.69 | 2.37 | 2.26 | 1.71 | 3.11 | 2.91 | 3.17 | 3.21 | 0.83 | 2.47 | 3.20 |
| Ta_05 | 1.56 | 1.49 | 1.17 | 1.07 | 0.51 | 1.59 | 1.34 | 1.23 | 1.73 | 1.13 | 1.31 | 1.62 |
| Ta_06 | 1.63 | 1.21 | 1.33 | 1.59 | 1.24 | 0.66 | 1.42 | 1.42 | 1.51 | 1.08 | 1.66 | 0.89 |
| Ta_07 | 1.55 | 1.39 | 1.38 | 1.47 | 1.24 | 1.90 | 1.75 | 1.00 | 1.84 | 0.81 | 1.42 | 1.61 |
| Ta_08 | 0.90 | 1.06 | 0.74 | 0.65 | 0.55 | 1.02 | 0.77 | 0.86 | 0.92 | 0.29 | 0.66 | 0.88 |
| Ta_09 | 3.44 | 3.53 | 4.38 | 2.11 | 2.93 | 4.40 | 3.69 | 2.30 | 4.93 | 0.67 | 2.72 | 4.62 |

| Isolates | Xylitol | D-Xylose | γ-Amino_  butyric_  acid | Bromo_  succinic-  Acid | Fumaric-  Acid | β-Hydroxy_  butyric-  Acid | Gamma-  Hydoxy  butyric-  Acid | Rho-  Hydroxy_  phenylacetic-  Acid | α-Keto-glutaric-Acid | D-Lactic-Acid_  Methyl_  Ester | L-Lactic-Acid |
| --- | --- | --- | --- | --- | --- | --- | --- | --- | --- | --- | --- |
| Bn_01 | 1.17 | 1.18 | 0.90 | 0.33 | 0.53 | 0.53 | 1.03 | 0.90 | 0.52 | 1.03 | 0.62 |
| Bn_10 | 1.21 | 1.79 | 0.80 | 0.39 | 0.31 | 0.37 | 0.80 | 0.87 | 0.70 | 1.22 | 0.64 |
| Bn_11 | 1.20 | 1.79 | 1.39 | 0.87 | 1.14 | 0.64 | 0.94 | 1.22 | 1.04 | 1.33 | 1.24 |
| Bn_13 | 1.24 | 1.52 | 0.58 | 0.26 | 0.27 | 0.55 | 0.77 | 0.33 | 0.59 | 0.64 | 0.49 |
| Bn_14 | 0.86 | 0.99 | 1.02 | 0.46 | 1.22 | 0.24 | 0.56 | 0.91 | 0.71 | 0.84 | 0.74 |
| Bn_15 | 1.67 | 2.17 | 1.49 | 2.08 | 2.27 | 0.42 | 0.66 | 0.60 | 0.78 | 0.74 | 1.22 |
| Bn_16 | 0.40 | 1.18 | 0.88 | 0.81 | 0.92 | 0.88 | 1.10 | 1.01 | 0.98 | 0.48 | 0.94 |
| Bn_17 | 0.73 | 4.64 | 4.57 | 1.92 | 3.53 | 0.78 | 1.77 | 2.04 | 1.61 | 0.67 | 2.99 |
| Bn_18 | 1.03 | 3.36 | 4.26 | 2.19 | 4.51 | 3.21 | 3.69 | 3.89 | 2.38 | 1.18 | 3.54 |
| Bn_19 | 2.55 | 2.56 | 1.08 | 0.41 | 0.80 | 0.43 | 0.75 | 0.69 | 1.50 | 1.69 | 1.09 |
| Bn_21 | 0.88 | 0.74 | 0.51 | 0.29 | 0.41 | 0.24 | 0.50 | 0.70 | 0.51 | 0.46 | 0.69 |
| Bn_22 | 0.67 | 2.69 | 2.07 | 0.63 | 1.23 | 1.39 | 1.20 | 2.25 | 0.65 | 0.84 | 0.66 |
| Bn_23 | 1.07 | 1.04 | 0.67 | 0.60 | 0.88 | 0.23 | 0.71 | 0.82 | 0.85 | 1.15 | 0.74 |
| Bn_24 | 1.26 | 1.60 | 1.19 | 0.70 | 1.25 | 0.53 | 0.69 | 1.11 | 1.02 | 0.90 | 0.73 |
| Bn_25 | 0.58 | 4.76 | 2.15 | 0.43 | 0.42 | 0.79 | 0.82 | 0.78 | 0.79 | 0.45 | 1.72 |
| Bn_26 | 2.09 | 4.13 | 3.69 | 1.00 | 1.24 | 1.36 | 1.35 | 2.26 | 1.56 | 0.72 | 1.49 |
| Bn_27 | 0.97 | 1.01 | 0.49 | 0.29 | 0.43 | 0.33 | 0.95 | 0.50 | 0.86 | 1.38 | 1.33 |
| Bn_28 | 1.08 | 1.37 | 1.09 | 0.23 | 0.50 | 0.26 | 1.01 | 0.85 | 0.94 | 0.86 | 1.09 |
| Bn_29 | 1.27 | 1.61 | 1.54 | 0.93 | 1.25 | 0.70 | 0.79 | 1.32 | 1.08 | 1.05 | 1.31 |
| Bn_03 | 0.74 | 1.03 | 0.70 | 0.24 | 0.30 | 0.33 | 0.96 | 0.80 | 0.73 | 1.05 | 0.76 |
| Bn_30 | 0.96 | 1.10 | 0.81 | 0.73 | 0.69 | 0.26 | 0.91 | 0.91 | 0.83 | 0.82 | 0.92 |
| Bn_31 | 0.80 | 2.00 | 1.54 | 0.66 | 0.73 | 0.38 | 0.87 | 0.59 | 0.73 | 0.73 | 1.27 |
| Bn_32 | 1.37 | 5.10 | 1.71 | 1.28 | 2.12 | 1.60 | 1.20 | 1.67 | 1.05 | 1.37 | 3.19 |
| Bn_33 | 1.10 | 5.37 | 6.04 | 3.00 | 5.08 | 1.18 | 2.70 | 3.63 | 2.30 | 1.07 | 4.21 |
| Bn_34 | 1.63 | 2.87 | 2.20 | 0.92 | 1.70 | 1.35 | 1.39 | 1.89 | 0.81 | 1.29 | 0.53 |
| Bn_36 | 1.07 | 2.56 | 0.68 | 2.18 | 3.30 | 0.22 | 0.41 | 0.23 | 0.92 | 0.89 | 2.71 |
| Bn_39 | 1.13 | 1.39 | 1.48 | 0.71 | 1.53 | 0.78 | 1.56 | 1.16 | 1.20 | 1.32 | 1.56 |
| Bn_04 | 1.02 | 1.42 | 1.09 | 0.51 | 0.89 | 0.32 | 1.10 | 0.91 | 0.77 | 0.97 | 0.97 |
| Bn_41 | 1.23 | 2.53 | 1.94 | 0.62 | 0.77 | 1.05 | 1.28 | 1.46 | 0.79 | 0.66 | 0.39 |
| Bn_43 | 1.33 | 1.30 | 1.23 | 1.14 | 1.52 | 0.32 | 0.83 | 0.77 | 0.67 | 0.90 | 1.03 |
| Bn_44 | 2.88 | 3.52 | 3.60 | 2.47 | 3.20 | 1.00 | 1.72 | 2.13 | 2.34 | 1.14 | 2.92 |
| Bn_46 | 1.07 | 0.95 | 1.07 | 0.78 | 1.05 | 0.71 | 0.96 | 0.71 | 0.75 | 0.67 | 1.04 |
| Bn_47 | 2.33 | 2.39 | 2.51 | 2.07 | 2.05 | 0.67 | 1.07 | 1.43 | 1.55 | 1.65 | 1.52 |
| Bn_49 | 4.85 | 5.09 | 4.72 | 1.20 | 5.76 | 3.11 | 4.72 | 3.62 | 3.32 | 2.13 | 4.84 |
| Bn_05 | 0.96 | 1.69 | 0.77 | 0.99 | 0.47 | 1.02 | 0.94 | 1.16 | 0.91 | 0.99 | 0.80 |
| Bn_50 | 2.39 | 2.29 | 1.25 | 0.36 | 0.71 | 0.40 | 0.77 | 0.59 | 1.47 | 1.76 | 1.24 |
| Bn_52 | 2.49 | 2.28 | 1.01 | 0.38 | 0.65 | 0.40 | 0.77 | 0.64 | 1.44 | 1.79 | 1.07 |
| Bn_53 | 2.02 | 1.94 | 1.33 | 0.27 | 0.59 | 0.41 | 0.74 | 0.76 | 1.82 | 1.67 | 1.04 |
| Bn_54 | 1.28 | 1.37 | 1.03 | 0.87 | 1.32 | 0.25 | 1.06 | 1.43 | 0.75 | 1.34 | 1.00 |
| Bn_56 | 1.41 | 1.39 | 1.22 | 0.36 | 0.96 | 0.35 | 0.79 | 0.56 | 1.60 | 1.37 | 0.80 |
| Bn_59 | 1.28 | 1.43 | 1.27 | 1.17 | 1.68 | 0.20 | 0.58 | 0.50 | 0.64 | 0.99 | 0.86 |
| Bn_06 | 2.18 | 2.57 | 0.71 | 0.45 | 0.55 | 0.42 | 1.36 | 0.69 | 1.11 | 1.17 | 0.96 |
| Bn_60 | 1.17 | 3.43 | 2.70 | 1.00 | 1.88 | 1.49 | 1.12 | 2.46 | 0.94 | 1.22 | 0.61 |
| Bn_62 | 0.90 | 5.58 | 5.27 | 2.07 | 4.25 | 1.15 | 1.87 | 2.45 | 2.01 | 0.86 | 3.78 |
| Bn_63 | 0.92 | 1.26 | 1.60 | 0.29 | 1.29 | 1.41 | 0.57 | 1.27 | 1.04 | 0.85 | 0.91 |
| Bn_64 | 0.93 | 1.34 | 1.20 | 0.95 | 1.14 | 0.32 | 1.03 | 0.93 | 0.93 | 0.88 | 0.95 |
| Bn_67 | 0.84 | 1.11 | 1.35 | 1.01 | 1.43 | 0.37 | 0.51 | 0.66 | 0.35 | 0.45 | 1.09 |
| Bn_68 | 1.26 | 3.47 | 2.84 | 0.29 | 1.10 | 1.12 | 1.31 | 1.49 | 0.68 | 1.01 | 0.78 |
| Bn_07 | 2.20 | 2.40 | 0.98 | 0.36 | 0.64 | 0.41 | 0.89 | 0.64 | 1.11 | 1.23 | 0.78 |
| Bn_08 | 1.13 | 0.98 | 0.67 | 0.15 | 0.51 | 0.16 | 0.31 | 0.75 | 0.82 | 0.77 | 0.92 |
| Bn_09 | 1.05 | 1.26 | 1.05 | 0.55 | 0.90 | 0.34 | 0.94 | 0.97 | 0.73 | 0.85 | 1.12 |
| Fg | 1.19 | 1.19 | 1.07 | 0.27 | 1.81 | 0.44 | 0.25 | 0.84 | 0.82 | 1.22 | 1.10 |
| Rs | 0.95 | 0.93 | 0.97 | 0.28 | 0.66 | 0.47 | 0.67 | 0.51 | 0.71 | 0.61 | 0.84 |
| Ta_01 | 1.11 | 1.33 | 1.11 | 0.38 | 0.54 | 0.60 | 1.10 | 1.01 | 0.84 | 1.11 | 0.97 |
| Ta_10 | 0.92 | 3.11 | 2.97 | 0.84 | 2.11 | 0.59 | 1.12 | 1.13 | 1.14 | 1.20 | 2.60 |
| Ta_11 | 0.97 | 0.78 | 0.61 | 0.30 | 0.44 | 0.26 | 0.64 | 0.68 | 1.23 | 1.52 | 1.01 |
| Ta_12 | 1.71 | 2.24 | 1.41 | 0.91 | 1.64 | 0.32 | 0.36 | 0.56 | 0.95 | 0.77 | 0.51 |
| Ta_13 | 1.36 | 6.12 | 5.56 | 2.55 | 4.73 | 1.28 | 1.60 | 2.84 | 1.96 | 1.21 | 4.69 |
| Ta_14 | 0.71 | 0.93 | 1.04 | 0.55 | 0.94 | 0.68 | 0.71 | 0.55 | 0.52 | 0.76 | 0.59 |
| Ta_15 | 1.16 | 2.00 | 1.42 | 1.26 | 2.32 | 0.73 | 0.78 | 1.26 | 0.73 | 0.83 | 1.19 |
| Ta_16 | 1.24 | 1.63 | 1.37 | 0.70 | 1.24 | 0.75 | 0.95 | 1.08 | 1.21 | 1.14 | 1.35 |
| Ta_18 | 1.10 | 2.81 | 3.18 | 2.72 | 3.67 | 2.56 | 2.99 | 3.59 | 2.20 | 1.49 | 2.70 |
| Ta_19 | 1.86 | 2.81 | 2.49 | 0.96 | 0.89 | 0.56 | 0.82 | 1.79 | 0.97 | 0.75 | 0.82 |
| Ta_02 | 1.19 | 3.59 | 1.33 | 0.40 | 2.27 | 0.71 | 2.02 | 3.31 | 1.01 | 3.43 | 2.47 |
| Ta_20 | 1.42 | 1.76 | 1.54 | 0.57 | 1.18 | 0.40 | 0.87 | 0.97 | 0.90 | 1.02 | 1.55 |
| Ta_21 | 1.31 | 1.46 | 0.41 | 0.41 | 1.32 | 0.19 | 0.31 | 0.32 | 0.72 | 0.94 | 0.33 |
| Ta_22 | 1.05 | 1.29 | 1.07 | 0.63 | 0.79 | 0.41 | 0.63 | 0.69 | 1.07 | 0.83 | 1.06 |
| Ta_23 | 1.04 | 1.67 | 1.23 | 1.04 | 1.63 | 0.47 | 0.71 | 0.55 | 0.58 | 0.84 | 1.23 |
| Ta_24 | 2.78 | 6.46 | 5.15 | 1.17 | 2.05 | 1.78 | 2.17 | 2.31 | 1.54 | 1.04 | 2.13 |
| Ta_25 | 0.69 | 1.10 | 0.89 | 0.97 | 1.07 | 0.34 | 0.75 | 0.45 | 0.48 | 0.64 | 0.92 |
| Ta_26 | 1.01 | 1.31 | 1.09 | 0.45 | 0.81 | 0.44 | 0.76 | 0.79 | 0.86 | 0.95 | 1.06 |
| Ta_27 | 2.09 | 2.29 | 1.62 | 0.64 | 1.49 | 0.65 | 1.15 | 0.84 | 1.54 | 1.49 | 1.32 |
| Ta_28 | 2.01 | 1.99 | 0.83 | 0.44 | 0.90 | 0.52 | 1.04 | 0.66 | 1.44 | 1.10 | 0.59 |
| Ta_29 | 1.30 | 1.53 | 1.16 | 0.31 | 0.61 | 0.79 | 0.83 | 0.55 | 0.67 | 1.14 | 1.19 |
| Ta_03 | 0.49 | 0.72 | 0.66 | 0.31 | 1.09 | 0.19 | 0.27 | 0.54 | 0.50 | 0.59 | 0.64 |
| Ta_30 | 0.89 | 1.48 | 1.00 | 0.15 | 0.47 | 0.21 | 1.10 | 0.74 | 0.92 | 0.81 | 1.06 |
| Ta_31 | 1.03 | 2.42 | 1.73 | 1.11 | 1.08 | 1.08 | 0.95 | 1.55 | 1.34 | 0.92 | 0.99 |
| Ta_32 | 1.36 | 1.90 | 1.44 | 0.58 | 1.32 | 0.82 | 0.95 | 1.23 | 1.20 | 1.10 | 1.37 |
| Ta_33 | 1.02 | 1.45 | 1.20 | 0.40 | 0.89 | 0.42 | 0.66 | 0.96 | 0.92 | 0.70 | 1.23 |
| Ta_34 | 0.90 | 1.39 | 1.14 | 0.37 | 0.80 | 0.43 | 0.51 | 0.84 | 0.88 | 0.92 | 0.96 |
| Ta_35 | 1.08 | 1.56 | 1.21 | 0.56 | 1.06 | 0.68 | 0.64 | 0.93 | 0.90 | 0.89 | 1.20 |
| Ta_36 | 0.98 | 1.22 | 0.98 | 0.37 | 0.67 | 0.36 | 0.53 | 0.78 | 0.74 | 0.95 | 1.05 |
| Ta_37 | 2.25 | 4.77 | 2.44 | 2.00 | 4.33 | 1.10 | 1.16 | 1.32 | 1.31 | 1.13 | 1.94 |
| Ta_38 | 0.78 | 1.57 | 1.50 | 0.47 | 1.33 | 0.50 | 1.40 | 0.55 | 0.83 | 0.54 | 0.81 |
| Ta_39 | 0.88 | 2.72 | 2.62 | 2.58 | 3.13 | 2.30 | 2.37 | 3.11 | 2.32 | 1.56 | 2.70 |
| Ta_04 | 1.10 | 1.52 | 1.26 | 0.54 | 1.11 | 0.64 | 0.82 | 0.89 | 0.92 | 1.10 | 1.21 |
| Ta_40 | 0.54 | 1.44 | 1.28 | 0.71 | 1.23 | 1.06 | 1.02 | 0.89 | 0.73 | 0.69 | 1.19 |
| Ta_41 | 1.87 | 2.52 | 2.55 | 2.13 | 2.60 | 1.29 | 1.80 | 0.61 | 1.27 | 1.14 | 1.61 |
| Ta_05 | 1.40 | 1.69 | 1.20 | 0.21 | 0.74 | 0.29 | 1.12 | 1.07 | 1.01 | 0.87 | 1.28 |
| Ta_06 | 1.06 | 1.59 | 0.99 | 1.01 | 1.40 | 0.68 | 0.80 | 1.52 | 1.10 | 0.69 | 1.14 |
| Ta_07 | 1.25 | 1.83 | 1.30 | 0.45 | 0.56 | 0.36 | 0.77 | 0.76 | 0.68 | 0.70 | 1.28 |
| Ta_08 | 0.89 | 1.25 | 0.50 | 0.44 | 1.10 | 0.21 | 0.46 | 0.28 | 0.52 | 0.78 | 0.20 |
| Ta_09 | 1.50 | 6.60 | 5.53 | 2.14 | 4.50 | 1.14 | 1.97 | 2.81 | 1.84 | 1.14 | 4.68 |

| Isolates | D-Malic-Acid | L-Malic-Acid | Quinic-Acid | D-Saccharic-Acid | Sebacic-Acid | Succinamic-Acid | Succinic-Acid | Succinic-Acid_Mono-Methyl-Ester | N-Acethyl-L-Glutamic-Acid | Alaninamide | L-Alanine |
| --- | --- | --- | --- | --- | --- | --- | --- | --- | --- | --- | --- |
| Bn_01 | 0.85 | 0.39 | 1.15 | 0.44 | 0.31 | 0.16 | 0.90 | 0.23 | 0.30 | 1.05 | 0.80 |
| Bn_10 | 0.57 | 0.63 | 1.61 | 0.80 | 0.47 | 0.29 | 0.51 | 0.27 | 0.39 | 0.99 | 1.00 |
| Bn_11 | 1.12 | 1.12 | 1.84 | 0.85 | 0.77 | 0.70 | 0.84 | 1.03 | 0.30 | 1.22 | 1.72 |
| Bn_13 | 0.61 | 1.49 | 1.16 | 0.53 | 0.51 | 0.27 | 1.44 | 0.24 | 0.48 | 0.75 | 0.65 |
| Bn_14 | 0.76 | 0.82 | 1.35 | 0.77 | 0.34 | 0.85 | 1.11 | 0.30 | 0.63 | 0.80 | 1.21 |
| Bn_15 | 0.93 | 2.13 | 2.24 | 1.89 | 0.66 | 0.96 | 1.54 | 1.39 | 0.58 | 1.40 | 1.80 |
| Bn_16 | 0.80 | 0.66 | 1.11 | 0.93 | 0.36 | 0.78 | 1.09 | 0.57 | 0.57 | 0.51 | 0.83 |
| Bn_17 | 1.55 | 3.11 | 4.11 | 2.18 | 2.83 | 0.94 | 2.73 | 2.77 | 0.86 | 2.18 | 4.58 |
| Bn_18 | 3.54 | 4.44 | 4.79 | 4.61 | 0.78 | 3.62 | 4.15 | 2.43 | 1.02 | 1.18 | 4.67 |
| Bn_19 | 0.66 | 0.84 | 0.82 | 0.72 | 2.03 | 0.63 | 1.19 | 1.82 | 0.61 | 1.67 | 3.50 |
| Bn_21 | 0.49 | 1.00 | 0.70 | 0.66 | 0.61 | 0.36 | 0.73 | 0.21 | 0.32 | 3.35 | 2.43 |
| Bn_22 | 1.55 | 1.06 | 2.55 | 2.41 | 1.96 | 1.36 | 1.37 | 1.08 | 0.46 | 1.53 | 1.91 |
| Bn_23 | 0.51 | 0.86 | 1.47 | 1.00 | 0.32 | 0.36 | 0.94 | 0.14 | 0.66 | 0.63 | 1.27 |
| Bn_24 | 0.79 | 1.27 | 1.33 | 1.04 | 0.60 | 0.79 | 1.00 | 0.79 | 0.69 | 1.27 | 1.41 |
| Bn_25 | 0.41 | 0.72 | 3.55 | 0.64 | 1.33 | 0.73 | 0.59 | 0.85 | 0.55 | 0.51 | 1.91 |
| Bn_26 | 2.22 | 1.06 | 4.24 | 3.42 | 1.18 | 1.44 | 1.70 | 2.67 | 1.36 | 1.36 | 3.82 |
| Bn_27 | 0.85 | 0.90 | 1.35 | 0.64 | 0.63 | 0.53 | 0.73 | 0.22 | 0.51 | 2.26 | 1.50 |
| Bn_28 | 0.66 | 0.81 | 1.36 | 1.06 | 0.55 | 0.31 | 0.87 | 0.54 | 0.38 | 1.03 | 1.19 |
| Bn_29 | 1.03 | 1.23 | 1.97 | 0.99 | 0.81 | 0.95 | 1.10 | 1.09 | 0.33 | 1.04 | 1.65 |
| Bn_03 | 0.48 | 0.52 | 0.72 | 0.66 | 0.31 | 0.19 | 0.61 | 0.21 | 0.32 | 0.99 | 0.95 |
| Bn_30 | 0.83 | 0.90 | 1.00 | 0.84 | 0.30 | 0.25 | 0.97 | 0.27 | 0.67 | 0.89 | 0.95 |
| Bn_31 | 1.01 | 0.52 | 1.60 | 0.83 | 0.96 | 0.54 | 0.97 | 0.67 | 0.47 | 0.52 | 1.78 |
| Bn_32 | 1.10 | 1.51 | 4.57 | 1.37 | 4.11 | 1.71 | 1.24 | 3.76 | 0.90 | 0.71 | 4.43 |
| Bn_33 | 3.33 | 3.64 | 5.70 | 3.08 | 3.79 | 1.39 | 4.35 | 2.96 | 1.11 | 2.78 | 5.97 |
| Bn_34 | 1.74 | 1.74 | 2.95 | 2.80 | 2.08 | 1.36 | 1.51 | 1.53 | 0.53 | 0.85 | 2.56 |
| Bn_36 | 2.81 | 3.38 | 0.62 | 3.41 | 0.85 | 0.72 | 3.38 | 0.26 | 1.99 | 0.87 | 3.14 |
| Bn_39 | 1.19 | 1.28 | 1.78 | 1.75 | 0.54 | 1.22 | 1.32 | 0.23 | 0.77 | 0.85 | 1.45 |
| Bn_04 | 0.84 | 0.86 | 1.20 | 0.80 | 0.42 | 0.56 | 1.05 | 0.18 | 0.98 | 0.93 | 1.26 |
| Bn_41 | 0.65 | 0.79 | 2.50 | 1.74 | 1.57 | 0.94 | 1.09 | 1.06 | 0.55 | 0.90 | 2.05 |
| Bn_43 | 0.57 | 1.28 | 1.67 | 1.31 | 0.44 | 0.47 | 1.49 | 0.73 | 0.81 | 0.93 | 1.17 |
| Bn_44 | 2.14 | 2.82 | 3.43 | 1.79 | 1.81 | 2.31 | 2.26 | 1.86 | 0.83 | 2.07 | 3.30 |
| Bn_46 | 0.99 | 0.99 | 0.92 | 0.90 | 0.33 | 0.38 | 0.99 | 0.19 | 0.54 | 0.81 | 0.83 |
| Bn_47 | 2.00 | 2.20 | 2.32 | 1.71 | 1.28 | 1.13 | 1.93 | 1.20 | 0.40 | 1.59 | 1.94 |
| Bn_49 | 1.21 | 5.83 | 5.75 | 4.76 | 2.82 | 1.41 | 5.45 | 3.24 | 0.96 | 1.80 | 5.93 |
| Bn_05 | 1.00 | 0.70 | 1.13 | 0.68 | 0.60 | 0.39 | 0.77 | 0.27 | 0.48 | 1.22 | 1.50 |
| Bn_50 | 0.56 | 1.00 | 0.77 | 0.64 | 2.03 | 0.51 | 1.33 | 1.79 | 0.45 | 1.47 | 3.09 |
| Bn_52 | 0.57 | 0.73 | 0.81 | 0.62 | 2.09 | 0.55 | 0.95 | 1.76 | 0.62 | 1.58 | 3.16 |
| Bn_53 | 0.60 | 0.63 | 1.18 | 0.50 | 1.73 | 0.44 | 1.02 | 1.42 | 0.46 | 1.55 | 2.63 |
| Bn_54 | 0.36 | 1.46 | 1.56 | 0.87 | 0.47 | 1.25 | 1.32 | 0.42 | 0.90 | 1.13 | 1.36 |
| Bn_56 | 0.52 | 0.78 | 0.66 | 0.48 | 1.64 | 0.40 | 0.96 | 1.33 | 0.54 | 1.42 | 2.12 |
| Bn_59 | 0.57 | 1.29 | 1.77 | 1.50 | 0.44 | 0.49 | 1.29 | 0.69 | 0.36 | 1.01 | 1.18 |
| Bn_06 | 0.63 | 0.78 | 0.96 | 0.88 | 1.94 | 0.58 | 0.64 | 1.17 | 0.48 | 1.49 | 3.84 |
| Bn_60 | 1.97 | 1.93 | 3.64 | 2.65 | 2.57 | 1.55 | 1.74 | 1.61 | 0.65 | 1.03 | 2.75 |
| Bn_62 | 3.00 | 3.07 | 5.70 | 2.97 | 3.47 | 1.21 | 3.40 | 2.78 | 0.89 | 2.02 | 5.27 |
| Bn_63 | 0.55 | 1.01 | 2.00 | 0.75 | 0.43 | 0.52 | 1.19 | 0.76 | 0.51 | 1.13 | 1.09 |
| Bn_64 | 0.79 | 0.82 | 1.27 | 0.94 | 0.23 | 0.56 | 0.95 | 0.15 | 0.48 | 0.95 | 1.01 |
| Bn_67 | 0.60 | 1.41 | 1.44 | 1.48 | 0.43 | 1.05 | 1.01 | 0.20 | 0.37 | 0.60 | 0.72 |
| Bn_68 | 1.61 | 0.73 | 3.27 | 2.39 | 1.03 | 1.09 | 0.91 | 1.89 | 1.36 | 1.79 | 3.08 |
| Bn_07 | 0.51 | 0.65 | 0.77 | 0.60 | 2.40 | 0.51 | 0.82 | 1.59 | 0.55 | 1.14 | 3.15 |
| Bn_08 | 0.48 | 0.71 | 0.79 | 0.80 | 0.85 | 0.24 | 0.47 | 0.51 | 0.43 | 1.35 | 1.42 |
| Bn_09 | 0.75 | 0.91 | 1.09 | 0.81 | 0.48 | 0.45 | 1.13 | 0.25 | 1.03 | 1.00 | 0.86 |
| Fg | 1.35 | 1.27 | 2.13 | 0.57 | 0.73 | 1.06 | 1.20 | 0.26 | 1.17 | 0.97 | 0.62 |
| Rs | 0.63 | 0.71 | 1.00 | 0.64 | 0.25 | 0.34 | 1.00 | 0.09 | 0.81 | 0.72 | 0.86 |
| Ta_01 | 0.51 | 0.49 | 1.24 | 0.64 | 0.41 | 0.30 | 0.93 | 0.24 | 0.55 | 1.08 | 1.18 |
| Ta_10 | 1.74 | 2.46 | 3.92 | 1.72 | 2.02 | 1.06 | 2.35 | 1.39 | 0.96 | 2.48 | 4.07 |
| Ta_11 | 0.98 | 0.96 | 1.01 | 1.12 | 0.66 | 0.55 | 0.86 | 0.21 | 0.83 | 2.94 | 3.16 |
| Ta_12 | 0.69 | 1.13 | 1.56 | 1.78 | 0.60 | 1.51 | 1.62 | 0.19 | 0.23 | 1.50 | 1.58 |
| Ta_13 | 3.39 | 3.76 | 5.59 | 3.50 | 4.19 | 1.70 | 3.98 | 3.07 | 1.44 | 3.50 | 5.75 |
| Ta_14 | 0.50 | 0.53 | 0.96 | 0.44 | 0.36 | 0.33 | 0.69 | 0.11 | 0.46 | 0.45 | 0.48 |
| Ta_15 | 0.95 | 1.09 | 2.43 | 1.12 | 0.94 | 1.21 | 1.96 | 1.34 | 0.49 | 0.47 | 1.13 |
| Ta_16 | 1.11 | 1.09 | 1.59 | 1.17 | 0.64 | 0.85 | 1.14 | 0.94 | 0.72 | 1.28 | 1.51 |
| Ta_18 | 3.41 | 3.48 | 3.61 | 3.73 | 0.94 | 2.99 | 3.59 | 2.10 | 1.10 | 0.96 | 3.81 |
| Ta_19 | 0.74 | 1.00 | 2.64 | 2.21 | 1.06 | 0.84 | 0.84 | 1.37 | 0.80 | 1.63 | 2.36 |
| Ta_02 | 1.43 | 1.47 | 2.89 | 0.51 | 0.83 | 1.15 | 2.46 | 0.36 | 1.69 | 2.24 | 0.89 |
| Ta_20 | 1.07 | 0.83 | 1.77 | 1.47 | 0.45 | 0.57 | 1.09 | 0.85 | 0.43 | 1.30 | 1.54 |
| Ta_21 | 0.43 | 1.23 | 1.25 | 1.54 | 0.64 | 1.21 | 0.93 | 0.83 | 0.23 | 0.93 | 1.29 |
| Ta_22 | 0.75 | 0.80 | 1.26 | 0.97 | 0.56 | 0.59 | 0.76 | 0.65 | 0.39 | 0.84 | 1.13 |
| Ta_23 | 0.75 | 1.55 | 1.71 | 1.50 | 0.56 | 0.68 | 1.61 | 0.77 | 0.60 | 1.06 | 1.35 |
| Ta_24 | 2.72 | 1.79 | 7.03 | 2.62 | 1.86 | 1.99 | 2.60 | 3.94 | 1.69 | 2.12 | 6.40 |
| Ta_25 | 0.70 | 0.99 | 1.22 | 0.65 | 0.29 | 0.67 | 1.31 | 0.47 | 0.64 | 0.89 | 0.69 |
| Ta_26 | 0.77 | 0.75 | 1.25 | 0.91 | 0.41 | 0.45 | 0.77 | 0.77 | 0.51 | 0.95 | 1.11 |
| Ta_27 | 0.90 | 1.23 | 1.28 | 0.78 | 2.03 | 0.67 | 1.63 | 1.77 | 0.53 | 1.45 | 2.67 |
| Ta_28 | 0.74 | 0.72 | 1.04 | 0.72 | 1.22 | 0.53 | 0.99 | 0.84 | 0.55 | 1.56 | 2.33 |
| Ta_29 | 0.52 | 0.64 | 1.33 | 0.76 | 0.35 | 0.54 | 0.54 | 0.59 | 0.25 | 1.33 | 1.37 |
| Ta_03 | 0.42 | 0.65 | 0.91 | 0.99 | 0.22 | 0.88 | 1.03 | 0.15 | 0.26 | 0.72 | 0.87 |
| Ta_30 | 0.59 | 0.70 | 1.34 | 0.74 | 0.50 | 0.25 | 0.84 | 0.49 | 0.50 | 0.98 | 0.93 |
| Ta_31 | 1.32 | 1.27 | 2.05 | 1.27 | 0.60 | 1.44 | 1.24 | 0.80 | 0.79 | 1.12 | 1.49 |
| Ta_32 | 1.13 | 1.15 | 1.71 | 1.12 | 0.67 | 0.82 | 1.24 | 1.08 | 0.72 | 1.30 | 1.55 |
| Ta_33 | 1.00 | 0.94 | 1.38 | 0.89 | 0.53 | 0.57 | 1.01 | 0.78 | 0.67 | 0.98 | 1.11 |
| Ta_34 | 0.78 | 0.76 | 1.24 | 0.92 | 0.49 | 0.57 | 0.94 | 0.67 | 0.65 | 0.67 | 1.01 |
| Ta_35 | 0.92 | 0.97 | 1.49 | 0.92 | 0.48 | 0.65 | 1.01 | 0.86 | 0.54 | 1.06 | 1.27 |
| Ta_36 | 0.51 | 0.54 | 1.26 | 1.00 | 0.62 | 0.36 | 0.63 | 0.65 | 0.44 | 0.91 | 1.15 |
| Ta_37 | 2.28 | 2.42 | 3.95 | 2.02 | 1.41 | 2.20 | 3.30 | 1.27 | 1.65 | 2.25 | 2.41 |
| Ta_38 | 0.82 | 0.72 | 0.97 | 0.76 | 0.33 | 0.89 | 1.25 | 0.22 | 0.63 | 0.40 | 0.77 |
| Ta_39 | 2.90 | 3.09 | 3.16 | 3.23 | 0.91 | 2.58 | 2.95 | 1.50 | 0.64 | 1.21 | 3.07 |
| Ta_04 | 1.06 | 1.10 | 1.41 | 1.01 | 0.49 | 0.72 | 1.11 | 0.93 | 0.61 | 1.10 | 0.96 |
| Ta_40 | 0.96 | 1.17 | 1.44 | 0.90 | 0.42 | 1.10 | 1.19 | 0.67 | 0.42 | 0.88 | 1.14 |
| Ta_41 | 0.56 | 2.46 | 2.80 | 2.02 | 0.98 | 1.71 | 2.66 | 1.61 | 1.73 | 1.36 | 2.33 |
| Ta_05 | 0.85 | 0.87 | 1.52 | 1.01 | 0.54 | 0.46 | 1.01 | 0.72 | 0.42 | 0.85 | 1.21 |
| Ta_06 | 0.72 | 0.85 | 1.75 | 0.87 | 0.48 | 1.08 | 0.97 | 0.18 | 0.71 | 0.40 | 1.16 |
| Ta_07 | 0.55 | 0.68 | 1.83 | 1.32 | 0.60 | 0.43 | 0.61 | 0.64 | 0.32 | 0.87 | 1.26 |
| Ta_08 | 0.27 | 0.64 | 0.77 | 0.59 | 0.47 | 0.61 | 0.89 | 0.58 | 0.18 | 0.46 | 1.04 |
| Ta_09 | 3.24 | 3.39 | 5.70 | 3.36 | 4.35 | 1.39 | 4.12 | 3.18 | 1.35 | 3.82 | 6.04 |

| Isolates | L-Alanyl-Glycine | L-Asparagine | L-Aspartic-Acid | L-Glutamic-Acid | Glycyl-L-Gutamic-Acid | L-Ornithine | L-Phenylalanine | L-Proline | L-Pyroglutamic-Acid | L-Serine |
| --- | --- | --- | --- | --- | --- | --- | --- | --- | --- | --- |
| Bn_01 | 0.76 | 0.87 | 1.05 | 1.09 | 0.72 | 1.21 | 1.08 | 1.05 | 1.09 | 0.97 |
| Bn_10 | 1.04 | 1.48 | 0.77 | 0.78 | 0.79 | 0.74 | 0.93 | 0.78 | 0.74 | 1.22 |
| Bn_11 | 1.01 | 1.60 | 1.30 | 1.66 | 0.60 | 1.46 | 1.40 | 1.78 | 1.30 | 1.54 |
| Bn_13 | 0.78 | 0.41 | 1.49 | 1.58 | 0.55 | 0.42 | 0.74 | 0.36 | 0.33 | 0.39 |
| Bn_14 | 1.05 | 1.34 | 0.91 | 1.38 | 1.18 | 0.79 | 0.72 | 1.26 | 1.13 | 1.28 |
| Bn_15 | 1.08 | 2.42 | 2.42 | 2.84 | 1.64 | 1.45 | 1.38 | 2.31 | 1.37 | 1.61 |
| Bn_16 | 1.03 | 0.97 | 1.19 | 1.22 | 0.78 | 0.67 | 0.30 | 1.24 | 1.01 | 0.94 |
| Bn_17 | 3.19 | 3.35 | 4.68 | 5.26 | 1.19 | 3.58 | 2.10 | 5.03 | 4.20 | 4.54 |
| Bn_18 | 4.40 | 4.47 | 4.70 | 4.81 | 2.10 | 4.74 | 4.77 | 4.80 | 3.75 | 4.10 |
| Bn_19 | 3.34 | 3.50 | 3.20 | 3.75 | 1.65 | 1.62 | 1.65 | 1.31 | 0.59 | 1.22 |
| Bn_21 | 2.10 | 1.79 | 2.50 | 3.17 | 2.23 | 0.76 | 0.43 | 0.81 | 0.35 | 0.82 |
| Bn_22 | 2.41 | 2.16 | 1.23 | 2.48 | 0.84 | 1.67 | 1.40 | 2.17 | 2.27 | 2.40 |
| Bn_23 | 1.20 | 1.33 | 1.30 | 1.36 | 1.03 | 0.85 | 0.84 | 0.90 | 0.60 | 1.38 |
| Bn_24 | 1.08 | 1.21 | 1.26 | 1.27 | 0.91 | 1.08 | 0.73 | 1.43 | 1.40 | 1.35 |
| Bn_25 | 1.24 | 2.33 | 2.87 | 3.22 | 0.76 | 0.92 | 0.47 | 2.45 | 0.94 | 2.49 |
| Bn_26 | 2.71 | 3.20 | 3.62 | 3.97 | 1.54 | 2.74 | 1.54 | 4.07 | 3.09 | 3.26 |
| Bn_27 | 2.47 | 1.50 | 2.23 | 2.61 | 2.14 | 1.49 | 0.47 | 1.86 | 0.48 | 1.70 |
| Bn_28 | 0.86 | 1.15 | 1.19 | 1.38 | 1.04 | 1.00 | 0.58 | 1.30 | 0.52 | 1.21 |
| Bn_29 | 1.47 | 1.55 | 1.47 | 1.71 | 0.81 | 1.53 | 1.45 | 1.86 | 1.49 | 1.60 |
| Bn_03 | 0.76 | 0.59 | 0.94 | 0.60 | 1.03 | 0.82 | 0.75 | 0.55 | 0.67 | 0.62 |
| Bn_30 | 0.82 | 0.79 | 0.95 | 1.01 | 1.04 | 0.95 | 0.81 | 1.03 | 0.99 | 0.85 |
| Bn_31 | 1.33 | 0.97 | 0.84 | 1.87 | 1.20 | 1.27 | 1.39 | 1.38 | 1.07 | 1.15 |
| Bn_32 | 2.74 | 3.73 | 2.10 | 3.81 | 2.55 | 1.49 | 2.03 | 3.59 | 2.47 | 3.08 |
| Bn_33 | 4.81 | 5.39 | 5.69 | 6.69 | 2.28 | 5.58 | 3.96 | 6.74 | 5.45 | 6.22 |
| Bn_34 | 2.72 | 2.45 | 1.90 | 2.83 | 0.71 | 1.95 | 0.97 | 2.44 | 2.54 | 2.60 |
| Bn_36 | 2.57 | 3.34 | 2.40 | 2.33 | 2.96 | 1.03 | 0.85 | 3.57 | 0.58 | 3.05 |
| Bn_39 | 1.47 | 1.99 | 1.61 | 1.75 | 1.36 | 1.71 | 1.73 | 1.96 | 1.78 | 1.62 |
| Bn_04 | 0.98 | 0.93 | 1.13 | 1.10 | 0.99 | 1.00 | 0.96 | 1.09 | 1.03 | 0.93 |
| Bn_41 | 1.67 | 2.51 | 1.72 | 2.29 | 0.71 | 1.92 | 1.13 | 1.95 | 2.07 | 2.14 |
| Bn_43 | 1.00 | 1.49 | 1.64 | 1.76 | 1.51 | 1.05 | 0.83 | 1.51 | 1.33 | 1.07 |
| Bn_44 | 2.84 | 2.31 | 2.74 | 3.36 | 1.04 | 2.27 | 2.62 | 1.86 | 1.15 | 2.76 |
| Bn_46 | 0.80 | 0.83 | 0.72 | 0.83 | 0.55 | 0.81 | 0.74 | 0.92 | 1.03 | 0.63 |
| Bn_47 | 1.59 | 1.40 | 1.51 | 2.32 | 0.78 | 1.70 | 1.58 | 2.17 | 0.74 | 1.89 |
| Bn_49 | 4.73 | 6.37 | 5.96 | 5.96 | 4.45 | 2.29 | 3.46 | 6.11 | 5.75 | 5.29 |
| Bn_05 | 1.44 | 1.40 | 1.19 | 1.17 | 1.12 | 1.26 | 0.80 | 0.81 | 1.55 | 1.22 |
| Bn_50 | 2.89 | 3.18 | 2.80 | 3.23 | 1.43 | 1.58 | 1.92 | 2.39 | 0.41 | 0.97 |
| Bn_52 | 2.94 | 3.23 | 2.89 | 3.42 | 1.59 | 1.64 | 1.99 | 2.19 | 0.45 | 1.06 |
| Bn_53 | 2.33 | 2.53 | 2.58 | 2.75 | 1.69 | 1.27 | 1.69 | 2.08 | 0.41 | 0.77 |
| Bn_54 | 1.18 | 0.95 | 1.51 | 1.78 | 1.32 | 0.93 | 0.85 | 1.27 | 1.18 | 1.08 |
| Bn_56 | 2.04 | 2.10 | 1.97 | 2.17 | 1.29 | 1.28 | 1.52 | 1.74 | 0.37 | 1.17 |
| Bn_59 | 0.75 | 1.42 | 1.62 | 1.84 | 1.47 | 1.21 | 0.65 | 1.68 | 0.54 | 1.00 |
| Bn_06 | 2.98 | 3.82 | 3.60 | 4.24 | 1.58 | 2.23 | 2.33 | 1.70 | 0.76 | 1.20 |
| Bn_60 | 2.60 | 3.30 | 2.20 | 3.35 | 1.08 | 2.43 | 1.89 | 3.28 | 3.15 | 3.26 |
| Bn_62 | 4.15 | 4.74 | 5.15 | 6.49 | 1.36 | 4.67 | 3.01 | 6.07 | 4.55 | 5.34 |
| Bn_63 | 1.10 | 1.47 | 1.20 | 1.87 | 0.88 | 0.73 | 0.87 | 1.07 | 0.38 | 0.75 |
| Bn_64 | 1.00 | 1.05 | 1.08 | 1.21 | 0.93 | 1.07 | 0.95 | 1.19 | 1.00 | 0.92 |
| Bn_67 | 1.25 | 1.17 | 1.42 | 1.97 | 0.75 | 1.57 | 0.69 | 0.70 | 0.38 | 1.10 |
| Bn_68 | 1.65 | 1.90 | 2.47 | 2.92 | 1.46 | 1.80 | 0.88 | 3.58 | 2.57 | 2.21 |
| Bn_07 | 2.65 | 3.00 | 3.16 | 3.44 | 1.42 | 1.11 | 1.68 | 1.64 | 0.45 | 0.95 |
| Bn_08 | 1.40 | 1.41 | 1.52 | 1.55 | 1.41 | 1.40 | 0.83 | 1.15 | 0.47 | 1.12 |
| Bn_09 | 0.97 | 0.89 | 1.14 | 1.12 | 1.08 | 1.16 | 1.02 | 1.08 | 1.06 | 0.90 |
| Fg | 0.88 | 1.25 | 1.59 | 1.42 | 0.36 | 1.05 | 1.19 | 1.09 | 0.86 | 1.40 |
| Rs | 0.64 | 0.55 | 1.10 | 1.39 | 0.97 | 0.81 | 0.46 | 0.97 | 0.70 | 0.80 |
| Ta_01 | 0.98 | 0.90 | 1.31 | 1.29 | 1.15 | 1.10 | 0.80 | 1.18 | 1.24 | 0.94 |
| Ta_10 | 3.53 | 4.01 | 3.63 | 4.61 | 2.95 | 2.88 | 1.52 | 4.36 | 2.24 | 3.38 |
| Ta_11 | 3.18 | 3.08 | 3.09 | 3.32 | 3.22 | 3.19 | 1.68 | 2.10 | 0.57 | 2.28 |
| Ta_12 | 1.79 | 1.52 | 1.68 | 2.30 | 1.51 | 1.64 | 1.90 | 1.13 | 0.39 | 1.01 |
| Ta_13 | 4.75 | 5.05 | 5.40 | 6.44 | 1.80 | 4.83 | 3.67 | 6.48 | 4.68 | 5.78 |
| Ta_14 | 0.43 | 0.56 | 0.58 | 0.91 | 1.06 | 0.33 | 0.69 | 1.16 | 0.95 | 0.64 |
| Ta_15 | 1.29 | 1.37 | 2.20 | 2.29 | 1.25 | 1.00 | 0.84 | 1.94 | 1.75 | 0.94 |
| Ta_16 | 1.43 | 1.40 | 1.28 | 1.51 | 0.90 | 1.26 | 0.96 | 1.63 | 1.24 | 1.48 |
| Ta_18 | 3.58 | 3.73 | 3.84 | 2.68 | 1.85 | 3.78 | 3.91 | 3.92 | 3.56 | 3.47 |
| Ta_19 | 2.31 | 2.76 | 2.40 | 2.63 | 1.23 | 1.94 | 1.42 | 2.69 | 2.37 | 2.59 |
| Ta_02 | 1.57 | 2.44 | 3.01 | 2.51 | 1.56 | 0.33 | 1.83 | 1.16 | 2.85 | 0.63 |
| Ta_20 | 1.17 | 1.51 | 1.60 | 1.69 | 0.84 | 1.41 | 0.93 | 1.81 | 1.43 | 1.59 |
| Ta_21 | 1.28 | 1.01 | 0.68 | 0.69 | 1.25 | 1.14 | 1.33 | 1.35 | 0.34 | 1.14 |
| Ta_22 | 0.81 | 1.09 | 1.08 | 1.22 | 0.72 | 0.86 | 0.76 | 1.20 | 0.86 | 1.11 |
| Ta_23 | 1.33 | 1.66 | 1.84 | 1.89 | 1.59 | 1.36 | 0.84 | 1.86 | 1.19 | 1.17 |
| Ta_24 | 3.90 | 5.32 | 5.73 | 6.87 | 2.76 | 2.82 | 2.15 | 5.81 | 3.37 | 4.59 |
| Ta_25 | 0.94 | 1.16 | 1.32 | 1.50 | 0.97 | 0.65 | 0.25 | 0.89 | 0.75 | 0.78 |
| Ta_26 | 0.88 | 0.85 | 1.15 | 1.25 | 0.77 | 0.92 | 0.72 | 1.24 | 0.93 | 1.07 |
| Ta_27 | 2.18 | 2.03 | 2.41 | 2.91 | 1.54 | 1.83 | 1.89 | 2.15 | 0.68 | 1.19 |
| Ta_28 | 2.36 | 2.39 | 2.32 | 2.43 | 2.29 | 1.30 | 1.30 | 2.07 | 0.67 | 1.56 |
| Ta_29 | 1.28 | 1.20 | 1.33 | 1.42 | 1.23 | 1.27 | 1.21 | 1.50 | 1.20 | 1.01 |
| Ta_03 | 0.86 | 0.82 | 1.03 | 1.27 | 1.00 | 0.76 | 0.59 | 1.10 | 1.16 | 1.21 |
| Ta_30 | 0.93 | 0.72 | 0.98 | 1.33 | 0.94 | 0.83 | 0.78 | 1.01 | 0.66 | 0.92 |
| Ta_31 | 1.32 | 1.96 | 1.80 | 2.14 | 1.03 | 1.55 | 1.22 | 1.89 | 1.93 | 1.50 |
| Ta_32 | 1.06 | 1.36 | 1.37 | 1.57 | 1.05 | 1.10 | 0.78 | 1.67 | 1.16 | 1.55 |
| Ta_33 | 0.87 | 1.22 | 1.25 | 1.34 | 1.19 | 0.67 | 0.65 | 1.39 | 0.56 | 0.99 |
| Ta_34 | 0.84 | 1.07 | 1.05 | 0.85 | 1.03 | 0.95 | 0.74 | 1.25 | 0.87 | 1.13 |
| Ta_35 | 0.91 | 1.11 | 1.18 | 1.32 | 0.94 | 0.96 | 0.64 | 1.45 | 0.97 | 1.23 |
| Ta_36 | 0.90 | 1.13 | 0.69 | 1.25 | 0.65 | 1.03 | 0.71 | 1.23 | 0.75 | 1.13 |
| Ta_37 | 3.74 | 2.85 | 3.75 | 3.56 | 2.75 | 1.83 | 2.06 | 1.94 | 1.82 | 2.56 |
| Ta_38 | 0.75 | 0.62 | 0.84 | 0.93 | 1.35 | 0.60 | 0.69 | 0.96 | 1.00 | 0.85 |
| Ta_39 | 2.90 | 3.18 | 3.26 | 3.33 | 1.88 | 3.17 | 3.17 | 3.30 | 2.63 | 2.78 |
| Ta_04 | 0.96 | 1.15 | 1.30 | 1.36 | 0.93 | 1.21 | 0.95 | 1.51 | 1.17 | 1.36 |
| Ta_40 | 1.15 | 1.48 | 1.32 | 1.56 | 0.98 | 1.43 | 1.00 | 1.54 | 1.39 | 1.37 |
| Ta_41 | 1.18 | 2.07 | 2.68 | 2.84 | 2.07 | 1.34 | 0.93 | 2.90 | 2.50 | 1.90 |
| Ta_05 | 1.19 | 1.19 | 1.32 | 1.44 | 1.27 | 1.06 | 0.81 | 1.47 | 0.60 | 1.27 |
| Ta_06 | 1.14 | 1.08 | 0.83 | 1.17 | 0.87 | 0.64 | 0.82 | 1.04 | 0.94 | 0.15 |
| Ta_07 | 0.91 | 1.43 | 1.34 | 1.73 | 0.98 | 0.83 | 0.54 | 1.68 | 0.80 | 1.31 |
| Ta_08 | 1.05 | 0.88 | 0.86 | 0.95 | 0.88 | 0.79 | 1.38 | 1.45 | 0.33 | 1.16 |
| Ta_09 | 4.85 | 5.30 | 5.24 | 6.45 | 1.90 | 5.32 | 2.93 | 6.54 | 5.05 | 5.91 |

| Isolates | L-Threonine | 2-Amino-Ethanol | Putrescine | Adenosine | Uridine | Adenosine-5'-Monophosphate |
| --- | --- | --- | --- | --- | --- | --- |
| Bn_01 | 0.76 | 0.58 | 0.91 | 0.55 | 1.07 | 0.86 |
| Bn_10 | 0.94 | 0.58 | 1.25 | 1.20 | 0.99 | 1.11 |
| Bn_11 | 1.38 | 0.50 | 1.38 | 0.72 | 0.95 | 0.76 |
| Bn_13 | 0.42 | 0.39 | 0.38 | 0.87 | 0.99 | 1.10 |
| Bn_14 | 0.89 | 0.35 | 0.99 | 1.02 | 0.63 | 0.81 |
| Bn_15 | 1.16 | 0.78 | 2.15 | 1.68 | 1.22 | 1.22 |
| Bn_16 | 0.58 | 0.61 | 1.29 | 0.99 | 1.00 | 0.49 |
| Bn_17 | 3.01 | 1.38 | 2.31 | 3.77 | 0.81 | 1.24 |
| Bn_18 | 4.31 | 3.25 | 3.78 | 1.05 | 1.21 | 1.08 |
| Bn_19 | 1.23 | 0.52 | 2.58 | 0.76 | 1.26 | 1.14 |
| Bn_21 | 1.26 | 0.73 | 0.73 | 2.64 | 0.65 | 2.20 |
| Bn_22 | 1.58 | 1.14 | 1.53 | 1.76 | 0.83 | 1.78 |
| Bn_23 | 0.50 | 0.32 | 1.07 | 0.88 | 1.35 | 0.93 |
| Bn_24 | 0.89 | 0.68 | 1.44 | 1.44 | 0.84 | 1.16 |
| Bn_25 | 1.56 | 0.81 | 0.95 | 1.14 | 0.75 | 1.18 |
| Bn_26 | 2.12 | 1.57 | 2.46 | 3.38 | 1.15 | 2.05 |
| Bn_27 | 0.88 | 0.61 | 0.73 | 2.03 | 1.73 | 0.97 |
| Bn_28 | 0.96 | 0.58 | 0.99 | 1.00 | 1.11 | 0.87 |
| Bn_29 | 1.44 | 0.83 | 1.36 | 0.75 | 1.10 | 1.17 |
| Bn_03 | 1.08 | 0.42 | 0.77 | 0.65 | 0.75 | 0.74 |
| Bn_30 | 0.73 | 0.71 | 0.88 | 0.99 | 0.95 | 0.91 |
| Bn_31 | 0.97 | 0.80 | 0.85 | 0.96 | 0.72 | 0.99 |
| Bn_32 | 2.85 | 2.11 | 1.39 | 3.90 | 2.11 | 2.82 |
| Bn_33 | 3.81 | 2.00 | 3.81 | 4.72 | 1.29 | 1.77 |
| Bn_34 | 1.58 | 1.02 | 1.45 | 2.18 | 1.25 | 2.05 |
| Bn_36 | 0.90 | 1.19 | 0.77 | 3.50 | 2.11 | 3.70 |
| Bn_39 | 1.88 | 1.03 | 1.87 | 1.02 | 1.40 | 1.23 |
| Bn_04 | 0.69 | 0.72 | 0.94 | 0.94 | 0.99 | 1.00 |
| Bn_41 | 1.94 | 0.93 | 1.43 | 2.04 | 1.02 | 1.52 |
| Bn_43 | 0.85 | 0.64 | 1.00 | 1.05 | 1.21 | 0.89 |
| Bn_44 | 2.46 | 2.77 | 2.64 | 1.57 | 1.28 | 1.04 |
| Bn_46 | 0.70 | 0.82 | 0.72 | 0.94 | 1.00 | 0.57 |
| Bn_47 | 2.35 | 1.39 | 1.64 | 1.13 | 1.32 | 0.99 |
| Bn_49 | 1.38 | 5.52 | 4.96 | 4.82 | 4.61 | 1.28 |
| Bn_05 | 0.89 | 0.60 | 1.06 | 1.04 | 1.03 | 1.19 |
| Bn_50 | 1.24 | 0.44 | 2.37 | 0.74 | 1.23 | 0.84 |
| Bn_52 | 1.49 | 0.49 | 2.38 | 1.30 | 1.32 | 1.08 |
| Bn_53 | 1.33 | 0.40 | 1.99 | 0.64 | 1.06 | 0.99 |
| Bn_54 | 0.85 | 0.96 | 1.43 | 0.96 | 0.98 | 0.86 |
| Bn_56 | 1.46 | 0.39 | 1.68 | 1.05 | 1.14 | 0.97 |
| Bn_59 | 0.57 | 0.75 | 0.92 | 0.55 | 0.82 | 0.86 |
| Bn_06 | 1.20 | 0.73 | 2.87 | 1.87 | 1.51 | 1.15 |
| Bn_60 | 2.31 | 1.46 | 2.10 | 2.16 | 1.59 | 2.45 |
| Bn_62 | 2.76 | 1.32 | 2.50 | 3.40 | 0.83 | 1.14 |
| Bn_63 | 1.25 | 0.31 | 0.93 | 0.69 | 0.89 | 1.15 |
| Bn_64 | 0.43 | 0.55 | 1.04 | 0.83 | 1.03 | 0.62 |
| Bn_67 | 0.30 | 0.64 | 0.70 | 0.86 | 0.81 | 1.41 |
| Bn_68 | 1.07 | 1.36 | 2.01 | 1.87 | 0.98 | 1.14 |
| Bn_07 | 1.19 | 0.45 | 2.27 | 1.67 | 1.04 | 0.85 |
| Bn_08 | 1.20 | 0.36 | 1.64 | 0.71 | 1.20 | 0.67 |
| Bn_09 | 0.80 | 1.08 | 0.84 | 1.20 | 1.07 | 0.96 |
| Fg | 1.46 | 1.67 | 1.48 | 1.16 | 0.84 | 1.00 |
| Rs | 0.61 | 0.42 | 0.64 | 0.50 | 0.91 | 0.70 |
| Ta_01 | 1.06 | 0.67 | 1.04 | 0.95 | 1.03 | 1.17 |
| Ta_10 | 1.66 | 1.20 | 2.25 | 3.01 | 0.97 | 1.40 |
| Ta_11 | 1.40 | 0.93 | 1.30 | 3.22 | 1.71 | 2.54 |
| Ta_12 | 1.03 | 0.35 | 1.50 | 0.63 | 1.41 | 0.95 |
| Ta_13 | 3.75 | 2.51 | 4.09 | 3.77 | 1.42 | 1.91 |
| Ta_14 | 0.43 | 0.99 | 0.50 | 0.78 | 0.60 | 0.53 |
| Ta_15 | 0.52 | 0.74 | 1.52 | 0.89 | 0.98 | 1.18 |
| Ta_16 | 1.25 | 0.66 | 1.36 | 0.72 | 1.09 | 0.85 |
| Ta_18 | 3.77 | 3.07 | 3.06 | 1.42 | 1.13 | 1.07 |
| Ta_19 | 2.16 | 1.24 | 2.32 | 1.91 | 1.23 | 1.14 |
| Ta_02 | 0.69 | 1.54 | 2.04 | 2.56 | 0.75 | 1.24 |
| Ta_20 | 1.23 | 0.78 | 1.40 | 0.57 | 1.06 | 0.92 |
| Ta_21 | 0.85 | 0.24 | 0.82 | 0.61 | 1.17 | 0.60 |
| Ta_22 | 0.97 | 0.53 | 1.10 | 0.51 | 0.88 | 0.84 |
| Ta_23 | 0.97 | 0.57 | 1.46 | 1.05 | 1.06 | 0.92 |
| Ta_24 | 2.97 | 2.43 | 4.75 | 4.90 | 1.91 | 3.25 |
| Ta_25 | 0.72 | 0.41 | 0.84 | 0.80 | 0.74 | 0.77 |
| Ta_26 | 0.90 | 0.61 | 0.93 | 0.83 | 1.00 | 1.05 |
| Ta_27 | 1.09 | 0.47 | 2.15 | 0.91 | 1.11 | 0.87 |
| Ta_28 | 1.16 | 0.61 | 2.29 | 0.78 | 1.26 | 0.90 |
| Ta_29 | 1.26 | 0.97 | 1.36 | 0.60 | 0.96 | 0.81 |
| Ta_03 | 0.44 | 0.48 | 0.63 | 0.65 | 0.74 | 0.81 |
| Ta_30 | 0.92 | 0.61 | 1.02 | 0.86 | 0.73 | 0.75 |
| Ta_31 | 1.28 | 1.88 | 1.25 | 1.62 | 1.40 | 0.91 |
| Ta_32 | 1.04 | 0.70 | 1.20 | 0.99 | 1.05 | 1.04 |
| Ta_33 | 0.53 | 0.71 | 0.92 | 1.10 | 0.89 | 0.76 |
| Ta_34 | 0.78 | 0.72 | 0.97 | 0.80 | 0.76 | 0.72 |
| Ta_35 | 0.76 | 0.64 | 0.91 | 0.83 | 0.92 | 0.69 |
| Ta_36 | 1.01 | 0.56 | 1.08 | 0.61 | 1.03 | 1.08 |
| Ta_37 | 2.19 | 1.26 | 1.58 | 2.31 | 1.64 | 3.28 |
| Ta_38 | 0.40 | 1.02 | 0.56 | 0.92 | 0.77 | 0.91 |
| Ta_39 | 2.75 | 2.22 | 2.78 | 0.83 | 0.97 | 0.81 |
| Ta_04 | 1.13 | 0.59 | 1.19 | 0.70 | 1.02 | 0.88 |
| Ta_40 | 1.02 | 0.99 | 1.07 | 0.74 | 0.64 | 0.80 |
| Ta_41 | 1.30 | 1.19 | 2.11 | 1.53 | 0.88 | 0.82 |
| Ta_05 | 0.97 | 0.70 | 1.16 | 1.21 | 1.21 | 1.20 |
| Ta_06 | 0.94 | 0.81 | 0.74 | 1.13 | 1.23 | 0.36 |
| Ta_07 | 0.92 | 0.68 | 1.30 | 0.71 | 1.23 | 0.80 |
| Ta_08 | 0.81 | 0.38 | 0.83 | 0.67 | 0.48 | 0.44 |
| Ta_09 | 3.32 | 2.08 | 4.02 | 4.31 | 1.86 | 1.74 |
